# Supplementary material for: Feeding response of the tropical copepod Acartia erythraea to short-term thermal stress: more animal-derived food was consumed
Source: PeerJ. 2018 Dec 19;6:e6129. doi: 10.7717/peerj.6129 (PMC6304158; doi:10.7717/peerj.6129)
Supplement: Data S1 [file peerj-06-6129-s001.docx]

primer 18S Non-copepodF2 (5’-agcaggcgcGhaaattrcccaatcy-3’) paired with 18S Non-copepodR2 (5’-CCGTGTTGAGTCAAATTAAGCCG-3’CGGCTTAATTTGACTCAACACGG)

>19-1

AGCAGGCGCGCAAATTGCCCAATCTCGACACGAGGAGGTAGTGACAAGAAATATTCAAGCAGGGCCCTCTGGGTCGTGCTATGAAAATGAGCTTAATGTAAACCGTTTCGCGAGGATCTATTGATGGGCAAGTCTGGTGCCAGCAGCCGCGGTAATTCCAGCATCAATAGCGTATATTAAAGTTGTTGCAGTTGAAACGCCCGTAGTTGAGACTCGACGCGGGCATGGATCGTGGCCCAAGGGCCGCCTTCCGCGCCTGCGTTTCCCGGGAGTCCTGTGCGGTGTCTCGCAAGGGATGTCGTGCATCTCCCGCGTTTACCTTGAGTAAATTGGAGTGTTCAAAGCAGGCGGGTGCTTGCACATGTTAGCATGGAATAATGGAACAGGGCGTATGTCCCTCCGTTGGTACCGGACAGACGCAATGACTAATAGGGACAGTTGGGGGCATTTGTATTGCGCCGCCAGAGGTGAAATTCGATGACTGGCGCAAGACAAACGAGTGCGAAAGCATTTGCCAAGGATGTTTTCATTAATCAAGGACGAAAGTATGGGGAGCCAAGACGATTAGATACCGTCGTAGTCTATACTGTAAACGATGCCGGCTCGGGATCAGAGGGCGGACACGAGCCCCCTCTGGCACCGTTCGAGAAATCAAAGCTTTTGGGCTCTGGGGGGAGTATGGTCGCAAGGCTGGAACTTAAAGGAATTGACGGAGGGGCACCACCAGACGTGGAGCCTGCGGCTTAATTTGACTCAACACGG

>19-2

AGCAGGCGCGTAAATTGCCCAATCCTGATACGGGGAGGTAGTGACAATAAATAACAATACGGGGCCCATCGGGTCTTGTAATTGGAATGAGTACAATCTAAATCCCTTAACGAGGATCAATTGGAGGGCAAGTCTGGTGCCAGCAGCCGCGGTAATTCCAGCTCCAATAGCGTATATTAAAGTTGTTGCAGTTAAAAAGCTCGTAGTTGGATTTCTGGTAGGAGCGACCGTTCCGAACTTGATTGTTCGTGTATTGAGTTGTCTCCAGCCATCCTTGTGGAGAACTTTTCTTGCATTAATTTGTAGGGATTGGGACCCGCATCGTTTACTGTGAAGAAATTAGAGTGTTTAAAGCAGGCAATCGCTTAAATACATTAGCATGGAATAATAAGATAGGACTTTGGTACTATTTTGTTGGTTTGCATACCAAATTAATGATCAACAGGAACAGTTTGAGGATATTCGTATGAACATGTCAGAGGTGAAATTCTTGGATTTTGATCAGACGAACTACTGCGAAAGCATTTATCAAGGATGTTTTCATTAATCAAGAACGAAAGTTAGGGGATCGAAGATGATTAGATACCATCGTAGTCTTAACCATAAACTATGCCGACTAGGGATTGGCGGACGTTGTCTATATGACTCCGCCAGCACCTCATGAGAAATCAAAGTCTTTGGGTTCCGGGGGGAGTATGGTCGCAAGGCTGAAACTTAAAGGAATTGACGGAAGGGCACCACCAGGAGTGGAGCCTGCGGCTTAATTTGACTCAACACGG

>19-4

AGCAGGCGCGTAAATTGCCCAATCTTAATTCAGGGAGGTAGTGACAATAAATAACAATACCGGGCTTCTTAAGTCTGGTAATTGGAATGAGTACAATCTAAATCCCTTAACGAGGATCCATTGGAGGGCAAGTCTGGTGCCAGCAGCCGCGGTAATTCCAGCTCCAATAGCGTATATTTAAGTTGTTGCAGTTAAAAAGCTCGTAGTTGGATTTCGGGTAGGATTTGCCGGTCCGTCTTTGAGATGTGTACTGGTAAGTCCTATCTTCTTGTCGGGGACCAGCTCCTGGGCTTCACTGTCCGGGACTGGGAGCCGACGAAGTTACTTTGAGTAAATTAGAGTGTTCAAAGCAAGCCTACGCTCTGAATATATTAGCATGGAATAACACGATAGGACTCTGGCTTATCTTGTTGGTCTGTGAGACCAGAGTAATGATTAAGAGGGACAGTCGGGGACATTCGTATTTCATTGTCAGAGGTGAAATTCTTGGATTTATGAAAGACGAACTTCTGCGAAAGCATTTGTCAAGGATGTTTTCATTAATCAAGAACGAAAGTTGGGGGCTCGAAGACGATTAGATACCGTCCTAGTCTCAACCATAAACGATGCCGACTAGGGATTGGCAGATGTTTTTTTGATGACTCTGCCAGCACCTTATGAGAAATCAAAGTTTTTGGGTTCCGGGGGGAGTATGGTCGCAAGGCTGAAACTTAAAGGAATTGACGGAAGGGCACCACCAGGCGTGGAGCCTGCGGCTTAATTTGACTCAACACGG

>19-9

AGCAGGCGCGTAAATTGCCCAATCCCGACACGGGGAGGTAGTGACGAAAAATAACAATACAGGACTCTTTCGAGGCCCTGTAATTGGAATGAGCACAATCTAAATCCTTTAGCGAGGATCTATTGGAGGGCAAGTCTGGTGCCAGCAGCCGCGGTAATTCCAGCTCCAATAGCGTATATTTAAGTTGTTGCAGTTAAAAAGCTCGTAGTTGGATTTCGGATGGGATCTGCCGGTCCGTCTTTGAGATGTGTACTGGTAGGTCCTATCTTGTTGTCGGGGACCAGCTCCTGGGCTTCACTGTCCGGGACTGGGAGCCGACGAAGTTACTTTGAGTAAATTAGAGTGTTCAAAGCAAGCCTACGCTCTGAATATATTAGCATGGAATAACACGATAGGACTCTGGCTTATCTTGTTGGTCTGTGAGACCAGAGTAATGATTAAGAGGGACAGTCGGGGACATTCGTATTTCATTGTCAGAGGTGAAATTCTTGGATTTATGAAAGACGAACTTCTGCGAAAGCATTTGTCAAGGATGTTTTCATTAATCAAGAACGAAAGTTGGGGGCTCGAAGACGATTAGATACCGTCCTAGTCTCAACCATAAACGATGCCGACTAGGGATTGGCAGATGTTTTTTTGATGACTCTGCCAGCACCTTATGAGAAATCAAAGTTTTTGGGTTCCGGGGGGAGTATGGTCGCAAGGCTGAAACTTAAAGGAATTGACGGAAGGGCACCACCAGGCGTGGAGCCTGCGGCTTAATTTGACTCAACACGG

>19-5

AGCAGGCGCGAAAATTGCCCAATCTTGACACAGGGAGGTAGTGACAATAAATAACAATGACGGGCCTTTGTAGGTCTGTCAATTGGAATGAGAACAATTTAAACCCCTTATCGAGTATCAATTGGAGGGCAAGTCTGGTGCCAGCAGCCGCGGTAATTCCAGCTCCAATAGCGTATATTAAAGTTGTTGCAGTTAAAAAGCTCGTAGTTGGATTTGTGGTTCGTCCCGCCGGCTGGGTCATTGACTTTTGCTGTGCTGGTCGTCCATCCTTGGGTGGAATCTGTGTGGCATTAGGTTGTCGCGCAGGGGATGCCCATCGTTTACTGTGAAAAAATTAGAGTGTTCAAAGCAGGCTTATGCCGTTGAATATATTAGCATGGAATAATGATATAGGACCTTGGTACTATTTTGTTGGTTTGCGCACTGAGGTAGTGATTAATAGGGACAGTTGGGGGTATTCGTATTCCATTGTAAGAGGTGAAATTCTTGGATTTTTGGAAGACGAACTACTGCGAAAGCATTTACCAAGGATGTTTTCATTAATCAAGAACGAAGGTTAGGGGATCGAAGATGATTAGATACCATCGTAGTCTTAACCATAAACTATGCCGACAAGGGATTGGCGGGGTCTCGTTACGTCCCTGTCAGCACCTTATGAGAAATCACAAGTTTTTGGGTTCCGGGGGAGTATGGTCGCAAGGCTGAAACTTAAAGAAATTGACGGAAGGGCACCACCAGGAGTGGAGCCTGCGGCTTAATTTGACTCAACACGG

>19-14

AGCAGGCGCGTAAATTGCCCAATCCTGACACAGGGAGGTAGTGACAATAAATAACAATGCCGGGCCTTTACAGGTCTGGCAATTGGAATGAGAACAATTTAAATCCCTTATCGAGTATCAATTGGAGGGCAAGTCTGGTGCCAGCAGCCGCGGTAATTCCAGCTCCAATAGCGCATATTAAAGTTGTTGCAGTTAAAAAGCTCGTAGTTGGATTTCTGGCAGGAGGGACCGGTCACGCACTCTGTGTGTGAACTTGGCTCCCTCTGGCCATCCTTGGGGAGATCCTGTTTGGCATTAAGTTGTCGGGCAGGGGATACCCATCGTTTACTGTGAAAAAATTAGAGTGTTTAAAGCAGGCTTATGCCGTTGAATATATTAGCATGGAATAATAGGATAGGACTTCGGAACTATTTTGTTGGTTTGCGTTACGAAGTAATGATTAATAGGGACAGTTGGGGGTATTCGTATTTCGTTGTCAGAGGTGAAATTCTTGGATTTCCGAAAGACGAACTACTGCGAAAGCATTTACCAAGGATGTTTTCATTAATCAAGAACGAAAGTTAGGGGATCGAAGATGATTAGATACCATCGTAGTCTTAACCATAAACTATGCCGACTCAGGATTGGCGGTTGTTTTTTGACTCCGTCAGCACTGTATGAGAAATCAAAGTCTTTGGGTTCCGGGGGGAGTATGGTCGCAAGGCTGAAACTTAAAGAAATTGACGGAAGGGCACCACCAGGAGTGGAACCTGCGGCTTAATTTGACTCAACACGG

>19-16

AGCAGGCGCGTAAATTGCCCAATCCTGACACAGGGAGGTAGTGACAATAAATAACAGTGCCGGGCCTTTACAGGTCTGGCAATTGGAATGAGAACAATTTAAATCCCTTATCAAGGATCAATTGGAGGGCAAGTCTGGTGCCAGCAGCCGCGGTAATTCCAGCTCCAATAGCGTATTTTAAAGTTGTTGCAGTTAAAAAAGCTTGTAGTTGGATTTGTGGTTAACATGAGATCTCTGTCCCTCCTGGGTTACAGTTTTGTCTTGTGTTGCCATCCTTGGGTGGAATCTGTGTGGCATTAGGTTGTCGTGCAGCCGACGCCCATCGTTTACTGTGAAAAAATTAGTGTTCAAAGCAGGCTTACACCGTTGAATATGTTAGCATGGAATAATGATATAGGACCTTGGTACTATTTTGTTGGTTTGCGCACTGAGGTAATGATTAAAAGGGTCAACTGGGGATATTCCTATTCCGCTGTCAGAGGTGAAATTCTTGGATTTTTGGAAGACGAACTACTGCGAAAGCATTTATCAAGGATGTTTTCATTAATCAAGAACGAAAGTTAGGGGATCGAAGATGATTAGATACCATCATAGTCTTAACCATAAACTATGCCGACAAGGGATTGGTGGAGTATCGTTACGTCTCTATCAGCACCTTAGGAGAAATCACAAGTCTTTGGGTTCTGGGGGGAGTATGGTCGCAAGGCTGAAACTTAAAGAAATTGACGGAAGGGCACCACCAGGAGTGGAGCCTGCGGCTTAATTTGACTCAACACGG

>19-17

AGCAGGCGCGTAAATTGCCCAATCTTGACACAGGGAGGTAGTGACAAGAAATAGCAATACAGGGCAATTTGTCTTGTAATTGGAATGAATAGAATTTAAATCCCTTTGTGAGTATCAATTGGAGGGCAAGTCTGGTGCCAGCAGCCGCGGTAATTCCAGCTCCAATAGCGTATATTAAAGTTGTTGCGGTTAAAAAGCTCGTAGTTGGATTTCTGCTGAGGACAGCTGATCCGCCCACTGGGTTGGTATTCTGCTTGTCCTTGGCATCTTCCTGAAGAGCGTGACTGCACTTCACTGTGTGGTGCGAAATTCAGGACATTTACTTTGAGGAAATTAGAGTGTTTCAAGCAAGCGCACGCCTTGAATACATTAGCATGGAATAATAAGATAGGACTTCGGTTCTATTTTGTTGGTTTCTAGGACTGAGGTAATGATTGATAGGGATAGTTGGGGGCATTCGTATTTAACTGTCAGAGGTGAAATTCTTGGATTTGTTAAAGACGGACTACTGCGAAAGCATTTGCCAAGGATGTTTTCATTGATCAAGAACGAAAGTTAGGGGATCGAAGACGATCAGATACCGTCCTAGTCTTAACCATAAACTATGCCGACTAGAGATTGGAGGTCGTTCCTCTTACGACTCCTTCAGCACCTTATGAGAAATCAAAGTCTTTGGGTTCCGGGGGGAGTATGGTCGCAAGGCTGAAACTTAAAGGAATTGACGGAAGGGCACCACCAGGAGTGGAGCCTGCGGCTTAATTTGACTCAACACGG

>19-18

AGCAGGCGCGTAAATTGCCCAATCTCGACTCGGGGAGGTAGTGACAATAAATAACAATGCCGGGCTATTGTAGTCTGGCAATTGGAATGAGTACAATCTAAATCCCTTAACGAGGAACAATTGGAGGGCAAGTCTGGTGCCAGCAGCCGCGGTAATTCCAGCTCCAATAGCGTATATTAAAGTTGTTGCAGTTAAAAAGCTCGTAGTTGGATTTCGGGGTGGCCTGCCTGGTCCACCGCAAGGTGAGTACTGGTCAGCCGCCCTTCCTCTCGAAAGCCCCGACTGCTCTTCATTGCAGTGGTCGGGTAGTTCGGGACGTTTACTTTGAAAAAATTAGAGTGTTCAAGGCAGGCTCTCGCCTGAATACATTAGCATGGAATAATGGAAGAGGACCTCGGTCCTATTTTGTTGGTTTCTAGGGCCGAAGTAATGATTAATAGGGACAGTTGGGGGTATTCGTATTTCGTTGTCAGAGGTGAAATTCTTGGATTTCCGAAAGACGAACTACTGCGAAAGCATTTACCAAGGATGTTTTCATTAATCAAGAACGAAAGTTAGGGGATCGAAGATGATTAGATACCATCGTAGTCTTAACCATAAACTATGCCGACTCAGGATTGGCGGTTGTTTTTTGACTCCGTCAGCACTGTATGAGAAATCAAAGTCTTTGGGTTCCGGGGGGAGTATGGTCGCAAGGCTGAAACTTAAAGAAATTGACGGAAGGGCACCACCAGGAGTGGAACCTGCGGCTTAATTTGACTCAACACGG

>19-20

AGCAGGCGCGTAAATTGCCCAATCCTGACACAGGGAGGTAGTGACAATAAATAACAATGCCGGGCCTTCTTAGGTCTGGCAATTGGAATGAGAACAATTTAAACCCCTTATCGAGTATCAATTGGAGGGCAAGTCTGGTGCCAGCAGCCGCGGTAATTCCAGCTCCAATAGCGTATATTAAAGTTGTTGCAGTTAAAAAGCTCGTAGTTGGATTTGTGGTGTGTCCAGTCGGCCCTTGCTCTTTGAGTGATTGTGCTGTACTGGTCTGCCATGTTTGGGTGGAATCTGTGTGGCATTAAGTTGTCGTGCAGGGGATGCCCATCGTTTACTGTGAAAAAATTAGAGTGTTCAAAGCAGGCTTATGCCGTTGAATATATTAGCATGGAATAATGATATAGGACCTTGGTACTATTTTGTTGGTTTGCGCACTAAGGTAATGATTAAGAGGGACAGTTGGGGGTATTTGTATTCCATTGTCAGAGGTGAAATTCTTAGATTTTTGGAAGACAAACTACTGCGAAAGCATTTACCAAGGATGTTTTCATTAATCAAGAACGAAAGTTAGGGGATCGAAGATGATTAGATACCATCGTAGTCTTAACCATAAACTATGCCGACAAAGGATTGGTGGAGTCTCGTTTCGTCTCCATCAGCACTTTGTGAGAAATCATAAGTCTTTGGGTTCCGGGGGGAGTATGGTCGCAAGGCTGAAACTTAAAGAAATTGACGGAAGGGCACCACCAGGAGTGGAGCCTGCGGCTTAATTTGACTCAACACGG

>19-24

AGCAGGCGCGCAAATTGCCCAATCTTGACACAGGGAGGTAGTGACAATAAATAACAATGCCGGGCCTTCTTAGGTCTGGCAATTGGAATGAGAACAATTTAAACCCCTTATCGAGTATCAATTGGAGGGCAAGTCTGGTGCCAGCAGCCGCGGTAATTCCAGCTCCAATAGCGTATATTAAAGTTGTTGCAGTTAAAAAGCTCGTAGTTGGATTTGTGGTGTGTCCAGTCGGCCTTTGCTCTTTGAGTGATTGTGCTGTACTGGTCTGCCATGTTTGGGTGGAATCTGTGTGGCATTAAGTTGTCGTGCAGGGGATGCCCATCGTTTACTGTGAAAAAATTAGAGTGTTCAAAGCAGGCTTATGCCGTTGAATATATTAGCATGGAATAATGATATAGGACCTTGGTACTATTTTGTTGGTTTGCGCACTAAGGTAATGATTAAGAGGGACAGTTGGGGGTATTTGTATTCCATTGTCAGAGGTGAAATTCTTAGATTTTTGGAAGACAAACTACTGCGAAAGCATTTACCAAGGATGTTTTCATTAATCAAGAACGAAAGTTAGGGGATCGAAGATGATTAGATACCATCGTAGTCTTAACCATAAACTATGCCGACAAAGGATTGGTGGAGTCTCGTTTCGTCTCCATCAGCACTTTGTGAGAAATCATAAGTCTTTGGGTTCCGGGGGGAGTATGGTCGCAAGGCTGAAACTTAAAGAAATTGACGGAAGGGCACCACCAGGAGTGGAGCCTGCGGCTTAATTTGACTCAACACGG

>19-21

AGCAGGCGCGCAAATTACCCAATCCCGACACGGGGAGGTAGTGACAAGAAATATGATGACTGTGCCCTTTGTGGCGGAGTTTTTCGAATGAGTGAAATGCAAATGCCTTTGCGAGTATCCATTGGAGGGCAAGTCTGGTGCCAGCAGCCGCGGTAATTCCAGCTCCAATAGCGTATATTAATGTTGTTGCAGTTAAAAAGCTCGTAGTTGGATCTCTGGCTTTGTTCGGACGGCCCGCCTTCGGGTTGAGTGCTGGAAGAACTAGCCTATTTTCCGGTTGGCGTCCTTTCTCTTTCTTTGAGCGAGGTTCGCGATCCGGATCGGTTACTTTGAAGAAATTTGAGTGTTCAAAGCAGGCTTACGCCGTGAAAATTGCAGCATGGAATAACAAGATAGGACTGTCGTGTTCCATTTTGTTGGTTCTGGGACCGTAGTAATGATTGATAGGGACAGTTGGGGGTGCTGCTATTCCGCGGCCAGAGGTGAAATTCTCAGATTCCCGGAAGAGCAACTTATGCGAAAGCATTCACCAAGGATGTCTTCTTTAATCAAGAACGAAAGTTGGGGGATCGAAGATGATTAGATACCATCGTAGTCTTAACCATAAACTATGCCGACAAGGGATTGGCGGAGTTTCGTTTTGTCTCCGTCAGCACCTTATGAGAAATCACAAGTCTTTGGGTTCCGGGGGGAGTATGGTCGCAAGGCTGAAACTTAAAGAAATTGACGGAAGGGCACCACCAGGAGTGGAGCCTGCGGCTTAATTTGACTCAACACGG

>19-22

AGCAGGCGCGAAAATTGCCCAATCTTGACACAGGGAGGTAGTGACAATAAATAACAATGCCGGGCCATCATTGGTCTGGCAATTGGAATGAGAACAATTTAAATCCCTTATCGAGGATCAATTGGAGGGCAAGTCTGGTGCCAGCAGCCGCGGTAATTCCAGCTCCAATAGCGTATATTAAAGTTGTTGCAGTTAAAAAGCTCGTAGTTGGATTTGTGGCGTACGGTGTGTTCCGGGCACTTGTTGTCTGAGTAACCCGCTGTTGCCATCCTTGGGTGGAACCTGTGTGGCATTAGGTTGTCGTGCAGGGGATGCCCATCATTTACTGTGAAAAAATTAGAGTGTTCAAAGCAGGCTTATGCCGTTGAATATATTAGCATGGAATAATGAGATAGGACTTTTTTGCTATTTTGTTGGTTTGCGCAAAGAGGTAATGATTAATAGGGACAGTTGGGGGTATTCGTATTCCATTGTCAGAGGTGAAATTCTTGGATTTTTGGAAGACGAACTACTGCGAAAGCATTTACCAAGGATGTTTTCATTAATCAAGAACGAAAGTTAGGGGATCGAAGATGATTAGATACCATCGTAGTCTTAACCATAAACTATGCCGACAAGGGATTGGTGGAGTCTCGTTTCGTCTCCATCAGCACTTTGTGAGAAATCATAAGTCTTTGGGTTCCGGGGGGAGTATGGTCGCAAGGCTGAAACTTAAAGAAATTGACGGAAGGGCACCACCAGGAGTGGAGCCTGCGGCTTAATTTGACTCAACACGG

>19-27

AGCAGGCGCGCAATTGCCCAATCTTGACACAGGGAGGTAGTGACAATAAATAACAATGCCGGGCCATCATTGGTCTGGCAATTGGAATGAGAACAATTTAAATCCCTTATCGAGGATCAATTGGAGGGCAAGTCTGGTGCCAGCAGCCGCGGTAATTCCAGCTCCAATAGCGTATATTAAAGTTGTTGCAGTTAAAAAGCTCGTAGTTGGATTTGTGGCGTACGGTGTGTTCCGGGCACTTGTTGTCTGAGTAACCCGCTGTTGCCATCCTTGGGTGGAACCTGTGTGGCATTAGGTTGTCGTGCAGGGGATGCCCATCATTTACTGTGAAAAAATTAGAGTGTTCAAAGCAGGCTTATGCCGTTGAATATATTAGCATGGAATAATGAGATAGGACTTTTTTGCTATTTTGTTGGTTTGCGCAAAGAGGTAATGATTAATAGGGACAGTTGGGGGTATTCGTATTCCATTGTCAGAGGTGAAATTCTTGGATTTTTGGAAGACGAACTACTGCGAAAGCATTTACCAAGGATGTTTTCATTAATCAAGAACGAAAGTTAGGGGATCGAAGATGATTAGATACCATCGTAGTCTTAACCATAAACTATGCCGACAAGGGATTGGTGGAGTCTCGTTATGTCTCCATCAGCACCTTATGAGAAATCACAAGTCTTTGGGTTCCGGGGGGAGTATGGTCGCAAGGCTGAAACTTAAAGAAATTGACGGAAGGGCACCACCAGGAGTGGAGCCTGCGGCTTAATTTGACTCAACACGG

>19-43

AGCAGGCGCGTAAATTGCCCAATCTTGACACAGGGAGGTAGTGACAATAAATAACAATGCCGGGCCATCATTGGTCTGGCAATTGGAATGAGAACAATTTAAATCCCTTATCGAGGATCAATTGGAGGGCAAGTCTGGTGCCAGCAGCCGCGGTAATTCCAGCTCCAATAGCGTATATTAAAGTTGTTGCAGTTAAAAAGCTCGTAGTTGGATTTGTGGCGTACGGTGTGTTCCGGGCACTTGTTGTCTGAGTAACCCGCTGTTGCCATCCTTGGGTGGAACCTGTGTGGCATTAGGTTGTCGTGCAGGGGATGCCCATCATTTACTGTGAAAAAATTAGAGTGTTCAAAGCAGGCTTATGCCGTTGAATATATTAGCATGGAATAATGAGATAGGACTTTTTTGCTGTTTTGTTGGTTTGCGCAAGAAGGTAATGATTAATAGGGACAGTTGGGGGTATTCGTATTCCATTGTCAGAGGTGAAATTCTTGGATTTTTGGAAGACGAACTACTGCGAAAGCATTTACCAAGGATGTTTTCATTAATCAAGAACGAAAGTTAGGGGATCGAAGATGATTAGATACCATCGTAGTCTTAACCATAAACTATGCCGACAAGGGATTGGCGGAGTTTCGTTTTGTCTCCGTCAGCACCTTATGAGAAATCACAAGTCTTTGGGTTCCGGGGGGAGTATGGTCGCAAGGCTGAAACTTAAAGGAATTGACGGAAGGGCACCACCAGGAGTGGAGCCTGCGGCTTAATTTGACTCAACACGG

>19-26

AGCAGGCGCGTAAATTGCCCAATCCTGACATAGGGAGGTAGTGACAAGAAATAACAATACAGGGCATCCATGTCTTGTAATTGGAATGAATGTGATTTAAACCCCTTTGTGAGTATCAATTGGAGGGCAAGTCTGGTGCCAGCAGCCGCGGTAATTCCAGCTCCAATAGCGTATATTAAAGTTGTTGCGGTTAAAAAGCTCGTAGTTGGATTTCTGCCGAGGACGACCGGTCCGCCTTTCGGGTGAGCATCTGGCTCGGCTTGGGCATATTCTTGGAGAACGTTCCTGCACTTGACTGTGCGGTGCGGTAGCCAAGAGCTTTACTTTGAGGAAATTAGAGTGTTCTAAGCAGGCAGATGCCTTGAATACATTAGCATGGAATAATGATGTAGGACTTCGGTTCTATTTTGTTGGTTTCTAGAGCTGAGGTAATGATTAATAGGGATGGTTGGGGGCATTCGTATTTAATTGTCAGAGGTGAAATTCTTGGATTTGTTAAAGACGGACGACTGCGATAGCATTTGCCAAGGATGTTTTCATTGATCAAGAACGAAAGTTAGGGGATCGAAGACGATCAGATACCGTCGTAGTCTTAACCATAAACCATGCCGACTAGAGATTGGAGGTCGTTTTCCACACGACCCCTTCAGCACCTTATGAGAAATCAAAGTCTTTGGGTTCCGGGGGGAGTATGGTCGCAAGGCTGAAACTTAAAGGAATTGACGGAAGGGCACCACCAGGAGTGGAGCCTGCGGCTTAATTTGACTCAACACGG

>19-29

AGCAGGCGCGCAAATTGCCCAATCTTGACACAGGGAGGTAGTGACAATAAATAACAATGCCGGGCCTTTAAAGGTCTGGCAATTGGAATGAGAACAATTTAAACCCCTTATCGAGGATCAATTGGAGGGCAAGTCTGGTGCCAGCAGCCGCGGTAATTCCAGCTCCAATAGCGTATATTAAAGTTGTTGCAGTTAAAAAGCTCGTAGTTGGATTTGTGGTTGAGGTGGCCGGCCCTCACTATGTGTGGTGCTTGGCTGTCCTCGTCCATCCTTGGGTGGAACTGGTGTGGCATTAAGTTGTCGTGTCAGGGATGCCCATCATTTACTGTGAAAAAATTAGAGTGTTCAAAGCAGGCTTACGCCGCTGAATATATTAGCATGGAATAATGATATAGGACCTTGGTACTATTTTGTTGGTTTGCGCACTAAGGTAATGATTAAGAGGGACAGTTGGGGGTATTTGTATTCCATTGTCAGAGGTGAAATTCTTAGATTTTTGGAAGACAAACTACTGCGAAAGCATTTACCAAGGATGTTTTCATTAATCAAGAACGAAAGTTAGGGGATCGAAGATGATTAGATACCATCGTAGTCTTAACCATAAACTATGCCGACAAAGGATTGGTGGAGTCTCGTTTCGTCTCCATCAGCACTTTGTGAGAAATCATAAGTCTTTGGGTTCCGGGGGGAGTATGGTCGCAAGGCTGAAACTTAAAGAAATTGACGGAAGGGCACCACCAGGAGTGGAGCCTGCGGCTTAATTTGACTCAACACGG

>19-39

AGCAGGCGCGTAAATTGCCCAATCCTGACACAGGGAGGTAGTGACAATAAATAACAATGCCGGGCCTTTGTAGGTCTGGCAATTGGAATGAGAACAATTTAAACCCCTTATCGAGTATCAATTGGAGGGCAAGTCTGGTGCCAGCAGCCGCGGTAATTCCAGCTCCAATAGCGTATATTAAAGTTGTTGCAGTTAAAAAGCTCGTAGTTGGATTTGTGGTGTACTTCGGCGGCCCGTCACAATGTGATGGAGTCTGCTGAAGTCGCCATCCTTGGGTGGATCCTGTGTGGCATTAAGTTGTCGTGCAGGGGATGCCCATCGTTTACTGTGAAAAAATTAGAGTGTTCAAAGCAGGCTTATGCCGTTGAATATATTAGCATGGAATAATAAGATAGGACCTTGGTACTATTTTGTTGGTTTGCGCACCAAGGTAATGATTAATAGGGACAGTTGGGGGTATTCGTATTTCATTGTCAGAGGTGAAATTCTTGGATTTTTGAAAGACGAACTACTGCGAAAGCATTTACCAAGGATGTTTTCATTAATCAAGAACGAAAGTTAGGGAATCGAAGATGATTAGATACCATCGTAGTCTTAACCATAAACTATGCCGACAAGGGATTGGCGGAGTTTCGTTATGTCTCCGTCAGCACCTTATGAGAAATCACAAGTCTTTGGGTTCCGGGGGGAGTATGGTCGCAAGGCTGAAACTTAAAGAAATTGACGGAAGGGCACCACCAGGAGTGGAGCCTGCGGCTTAATTTGACTCAACACGG

>19-40

AGCTTGCATGCCTGCAGGTCGACGATTCGAAAATTGCCCAATCCTGACACAGGGAGGTAGTGACAAGAAATAGCAACACAAAGCACTTGCTTTGATGTTTGCAATGGCAGGCGGTTACATCTCGCTTAGAGTATCAATTGGAGGGCAAGTCTGGTGCCAGCAGCCGCGGTAATTCCAGCTCCAATAGCGTATATTAAAGTTGTTGCAGTTAAAAAGCTCGTAGTTGAAATGATGGTTAGCGATCGGCATTTCCACAGGAAAACCTTTTGCTTGCCAGGCATCTCACGATGCTTAGTTACTTTGAGAAAAATAGAGTGTTTCAAGCGGGCATTTGCTGAAATAGTGTACCGTGGAATGACGAAAAGGCTGCATTTCCGTTTTTCAACTCTCGGAAATGCGGTGCGTGTTGAGAGGAACAGTCGGGGGTATTCGTATTGAACAGTCAGAGGTGAAATTCTTAGATTTGTTCAGGACGAACTAGTGCGAAAGCATCTACCAAGGATGTTTTCATTAATCAAGAACGAAAGTTAGGGGATCGAAGACGATCAGATACCGTCGTAGTCTTAACCATAAACTATGCCGACTAGAGATCGGTGGATATCAATCAGCTTCCTTCGGCATCTTATGAGAAATCAAAGTCTTTGGGTTCTGGGGGGAGTATGGTCGCAAGGCTGAAACTTAAAGGAATTGACGGAAGGGCACCACCAGGAGTGGAGCCTGCGGCTTAATTTGACTCAACACGG

>19-48

AGCAGGCGCGAAAATTGCCCAATCCCAATACGGGGAGGTAGTGACAATAAATAACAATATGGGGCATTTCAATGTCTTATAATTGGAATGGGTACAACATAAATCCCTTAACGAGGATCAATTGGAGGGCAAGTCTGGTGCCAGCAGCCGCGGTAATTCCAGCTCCAATAGCGTATATTAAAGTTGTTGCAGTTAAAAAGCTCGTAGTTGGATTTCTGATGTCTTTTTGCGTTTCCTTCTTTGCCAGCGAAATGCTGGTGTTGGTCGCAATGGATATCTTCTTTCCCTACAAATTTGTCGCTGAATCATTAGTTTGACTCGGTGGTGAATAGGGATCATTTACTGTGAAAAAATTAGAGTGTTCAAAGCAAGTATTATTTCTTGAATACATTAGCATGGAATAATAAGATAGGACTTTGGTAATATTTTGTTGGTTTGCATACCTTGGTAATGATTAATAGGAACATATTGGGGATATCTGTATTTTTTAGTCAGAGGTGAAATTCTTGGATTTAAGAAAGACAAACTACAGCGAAAGCATTTATCAAGGATGTTTTCATTGATCAAGAACGAAAGTTGGGGGATCGAAGATGATTAGATACCATCGTAGTCTCAACCATAAACTATGCCGACTTGGGATTGGAGGACGTGAATTTTTAATAACTCCTTCAGCACCACATGAGAAATCAAAGCCTTTGGGTTCCGGGGGGAGTATGGTCGCAAGGCTGAAACTTAAAGGAATTGACGGAAGGGCACACAAGTAGTGGAGCCTGCGGCTTAATTTGACTCAACACGG

>19-50

AGCAGGCGCGTAAATTGCCCAATCTTGACACAGGGAGGTAGTGACAATAAATAACAATGCCGGGCCTTTATAGGTCTGGCAATTGGAATGAGAACAATTTAAACCCCTTATCGAGTATCAATTGGAGGGCAAGTCTGGTGCCAGCAGCCGCGGTAATTCCAGCTCCAATAGCGTATATTAAAGTTGTTGCAGTTAAAAAGCTCGTAGTTGGATTTGTGGCGTTCGCGTGCGTCCCGGCATTCGTGCCGGTGTTTGCTAGCGTCGCCATCCTTGGGTGGAACTAGTGTGGCATTAAGTTGTCGTGCTGGGGATGCCCATCGTTTACTGTGAAAAAATTAGAGTGTTCAAAGCAGGCTTATGCCGTTGAATATATTAGCATGGAATAATAAGATAGGACCTTGGTACTATTTTGTTGGTTTGCGCACCGAGGTAATGATTAATAGGGACAGTTGGGGGTATTCGTATTTCATTGTCAGAGGTGAAATTCTTGGATTTCTGAAAGACGAACTACTGCGAAAGCATTTACCAAGGATGTTTTCATTAATCAAGAACGAAAGTTAGGGGATCGAAGATGATTAGATACCATCGTAGTCTTAACCATAAACTATGCCGACAAGGGATTGGTGGAGTTTCGTTATGTCTCCATCAGCACCTTATGAGAAATCACAAGTCTTTGGGTTCCGGGGGGAGTATGGTCGCAAGGCTGAAACTTAAAGAAATTGACGGAAGGGCACCACCAGGAGTGGAGCCTGCGGCTTAATTTGACTCAACACGG

>19-51

AGCAGGCGCGTAAATTGCCCAATCCTAATGCAGGGAGGTAGTGACAATAAATACTGACGCTGGACTCTACGAGTCTGGTAAAGAATGAGAACAGTTTAAAACCCTTATCGAGGACCCATTGGAGGGCAAGTCTGGTGCCAGCAGCCGCGGTAATTCCAGCTCCAATAGCGTATATTAAAGTTGTTGCAGTTAAAAAGCTCGTAGTTGGATTTGTGGTGTACTTCGGCGGCCCGTCACTATGTGATGGAGCTTGCTGAAGTCGCCATCCTTGGGTGGATCCTGTGTGGCATTAAGTTGTCGTGCAGGGGATGCCCATCGTTTACTGTGAAAAAATTAGAGTGTTCAAAGCAGGCAATCGCTTGAATACATTAGCATGGAATAATAAGATAGGATCTTCGTCTATTTTGTTGGTTGCGGACTGAGATAATGATTAATAGGGATAGTTGGGGGTGTCTGTATTGTGTTGTCAGAGGTGAAATTCTTAGATTTACACAAGACAAACTACTGCGAAAGCATTCACCAAGGATGTTTTCATTAATCAAGAACGAAAGTCAGGGGATCGAAGAGGATTAGATACCCTCGTAGTCTTGACCATAAACTATGCCGACTCGGGATTGGCAGACGATTGTTTCGACTCTGTCAGCACCGTATGAAAAATCAAAGTCTTTGGGTTCCGGGGGGAGTATGGTCGCAAGGCTGAAACTTAAAGGAATTGACGGAAGGGCACCACCAGGAGTGGAGCCTGCGGCTTAATTTGACTCAACACGG

>19-53

AGCAGGCGCGTAAATTGCCCAATCTCGACACAGGGAGGTAGTGACAATAAATAACAATGCCGGGCCTTTACAGGTCTGGCAATTGGAATGAGAACAATTTAAATCCCTTATCGAGTATCAATTGGAGGGCAAGTCTGGTGCCAGCAGCCGCGGTAATTCCAGCTCCAATAGCGTATATTAAAGTTGTTGCAGTTAAAAAGCTCGTAGTTGGATTTCTGGCAGGAGGGACCGGTCACGCACTCTGTGTGTGAACTTGGCTCCCTCTGGCCATCCTTGGGGAGATCCTGTTTGGCATTAAGTTGTCGGGCAGGGGATACCCATCGTTTACTGTGAAAAAATTAGAGTGTTTAAAGCAGGCTTATGCCGTTGAATATATTAGCATGGAATAATAAGATAGGACCTTGGTACTATTTTGTTGGTTTGCGCACCGAGGTAATGATTAATAGGGACAGTTGGGGGTATTCGTATTCCATTGTCAGAGGTGAAATTCTTGGATTTCTGGAAGACGAACTACTGCGAAAGCATTTACCAAGGATGTTTTCATTAATCAAGAACGAAAGTTAGGGGATCGAAGATGATTAGATACCATCGTAGTCTTAACCATAAACTATGCCGACAAGGGATTGGCGGGCGTTGTATTGACTCCGTCAGCACCTTATGAGAAATCACAAGTCTTTGGGTTCCGGGGGGAGTATGGTCGCAAGGCTGAAACTTAAAGAAATTGACGGAAGGGCACCACCAGGAGTGGAGCCTGCGGCTTAATTTGACTCAACACGG

>19-62

AGCAGGCGCGAAAATTGCCCAATCTCGGTACGGGGAGGTAGTGACGAAAAATAACAATACGGGTCTTTTTATAGGCCCCGTAATTGGAATGAGTACACTTTAAAAGCATTAACGAGTACCAATTGGAGGGCAAGTCTGGTGCCAGCAGCCGCGGTAATTCCAGCTCCAATAGCGTATATTAAAGTTGTTGCAGTTAAAAAGCTCGTAGTTGGATCTGAGGAGTGGAGCTTACGGTTGGTCGCATGACTACACTGTTGGCTCGGCCTACATAGTCGGTGATTGTTTTGGTGCTCTTAATTGAGTGCCTTAATTACCCGGCCACGTTTACTTTGAAAAAATTGGAGTGCTCAAATCAGGCCTACTGCCTGAACAGATGTGCATGGAATAATGGAAAAGGACTTCGGTTCTATTTTGTTGGTTTTCGGAACATGAAGTAATGATTAAGAGGGACAGACGGGGGCATTCGTATTGCGGTGGGAGAGGTGAAATTCTTGGATCATCGCAAGACGCCCTACAGCGAAAGCATTTGCCAAGAATGTTTCCATTAATCAAGAACGAAAGTCAGAGGTTCGAAGACGATCAGATACCGTCCTAGTTCTGACCATAAACGATGCCAACTGGCAATCCGTTGCGATTGCAAGTTCGATCCAACGGGCAGCCTCCGGGAAACCAAAGTCTTTGGGTTCCGGGGGAAGTATGGTTGCAAAGCTGAAACTTAAAGGAATTGACGGAAAGGCACCACAAGGAGTGGAGCCTGCGGCTTAATTTGACTCAACACGG

>19-63

AGCAGGCGCGTAAATTGCCCAATCTCGGTACGGGGAGGTAGTGACGAAAAATAACAATACGGGTCTTTTTATAGGCCCCGTAATTGGAATGAGTACACTTTAAAAGCATTAACGAGTACCAATTGGAGGGCAAGTCTGGTGCCAGCAGCCGCGGTAATTCCAGCTCCAATAGCGTATATTAAAGTTGTTGCAGTTAAAAAGCTCGTAGTTGGATCTGAGGAGTGGAGCTTACGGTTGGTCGCATGACTACACTGTTGGCTCGGCCTACATAGTCGGTGATTGTTTTGGTGCTCTTAATTGAGTGCCTTAATTACCCGGCCACGTTTACTTTGAAAAAATTGGAGTGCTCAAATCAGGCCTACTGCCTGAACAGATGTGCATGGAATAATGGAAAAGGACTTCGGTTCTATTTTGTTGGTTTTCGGAACATGAAGTAATGATTAAGAGGGACAGACGGGGGCATTCGTATTGCGGTGGGAGAGGTGAAATTCTTAGATCATCGCAAGACGCCCAACAGCGAAAGCATTTGCCAAGAATGTTTCCATTAATCAAGAACGAAAGTCAGAGGTTCGAAGACGATCAGATACCGTCCTAGTTCTGACCATAAACGATGCCAACTGGCAATCCGTTGCGATTGCATGTTCGATCCAACGGGCAGCCTCCGGGAAACCAAAGTCTGTGGGTTCCGGGGGAAGTATGGTTGCAAAGCTGAAACTTAAAGGAATTGACGGAAAGGCACCACAAGGAGTGGAGCCTGCGGCTTAATTTGACTCAACACGG

>19-65

AGCAGGCGCGCAAATTGCCCAATCTTGACACAGGGAGGTAGTGACAATAAATAACAATGCCGGGCCATCATTGGTCTGGCAATTGGAATGAGAACAATTTAAATCCCTTATCGAGGATCAATTGGAGGGCAAGTCTGGTGCCAGCAGCCGCGGTAATTCCAGCTCCAATAGCGTATATTAAAGTTGTTGCAGTTAAAAAGCTCGTAGTTGGATTTGTGGCGTACAGTGTGTTCCGGGCACTTGTCTGAGTAACCCGCTGTTGCCATCCTTGGGTGGAACCTGTGTGGCATTAGGTTGTCGTGCAGGGGATGCCCATCATTTACTGTGAAAAAATTAGAGTGTTCAAAGCAGGCTTATGCCGTTGAATATATTAGCATGGAATAATGAGATAGGACTTTTTTGCTATTTTGTCGGTTTGCGCAAGAAGGTAATGATTAATAGGGACAGTTGGGGGTATTCGTATTCCATTGTCAGAGGTGAAATTCTTAGATTTTTGGAAGACAAACTACTGCGAAAGCATTTACCAAGGATGTTTTCATTAATCAAGAACGAAAGTTAGGGGATCGAAGATGATTAGATACCATCGTAGTCTTAACCATAAACTATGCCGACAAGGGATTGGTGGAGTTTCGTTATGTCTCCATCAGCACCTTATGAGAAATCACAAGTCTTTGGGTTCCGGGGGGAGTATGGTCGCAAGGCTGAAACTTAAAGAAATTGACGGAAGGGCACCACCAGGAGTGGAGCCTGCGGCTTAATTTGACTCAACACGG

>19-67

AGCAGGCGCGTAAATTGCCCAATCCTGACACAGGGAGGTAGTGACAATAAATAACAATGCCGGGCCTTTAAAGGTCTGGCAATTGGAATGAGAACAATTTAAACCCCTTATCGAGGATCAATTGGAGGGCAAGTCTGGTGCCAGCAGCCGCGGTAATTCCAGCTCCAATAGCGTATATTAAAGTTGCTGCAGTTAAAAAGCTCGTAGTTGGATTTGTGGCGCGGCCGTCCGGCGGGGGTCCTTTACTGGTCCCGTTTGTCCTGGTTTTCGGCCGCTCCTTTCTCGGGTAGCGCGTGGCTGGCATTAGGTTGTCGGCCGCTTCGCGCCCGTCATTTACTGTGAAAAAATTAGAGTGTTCAAAGCAGGCTTAGGCCGTTGGATACATTAGCATGGAATAATGAGATAGGACCTTGGTACTATTTTGTTGGTTTGCGCACCAAGGTAATGATTAATAGGGACAGTTGGGGGTATTCGTATTTCATTGTCAGAGGTGAAATTCTTGGATTTTTGAAAGACGAACTACTGCGAAAGCATTTACCAAGGATGTTTTCATTAATCAAGAACGAAAGTTAGGGGATCGAAGATGATTAGATACCATCGTAGTCTTAACCATAAACTATGCCGACAAGGGATTGGCGGAGTTTCGTTATGTCTCCGTCAGCACCTTATGAGAAATCACAAGTCTTTGGGTTCCGGGGGGAGTATGGTCGCAAGGCTGAAACTTAAAGAAATTGACGGAAGGGCACCACCAGGAGTGGAGCCTGCGGCTTAATTTGACTCAACACGG

>19-70

AGCAGGCGCGTAAATTGCCCAATCCTGACACAGGGAGGTAGTGACAATAAATATCAATTCTGGGCCACATGGTCCGGTAATTGGAATGAGTACAATGTAAACGCCTTAACGAGGATCCATTGGAGGGCAAGTCTGGTGCCAGCAGCCGCGGTAATTCCAGCTCCAATAGCGTATATTTAAGTTGTTGCAGTTAAAAAGCTCGTAGTTGGATTTCGGGTGGGACGCAGCGGTCTCGCATTGCGTTTGTACTGCTGCAGCCCTCCTTCTTGCCGGGGGCGGCCTCTCGGACTTCACTGTCTGGGGGTCAGAATCGGCGATGTTACTTTGAGTAAATTAGAGTGTTCAAAGCAAGCTTACGCTCTGAATATAATAGCATGGGATAACACGACAGGACTCTGGCCTATCGTGTTGGTCTATAGGACCAGAGTAATGATTAAGAGGGACAGTCGGGGGCATTCGTATTCCGTTGTCAGAGGTGAAATTCTTGGATTTACGGAAGACGAACATCTGCGAAAGCATTTGCCAAGGATGTTTTCATTGATCAAGAACGAAAGTTGGGGGCTCGAAGACGATTAGATACCGTCGTAGTCTCAACCATAAACGATGCCGACTAGGGATTGGCGGGTGTTTTTTTGATGACTCCGCCAGCACCTCATGAGAAATCAAAGTTTTTGGGTTCCGGGGGGAGTATGGTCGCAAGGCTGAAACTTAAAGGAATTGACGGAAGGGCACCACCAGGCGTGGAGCCTGCGGCTTAATTTGACTCAACACGG

>19-72

AGCAGGCGCGCAAATTGCCCAATCCTGACACAGGGAGGTAGTGACAATAAATAACAATGCCGGGCCTTTACAGGTCTGGCAATTGGAATGAGAACAATTTAAATCCCTTATCGAGTATCAATTGGAGGGCAAGTCTGGTGCCAGCAGCCGCGGTAATTCCAGCTCCAATAGCGTATATTAAAGTTGTTGCAGTTAAAAAGCTCGTAGTTGGATTTCTGGCAGGAGGGACCGGTCACGCACTCTGTGTGTGAACTTGGCTCCCTCTGGCCATCCTTGGGGAGATCCTGTTTGGCATTAAGTTGTCGGGCAGGGGATACCCATCGTTTACTGTGAAAAAATTAGAGTGTTTAAAGCAGGCTTATGCCGTTGAATATATTAGCATGGAATAATAAGATAGGACTTCGGAACTATTTTGTTGGTTTGCGTTACGAAGTAATGATTAATAGGGACAGTTGGGGGTATTCGTATTTCGTTGTCAGAGGTGAAATTCTTGGATTTCCGAAAGACGAACTACTGCGAAAGCATTTACCAAGGATGTTTTCATTAATCAAGAACGAAAGTTAGGGGATCGAAGATGATTAGATACCATCGTAGTCTTAACCATAAACTATGCCGACTCAGGATTGGCGGTTGTTTTTTGACTCCGTCAGCACTGTATGAGAAATCAAAGTCTTTGGGTTCCGGGGGGAGTATGGTCGCAAGGCTGAAACTTAAAGAAATTGACGGAAGGGCACCACCAGGAGTGGAACCTGCGGCTTAATTTGACTCAACACGG

>19-75

AGCAGGCGCGTAAATTGCCCAATCCTGACACAGGGAGGTAGTGACAATAAATAACAATGCCGGGCCATCATTGGTCTGGCAATTGGAATGAGAACAATTTAAATCCCTTATCGAGGATCAATTGGAGGGCAAGTCTGGTGCCAGCAGCCGCGGTAATTCCAGCTCCAATAGCGTATATTAAAGTTGTTGCAGTTAAAAAGCTCGTAGTTGGATTTGTGGCGTACGGTGTGTTCCGGGCACTTGTTGTCTGAGTAACCTGCTGTTGCCATCCTTGGGTGGAACCTGTGTGGCATTAGGTTGTCGTGCAGGGGATGCCCATCATTTACTGTGAAAAAATTAGAGTGTTCAAAGCAGGCTTATGCCGTTGAATATATTAGCATGGAATAATGAGATAGGACTTTTTTGCTATTTTGTTGGTTTGCGCAAAGAGGTAATGATTAATAGGGACAGTTGGGGGTATTCGTATTCCATTGTCAGAGGTGAAATTCTTGGATTTTTGGAAGACGAACTACTGCGAAAGCATTTACCAAGGATGTTTTCATTAATCAAGAACGAAAGTTAGGGGATCGAAGATGATTAGATACCATCGTAGTCTTAACTATAAACTATGCCGACAAGGGATTGGTGGAGTTTCGTTATGTTTCCATCAGCACCTTATGAGAAATCACAAGTCTTTGGGTTCCGGGGGGAGTATGGTCGCAAGGCTGAAACTTAAAGAAATTGACGGAAGGGCACCACCAGGAGTGGAGCCTGCGGCTTAATTTGACTCAACACGG

>19-77

AGCAGGCGCGCAAATTGCCCAATCTCGGCACGGGGAGGTAGTGACGAAAAATAACAATACGGGTCTTTTTATAGGCCCCGTAATTGGAATGAGTACACTTTAAAAGCATTAACGAGTACCAATTGGAGGGCAAGTCTGGTGCCAGCAGCCGCGGTAATTCCAGCTCCAATAGCGTATATTAAAGTTGTTGCAGTTAAAAAGCTCGTAGTTGGATCTGAGGAGTGGAGCTTACGGTTGGTCGCATGACTACACTGTTGGCTCGGCCTGCATAGTCGGTGATTGCTTTGGTGCTCTTAATTGAGTGCCTTAACTGCCCGGCCACGTTTACTTTGAAAAAATTGGAGTGCTCAAATCAGGCCCACTGCCTGAACAGATGTGCATGGAATAATGGAAAAGGACTTCGGTTCTATTTTGTTGGTTTTCGGAACATGAAGTAATGATTAAGAGGGACAGACGGGGGCATTCGTATTGCGGTGGGAGAGGTGAAATTCTTGGATCATCGCAAGACGCCCTACAGCGAAAGCATTTGCCAAGAATGTTTCCATTAATCAAGAACGAAAGTCAGAGGTTCGAAGACGATCAGATACCGTCCTAGTTCTGACCATAAACGATGCCAACTGGCAATCCGTTGCGATTGCAAGTTCGATCCAACGGGCAGCCTCCGGGAAACCAAAGTCTTTGGGTTCCGGGGGAAGTATGGTTGCAAAGCTGAAACTTAAAGGAATTGACGGAAAGGCACCACAAGGAGTGGAGCCTGCGGCTTAATTTGACTCAACACGG

>19-79

AGCAGGCGCGTAAATTGCCCAATCCTGACACAGGGAGGTAGTGACAATAAATAACAATGCCGGGCCTTTCTAGGTCTGGCAATTGGAATGAGAACAATTTAAATCCCTTATCGAGGATCAATTGGAGGGCAAGTCTGGTGCCAGCGGCCGCGGTAATTCCAGCTCCAATAGCGTATATTAAAGTTGTTGCAGTTAAAAAGCTCGTAGTTGGATTTGTGGCGTACGGTGGGTTCAGGGCACTTGTTGTCTGAGTAACTTGCTGTTGCCATCCTTGGGTGGAATCTGTGTGGCATTAGGTTGTCGTGCAGGGGATGCCCATCGTTTACTGTGAAAAAATTAGAGTGTTCAAAGCAGGCTTATGCCGTTGAATATATTAGCATGGAATAATGAGATAGGACTTTTTTGCTATTTTGTTGGTTTGCGCGAAGAGGTAATGATTAATAGGGACAGTTGGGGGTATTCGTATTCCATTGTCAGAGGTGAAATTCTTGAATTTTTGGAAGACGAACTACTGCGAAAGCATTTACCAAGGATGTTTTCATTAATCAAGAACGAAAGTTAGGGGATCGAAGATGATTAGATACCATCGTAGTCTTAACCATAAACTATGCCGACAAGGGATTGGCGGAGTTTCGTTATGTCTCCGTCAGCACCTTATGAGAAATCACAAGTCTTTGGGTTCCGGGGGGAGTATGGTCGCAAGGCTGAAACTTAAAGAAATTGACGGAAGGGCACCACCAGGAGTGGAGCCTGCGGCTTAATTTGACTCAACACGG

>19-81

AGCAGGCGCGAAAATTGCCCAATCCCGGCACGGGGAGGTAGTGACGAAAAATACCGATGCGGGGCCCCCCGCGGGGCTCTCGTAATCTGGACTGAGTACACTCTAAAGCCTTGCTTAGCGAGGATCTACTGGAGGGCAAGTCTGGTGCCAGCAGCCGCGGTAATTCCAGCTCCAGCAGCGTATACTAAAGTTGTTGCAGTTAAAAAGCTCGTAGTCGGATGTCGGTGTAAGGCGTTACGCCGTGGGCTTCGTCGGTGCGCTCGCATCTGCGGGCGGCTACTGACGGGTCCGACCTTGCGGCTCGCGGTCCCCGTCCGCGGGGGCTGGCGGGTTGCTGTTACTTTGAAAAAATGAGAGTGTTCAAGACGGGCTATTGTCGCTCGAATTAGAAAGCATGGAATAATGGAAGATGATCCGGCCTTCCGCCGTTTCCGTCGTGTTCCTTCGGGGGTGCGGTCGAGAGCGGCGTGAATGTTGCGGGATAATGATGAAGAGAGACGGTCGGGGGCATTCGTATTGCGGCGCGAGAGGTGAAATTCTTGGACCGTCGCAAGACGAACTGGTGCGAAAGCGTTTGCCAAGGACGTTTTCATTGATCGAGAGCGAAAGTCAGAGGCTCGAAGACGATCAGATACCGTCGTAGTTCTGACCGTAAACGATGTCGACCGGGGCTCGGCGGACCCGTCGTTGTCTCGAATGCAGAGACTGGTGACACCGCCGGTACCCTCCCCCTCGGTTCGGGAAACCTTTGAGTGTTTGGATTCCGGGGGGAGTATGGTTGCAAAGCTGAAACTTAAAGGAATTGACGGAAGGGCACCACCAGGAGTGGAGCCTGCGGCTTAATTTGACTCAACACGG

>19-83

AGCAGGCGCGCAAATTGCCCAATCCTGACACAGGGAGGTAGTGACAATAAATAACAATGCCGGGCCTTTATAGGTCTGGCAATTGGAATGAGAACAATTTAAATCCCTTATCGAGGATCAATTGGAGGGCAAGTCTGGTGCCAGCAGCCGCGGTAATTCCAGCTCCAATAGCGTATATTAAAGTTGTTGCAGTTAAAAAGCTCGTAGTTGGATTTGTGGCGTACGGTGTGCTCCGGGCACTTGTTGTCTGAGTAGCCTGCCGTTGCCATCCTTGGGTGGAACCTGTGTGGCATTAGGTTGTCGCGCAGGGGATGCCCATCGTTTACTGTGAAAAAATTAGAGTGTTCAAAGCAGGCTTATGCCGTTGAATATATTAGCATGGAATAATGAGATAGGACTTTTTTGCTATTTTGTTGGTTTGCGCAAAGAGGTAATGATTAATAGGGACAGTTGGGGGTATTCGTATTCCATTGTCAGAGGTGAAATTCTTGGATTTTTGGAAGACGAACTACTGCGAAAGCATTTACCAAGGATGTTTTCATTAATCAAGAACGAAAGTTAGGGGATCGAAGATGATTAGATACCATCGTAGTCTTAACCATAAACTATGCCGACAAGGGATTGGTGGAGTCTCGTTATGTCTCCATCAGCACCTTATGAGAAATCACAAGTCTTTGGGTTCCGGGGGGAGTATGGTCGCAAGGCTGAAACTTAAAGAAATTGACGGAAGGGCACCACCAGGAGTGGAGCCTGCGGCTTAATTTGACTCAACACGG

>19-85

AGCAGGCGCGTAAATTGCCCAATCTTGACACAGGGAGGTAGTGACAATAAATAACAATGCCGGGCCTTTGTAGGTCTGGCAATTGGAATGAGAACAATTTAAACCCCTTATCGAGTATCAATTGGAGGGCAAGTCTGGTGCCAGCAGCCGCGGTAATTCCAGCTCCAATAGCGTATATTAAAGTTGTTGCAGTTAAAAAGCTCGTAGTTGGATTTGTGGCTGTCACGGGCGGCCTATCACTTAGTGTTAGTGCTTGCCTGTGTCGCCATCCTTGGGTGGAGCCCGTGTGGCATTAGGTTGTCGTGCGGGGGATGCCCATCGTTTACTGTGAGAAAATTAGAGTGTTCAAAGCAGGCTTATGCCGTTGAATATGTTAGCATGGAATAATAAGATAGGACCTCGGTACTATTTTGTTGGTTTGCGCACCGAGGTAATGATTAATAGGGACAGTTGGGGGTATTCGTATTTCATTGTCAGAGGTGAAATTCTTGGATTTCTGAAAGACGAACTACTGCGAAAGCATTTACCAAGGATGTTTTCATTAATCAAGAACGAAAGTTAGGGGATCGAAGATGATTAGATACCATCGTAGTCTTAACCATAAACTATGCCGACAAGGGATTGGCGGAGTTTCGTTTTGTCTCCGTCAGCACCTTATGAGAAATCACAAGTCTTTGGGTTCCGGGGGGAGTATGGTCGCAAGGCTGAAACTTAAAGAAATTGACGGAAGGGCACCACCAGGAGTGGAGCCTGCGGCTTAATTTGACTCAACACGG

>19-87

AGCAGGCGCGAAAATTGCCCAATCTCCACTCGGGGGAGGTAATGACAAGAAATAACGAATCAGTGCTCTTTGAGTTCTGTATTCGGAATGAGTGCAATCTAAAAATCCCAATGAGGAACTATTGGAGGGCAAGTCTGGTGCCAGCAGCCGCGGTAATTCCAGCTCCAATAGCGTATATTAAAGTTGTTGCAGTTAAAAAGCTCGTAGTTGGATTTGTGGTGTACTTCGGCGGCCCGTCACAATGTGATGGAGTCTGCTGAAGTCGCCATCCTTGGGTGGATCCTGTGTGGCATTAAGTTGTCGTGCAGGGGATGCCCATCGTTTACTGTGAAAAAATTAGAGTGTTCAAAGCAGGCTTATGCCGTTGAATATATTAGCATGGAATAATAAGATAGGACCTTGGTACTATTTTGTTGGTTTGCGCACCAAGGTAATGATTAATAGGGACAGTTGGGGGTATTCGTATTCCATTGTCAGAGGTGAAATTCTTGGATTTTTGGAAGACGAACTACTGCGAAAGCATTTACCAAGGATGTTTTCATTAATCAAGAACGAAAGTTAGGGGATCGAAGATGATTAGATACCATCGTAGTCTTAACCATAAACTATGCCGACAAGGGATTGGCGGAGTTACGTTATGTCTCCGTCAGCACCTTATGAGAAATCACAAGTCTTTGGGTTCCGGGGGGAGTATGGTCGCAAGGCTGAAACTTAAAGAAATTGACGGAAGGGCACCACCAGGAGTGGAGCCTGCGGCTTAATTTGACTCAACACGG

>19-89

AGCAGGCGCGTAAATTGCCCAATCCTGACACAGGGAGGTAGTGACAATAAATAACAATGCCGGGCCTTTACAGGTCTGGCAATTGGAATGAGAACAATTTAAATCCCTTATCGAGTATCAATTGGAGGGCAAGTCTGGTGCCAGCAGCCGCGGTAATTCCAGCTCCAATAGCGTATATTAAAGTTGTTGCAGTTAAAAAGCTCGTAGTTGGATTTGTGGCTGTCACGGGCGGCCTATCACTTAGTGTTAGTGCTTGCCTGTGTCGCCATCCTTGGGTGGAGCCCGTGTGGCATTAGGTTGTCGTGCGGGGGATGCCCATCGTTTACTGTGAGAAAATTAGAGTGTTCAAAGCAGGCTTATGCCGTTGAATATGTTAGCATGGAATAATAAGATAGGACCTCGGTACTATTTTGTTGGTTTGCGCACCGAGGTAATGATTAATAGGGACAGTTGGGGGTATTCGTATTTCATTGTCAGAGGTGAAATTCTTGGATTTCTGAAAGACGAACTACTGCGAAAGCATTTACCAAGGATGTTTTCATTAATCAAGAACGAAAGTTAGGGGATCGAAGATGATTAGATACTATCGTAGTCTTAACCATAAACTATGCCGACAAGGGATTGGCGGAGTTTCGTTTTGTCTCCGTCAGCACCTTATGAGAAATCACAAGTCTTTGGGTTCCGGGGGGAGTATGGTCGCAAGGCTGAAACTTAAAGAAATTGACGGAAGGGCACCACCAGGAGTGGAGCCTGCGGCTTAATTTGACTCAACACGG

>19-90

AGCAGGCGCGAAAATTGCCCAATCCTGACACAGGGAGGTAGTGACAATAAATAACAATGCCGGGCCTTTATAGGTCTGGCAATTGGAATGAGAACAATTTAAATCCCTTATCGAGGATCAATTGGAGGGCAAGTCTGGTGCCAGCAGCCGCGGTAATTCCAGCTCCAATAGCGTATATTAAAGTTGTTGCAGTTAAAAAGCTCGTAGTTGGATTTGTGGCGTACGGTGTGTTCCGGGCACTTGTTGTCTGAGTAACCTGCTGTTGCCATCCTTGGGTGGAACCTGTGTGGCATTAGGTTGTCGTGCAGGGGATGCCCATCATTTACTGTGAAAAAATTAGAGTGTTCAAAGCAGGCTTATGCCGTTGAATATATTAGCATGGAATAATGAGATAGGACTTTTTTGCTATTTTGTTGGTTTGCGCAAAGAGGTAATGATTAATAGGGACAGTTGGGGGTATTCGTATTCCATTGTCAGAGGTGAAATTCTTGGATTTTTGGAAGACGAACTACTGCGAAAGCATTTACCAAGGATGTTTTCATTAATCAAGAACGAAAGTTAGGGGATCGAAGATGATTAGATACCATCGTAGTCTTAACCATAAACTATGCCGACAAGGGATTGGTGGAGTCTCGTTATGTCTCCATCAGCACCTTATGAGAAATCACAAGTCTTTGGGTTCCGGGGGGAGTATGGTCGCAAGGCTGAAACTTAAAGAAATTGACGGAAGGGCACCACCAGGAGTGGAGCCTGCGGCTTAATTTGACTCAACACGG

>19-92

AGCAGGCGCGTAAATTGCCCAATCCTGACACAGGGAGGTAGTGACAATAAATAACAATGCCGGGCCTTTGTAGGTCTGGCAATTGGAATGAGAACAATTTAAACCCCTTATCGAGTATCGATTGGAGGGCAAGTCTGGTGCCAGCAGCCGCGGTAATTCCAGCTCCAATAGCGTATATTAAAGTTGTTGCAGTTAAAAAGCTCGTAGTTGGATTTGTGGTGTACTTCGGCGGCCCGTCACAATGTGATGGAGTCTGCTGAAGTCGCCATCCTTGGGTGGATCCTGTGTGGCATTAAGTTGTCGTGCAGGGGATGCCCATCGTTTACTGTGAAAAAATTAGAGTGTTCAAAGCAGGCTTATGCCGTTGAATATATTAGCATGGAATAATGAGATAGGACTTTTTTGCTATTTTGTTGGTTTGCGCAAGAAGGTAATGATTAATAGGGACAGTTGGGGGTATTCGTATTCCATTGTCAGAGGTGAAATTCTTGGATTTTTGGAAGACGAACTACTGTGAAAGCATTTACCAAGGATGTTTTCATTAATCAAGAACGAAAGTTAGGGGATCGAAGATGATTAGATACCATCGTAGTCTTAACCATAAACTATGCCGACAAGGGATTGGTGGAGTCTCGTTATGTCTCCATCAGCACCTTATGAGAAATCACAAGTCTTTGGGTTCCGGGGGGAGTATGGTCGCAAGGCTGAAACTTAAAGAAATTGACGGAAGGGCACCACCAGGAGTGGAGCCTGCGGCTTAATTTGACTCAACACGG

>19-95

AGCAGGCGCGTAAATTGCCCAATCTTGACACAGGGAGGTAGTGACAAGAAATAACAATACAGGGCATCCATGTCTTGTAATTGGAATGAGTAGAATTTAAATCCCTTTTCGAGTATCAATTGGAGGGCAAGTCTGGTGCCAGCAGCCGCGGTAATTCCAGCTCCAATAGCGTATATTAAAGTTGTTGCGGTTAAAAAGCTCGTAGTTGGATTTCTGCCGAGGACGACCGGTCCGCCCTCTGGGTGTGTATCTGGTTTGGCCTGGGCATCTTCTTGGAGAACGTAGCTGCACTTGACTGTGTGGTGCGGTGTCCAGGACTTTTACTTTGAGGAAATTAGAGTGTTTCAAGCAGGCACACGCCTTGAATACATTAGCATGGAATAATAAGATAGGGCCTCGGTTCTATTTTGTTGGTTTCTAGAGCTGAGGTAATGATTAATAGGGATAGTTGGGGGCATTCGTATTTAACTGTCAGAGGTGAAATTCTTGGATTTGTTAAAGACGGACTACTGCGAAAGCATTTGCCAAGGATGTTTTCATTGATCAAGAACGAAAGTTAGGGGATCGAAGACGATCAGATACCGTCCTAGTCTTAACCATAAACCATGCCGACTAGAGATTGGAGGTCGTTATTCTTACGACTCCTTCAGCACCTTATGAGAAATCAAAGTCTTTGGGTTCCGGGGGGAGTATGGTCGCAAGGCTGAAACTTAAAGGAATTGACGGAAGGGCACCACCAGGAGTGGAGCCTGCGGCTTAATTTGACTCAACACGG

>19-96

AGCAGGCGCGTAAATTGCCCAATCCTGACACAGGGAGGTAGTGACAATAAATAACAATGCCGGGCCATCATTGGTCTGGCAATTGGAATGAGAACAATTTAAATCCCTTATCGAGGATCAATTGGAGGGCAAGTCTGGTGCCAGCAGCCGCGGTAATTCCAGCTCCAATAGCGTATATTAAAGTTGTTGCAGTTAAAAAGCTCGTAGTTGGATTTGTGGCGTACGGTGTGTTCCGGGCACTTGTTGTCTGAGTAACCTGCTGTTGCCATCCTTGGGTGGAACCTGTGTGGCATTAGGTTGTCGTGCAGGGGATGCCCATCATTTACTGTGAAAAAATTAGAGTGTTCAAAGCAGGCTTATGCCGTTGAATATATTAGCATGGAATAATGAGATAGGACTTTTTTGCTATTTTGTTGGTTTGCGCAAAGAGGTAATGATTAATAGGGACAGTTGGGGGTATTCGTATTCCATTGTCAGAGGTGAAATTCTTGGATTTTTGGAAGACGAACTACTGCGAAAGCATTTACCAAGGATGTTTTCATTAATCAAGAACGAAAGTTAGGGGATCGAAGATGATTAGATACCATCGTAGTCTTAACCATAAACTATGCCGACAAGGGATTGGTGGAGTCTCGTTATGTCTCCATCAGCACCTTATGAGAAATCACAAGTCTTTGGGTTCCGGGGGGAGTATGGTCGCAAGGCTGAAACTTAAAGAAATTGACGGAAGGGCACCACCAGGAGTGGAGCCTGCGGCTTAATTTGACTCAACACGG

>19-99

AGCAGGCGCGCAAATTGCCCAATCCTATACTAGGGAGGTAGTGACAAGAAATAACAACATAGATATTATATATTTATGATTGGAATGAAAGGACTTTAAAACATTTCTTGAGGATCCATTGGAGGGCAAGTCTGGTGCCAGCAGCCGCGGTAATTCCAGCTCCAACAGCGTATATTAAAGTTGCTGCAGTTAAAACGCTCGTAGTTGTTGTCCAACTATACGCTATTTCAAGCAATTGATAATAGTTCAATAGTTTAGCGGAAACTCGTTTCTGCCAAAACCTTGAGGAAAGATTAGTGTTCCAAGCAATTATCACTTTGTCCTCCCCAGCATGGTATTCAACCTAATAGAAAGGGATTGTCGAGTCTCTTTCTTACTATATAGAAATTCAAGGGGGTATACGTACTTTAATGTCAGAGGTGAAATTCATAGATTTTTAAAAGACGGTCAATTGCGAAAGCATTCACCAAGCGACTTTTCTTTGATCAAGAACGAAAGTTAGGGGATTGAAGACGATCAGATACCGTCGTAATCTTAACCGTAAACTATGCCGACTTGTCGCAGGTAGACCTTTCCTTCGAGTTTATCGTCGACTTTTGAGAAATCAAAGTTTTTGGGTTCTGGGGGGAGTATGATTGCAAGATTGAAACTTAAAGGAATTGACGGAAGGGCACCACCAGGAGTGGAGCTTGCGGCTTAATTTGACTCAACACGG

>19-100

AGCAGGCGCGAAAATTGCCCAATCCTGACACAGGGAGGTAGTGACAATAAATAACAATGCCGGGCCATCATTGGTCTGGCAATTGGAATGAGAACAATTTAAATCCCTTATCGAGGATCAATTGGAGGGCAAGTCTGGTGCCAGCAGCCGCGGTAATTCCAGCTCCAATAGCGTATATTAAAGTTGTTGCAGTTAAAAAGCTCGTAGTTGGATTTGTGGCGTACGGTGTGTTCCGGGCACTTGTTGTCTGAGTAACCTGCTGTTGCCATCCTTGGGTGGAACCTGTGTGGCATTAGGTTGTCGTGCAGGGGATGCCCATCATTTACTGTGAAAAAATTAGAGTGTTCAAAGCAGGCTTATGCCGTTGAATATATTAGCATGGAATAATGAGATAGGACTTTTTTGCTATTTTGTTGGTTTGCGCAAAGAGGTAATGATTAATAGGGACAGTTGGGGGTATTCGTATTCCATTGTCAGAGGTGAAATTCTTGGATTTTTGGAAGACGAACTACTGCGAAAGCATTTACCAAGGATGTTTTCATTAATCAAGAACGAAAGTTAGGGGATCGAAGATGATTAGATACCATCGTAGTCTTAACCATAAACTATGCCGACAAGGGATTGGTGGAGTCTCGTTATGTCTCCATCAGCACCTTATGAGAAATCACAAGTCTTTGGGTTCCGGGGGGAGTATGGTCGCAAGGCTGAAACTTAAAGAAATTGACGGAAGGGCACCACCAGGAGTGGAGCCTGCGGCTTAATTTGACTCAACACGG

>19-102

AGCAGGCGCGTAAATTGCCCAATCCTGACACAGGGAGGTAGTGACAATAAATAACAATGCCGGGCCTTTCTAGGTCTGGCAATTGGAATGAGAACAATTTAAATCCCTTATCGAGGATCAATTGGAGGGCAAGTCTGGTGCCAGCAGCCGCGGTAATTCCAGCTCCAATAGCGTATATTAAAGTTGTTGCAGTTAAAAAGCTCGTAGTTGAAATGATGGCTAGCGATTGGATTTCCATCGGATAACCTCTTGCTTGCCAGGCATCTCAAGATGCTTAGTTACTTTGAGAAAAATAGAGTGTTTCAAGCGGGCATTTGCTGAAATAGTGTACCGTGGAATGACGTAAAGGCTGCCTTTCCGCTTGTCGACTCTCGGAATTGCGGTGCGTGTTGAGAGGAACAGTCGGGGGTATTCGTATTGAACAGTCAGAGGTGAAATTCTTAGATTTGTTCAGGACGAACTAGTGCGAAAGCATCTACCAAGGATGTTTCCATTAATCAAGAACGAAAGTTAGGGGATCGAAGACGATCAGATACCGTCGTAGTCTTAACCATAAACTATGCCGACTAGAGATCGGTGGATAACACTCAGTTTCCTTCGGCATCTTATGAGAAATCAAAGTCTTTGGGTTCTGGGGGGAGTATGGTCGCAAGGCTGAAACTTAAAGGAATTGACGGAAGGGCACCACCAGGAGTGGAGCCTGCGGCTTAATTTGACTCAACACGG

>19-104

CGCCGCAGCCGAACGACCGAGCGCAGCGAGTCAGTGAGCGAGGAAGCGGAAGAGCGCCCAATACGCAAACCGCCTCTCCCCGCGCGTTGGCCGATTCATTAATGCAGCTGGCACGACAGGTTTCCCGACTGGAAAGCGGGCAGTGAGCGCAACGCAATTAATGTGAGTTAGCTCACTCATTAGGCACCCCAGGCTTTACACTTTATGCTTCCGGCTCGTATGTTGTGTGGAATTGTGAGCGGATAACAATTTCACACAGGAAACAGCTATGACCATGATTACGAATTCGAGCTCGGTACCCGGGGATCCTCTAGAGATTAAAGTTGTTGCAGTTAAAAAGCTCGTAGTTGGATTTCTGGCAGGTGGAGCGGGCCACACACTCTGTGCGTGAGCTTTGTCTCTCACTGGCCATCCTCGGGGAGATCCTGTTTGGCATTAAGTTGTCGGGCAGGGGATACCCGTCGTTTACTGCGAAAAAATTAGAGTGTTTAAAGCAGGCTTATGCCGTTGAATATATTAGCATGGGATAATAAGATAGGACTTCGGAACTATTTTGTTGGTTTGCGTTACGAAGTAATGATTAATAGGGACAGTTGGGGGTATTCGTATTTCATTGTCAGAGGTGAAATTCTTGGATTTCTGAAAGACGAACTACTGCGAAAGCATTTACCAAGGATGTTTTCATTAATCAAGAACGAAAGTTAGGGGATCGAAGACGATCAGATACCGTCCTAGTCTTAACCATAAACCATGCCGACTAGGGATTGGAGGTCGTTATTGATACGACCCCTTCAGCATCTTATGAGAAATCAAAGTCTTTGGGTTCCGGGGGGAGTATGGTCGCAAGGCTGAAACTTAAAGGAATTGACGGAAGGGCACCACCAGGAGTGGAGCCTGCGGCTTAATTTGACTCAACACGG

>19-107

AGCAGGCGCGCAAATTACCCAATCCTGACACAGGGAGGTAGTGACAATAAATAACAATGCCGGGCCTTTGTAGGTCTGGCAATTGGAATGAGAACAATTTAAACCCCTTATCGAGTATCAATTGGAGGGCAAGTCTGGTGCCAGCAGCCGCGGTAATTCCAGCTCCAATAGCGTATATTAAAGTTGTTGCAGTTAAAAAGCTCGTAGTTGGATTTGTGGTGTACTTCGGCGGCCCGTCACTATGTGATGGAGCTTGCTGAAGTCGCCATCCTTGGGTGAATCCTGTGTGGCATTAAGTTGTCGTGCAGGGGATGCCCATCGTTTACTGTGAAAAAATTAGAGTGTTCAAAGCAGGCTTATGCCGTTGAATATATTAGCATGGAATAATAAGATAGGACCTTGGTACTATTTTGTTGGTTTGCGCACCAAGGTAATGATTAATAGGGACAGTTGGGGGTATTCGTATTTCATTGTCAGAGGTGAAATTCTTGGATTTTTGAAAGACGAACTACTGCGAAAGCATTTACCAAGGATGTTTTCATTAATCAAGAACGAAAGTTAGGGGATCGAAGATGATTAGATACCATCGTAGTCTTAACCATAAACTATGCCGACAAGGGATTGGCGGAGTTTCGTTATGTCTCCGTCAGCACCTTATGAGAAATCACAAGTCTTTGGGTTCCGGGGGGAGTATGGTCGCAAGGCTGAAACTTAAAGAAATTGACGGAAGGGCACCACCAGGAGTGGAGCCTGCGGCTTAATTTGACTCAACACGG

>19-110

AGCAGGCGCGCAAATTGCCCAATCCTGACACAGGGAGGTAGTGACAATAAATAACAATGCCGGGCCTTTGTAGGTCTGGCAATTGGAATGAGAACAATTTAAACCCCTTATCGAGTATCAATTGGAGGGCAAGTCTGGTGCCAGCAGCCGCGGTAATTCCAGCTCCAATAGCGTATATTAAAGTTGTTGCAGTTAAAAAGCTCGTAGTTGGATTTGTGGCTGTCACGGGCGGCCTATCACTTAGTGTTAGTGCTTGCCTGTGTCGCCATCCTTGGGTGGAGCCCGTGTGGCATTAGGTTGTCGTGCGGGGGATGCCCATCGTTTACTGTGAGAAAATTAGAGTGTTCAAAGCAGGCTTATGCCGTTGAATATATTAGCATGGAATAATAAGATAGGACCTTGGTACTATTTTGTTGGTTTGCGCACCAAGGTAATGATTAATAGGGACAGTTGGGGGTATTCGTATTCCATTGTCAGAGGTGAAATTCTTGGATTTTTGGAAGACGAACTACTGCGAAAGCATTTACCAAGGATGTTTTCATTAATCAAGAACGAAAGTTAGGGGATCGAAGATGATTAGATACCATCGTAGTCTTAACCATAAACTATGCCGACAAGGGATTGGCGGAGTCTCGTTTTGTCTCCGTCAGCACCTTATGAGAAATCACAAGTCTTTGGGTTCCGGGGGGAGTATGGTCGCAAGGCTGAAACTTAAAGAAATTGACGGAAGGGCACCACCAGGAGTGGAGCCTGCGGCTTAATTTGACTCAACACGG

>19-112

AGCAGGCGCGTAAATTGCCCAATCCCGACACGGGGAGGTAGTGACAATAAATAACAATACCGGGCATTTAATGTCTGGTAATTGGAATGAGTACAATCTAAATCCCTTAACGAGGATCAATTGGAGGGCAAGTCTGGTGCCAGCAGCCGCGGTAATTCCAGCTCCAATAGCGTATATTTAAGTTGTTGCAGTTAAAAAGCTCGTAGTTGGATTTCGGGTGGGTGACAGCGGTCCGGCTCTGCTGTGTACTGCTGTTGCCTTCCTTTCTGTCGGGGACGGGCTCTTGGACTTCACTGTCCGGGACTCGGAGTCGGCGAGGTTACTTTGAGTAAATTAGAGTGTTCAAAGCAAGCGTTCGCTATGAATACATTAGCATGGAATAACATGATAGGACTCTGGCTTATCTTGTTGGTCTGTAAGACCGGAGTAATGATTAAGAGGGACAGTCGGGGGCATTCGTATTTCATTGTCAGAGGTGAAATTCTTGGATTTATGAAAGACGAACTTCTGCGAAAGCATTTGCCAAGGATGTTTTCATTAATCAAGAACGAAAGTTGGGGGCTCGAAGACGATTAGATACCGTCGTAGTCTCAACCATAAACGATGCCGACTAGGGATTGGCAGGTGTTTCGTTGATGACCCTGCCAGCACCTTATGAGAAATCAAAGTTTTTGGGTTCCGGGGGGAGTATGGTCGCAAGGCTGAAACTTAAAGGAATTGACGGAAGGGCACCACCAGGCGTGGAGCCTGCGGCTTAATTTGACTCAACACGG

>19-116

AGCAGGCGCGCAAATTGCCCAATCCTGACACAGGGAGGTAGTGACAATAAATAACAATGCCGGGCCATCATTGGTCTGGCAATTGGAATGAGAACAATTTAAATCCCTTATCGAGGATCAATTGGAGGGCAAGTCTGGTGCCAGCAGCCGCGGTAATTCCAGCTCCAATAGCGTATATTAAAGTTGTTGCAGTTAAAAAGCTCGTAGTTGGATTTGTGGCGTACGGTGTGTTCCGGGCACTTGTTGTCTGAGTAACCCGCTGTTGCCATCCTTGGGTGGAACCTGTGTGGCATTAGGTTGTCGTGCAGGGGATGCCCATCATTTACTGTGAAAAAATTAGAGTGTTCAAAGCAGGCTTATGCCGTTGAATATATTAGCATGGAATAATGAGATAGGACTTTTTTGCTATTTTGTTGGTTTGCGCAAGAAGGTAATGATTAATAGGGACAGTTGGGGGTATTCGTATTCCATTGTCAGAGGTGAAATTCTTGGATTTTTGGAAGACGAACTACTGCGAAAGCATTTACCAAGGATGTTTTCATTAATCAAGAACGAAAGTTAGGGGATCGAAGATGATTAGATACCATCGTAGTCTTAACCATAAACTATGCCGACAAGGGATTGGTGGAGTCTCGTTATGTCTCCATCAGCACCTTATGAGAAATCACAAGTCTTTGGGTTCCGGGGGGAGTATGGTCGCAAGGCTGAAACTTAAAGAAATTGACGGAAGGGCACCACCAGGAGTGGAGCCTGCGGCTTAATTTGACTCAACACGG

>19-118

AGCAGGCGCGTAAATTGCCCAATCCTAAAGTAGGGAGGTAGTGACAATAAATACTGACGCTGGATTCAATGAATCTGGTAAAGAATGAGAACAGTCTAAAACCCTTATCGAGGACCCATTGGAGGGCAAGTCTGGTGCCAGCAGCCGCGGTAATTCCAGCTCCAATAGCGTATATTAAAGTTGTTGCAGTTAAAAAGCTCGTAGTTGAATTTCGGGTCCCCGCGCTCGGCGCTTCATTGTGCCGTCGTGGTGTCCCTCCTCGCCGTCAGGCGTCCTTTACTGTGAAAAAATTAGAGTGTTCAAAGCAGGCAATCGCTTGAATACATTAGCATGGAATAATAAGATAGGATCTTGTCTATTTTGTTGGTTGCGGACAATGATAATGATTAATAGGGATAGTTGGGGGTGTCCGTATTGAGCTGTCAGAGGTGAAATTCTTGGATTTGCTCAAGACGAACTACTGCGAAAGCATTCACCAAGGATGTTTTCATTAATCAAGAACGAAAGTCAGGGGATCGAAGAGGATTAGATACCCTCGTAGTCTTGACCATAAACTATGCCGACTCGGGATTGGCAGACGTTGTATCGACTCTGTCAGCACCGTATGAAAAATCAAAGTCTTTGGGTTCCGGGGGGAGTATGGTCGCAAGGCTGAAACTTAAAGGAATTGACGGAAGGGCACCACCAGGAGTGGAGCCTGCGGCTTAATTTGACTCAACACGG

>19-120

AGCAGGCGCGTAAATTGCCCAATCTTGACACAGGGAGGTAGTGACAATAAATAACAATGCCGGGCCTTTCTAGGTCTGGCAATTGGAATGAGAACAATTTAAATCCCTTATCGAGGATCAATTGGAGGGCAAGTCTGGTGCCAGCAGCCGCGGTAATTCCAGCTCCAATAGCGTATATTAAAGTTGTTGCAGTTAAAAAGCTCGTAGTTGGATTTCTGGTGGGAGCGATCGGTCTGGCGCTTAGCGCGAGTACTTGCATCGTCTCCGGCCATCCTTGGGGAGAGTCTGTCTGGCATTAGGTTGTCGGGCAGAGGATCCTCATCGTTTACTGTGAAAAAATTAGAGTGTTCAAAGCAGGCTTATGCCGTTGAATATATTAGCATGGAATAATAAGATAGGACCTTGGTACTATTTTGTTGGTTTGCGCACCAAGGTAATGATTAATAGGGACAGTTGGGGGTATTCGTATTCCATTGTCAGAGGTGAAATTCTTGGATTTTTGGAAGACGAGCTACTGCGAAAGCATTTACCAAGGATGTTTTCATTAATCAAGAACGAAAGTTAGGGGATCGAAGATGATTAGATACCATCGTAGTCTTAACCATAAACTATGCCGACAAGGGATTGGCGGAGTTTCGTTATGTCTCCGTCAGCACCTTATGAGAAATCACAAGTCTTTGGGTTCCGGGGGGAGCATGGTCGCAAGGCTGAAACTTAAAGAAATTGACGGAAGGGCACCACCAGGAGTGGAGCCTGCGGCTTAATTTGACTCAACACGG

>19-124

AGCAGGCGCGTAAATTGCCCAATCCTAGCTCGGGGAGGTAGTGACGAAAAATAACAATACGGGACTCTTACGAGGCCCCGTAATTGGAATGAGTACACTTTAAATCCTTTGTCGAGGATCTATTGGAGGGCAAGTCTGGTGCCAGCAGCCGCGGTAATTCCAGCTCCAATAGCGTATATTAAAGTTGCTGCAGTTAAAAAGCTCGTAGTTGGATTTGTGGCGCGGCCGTCCGGCGGGGCTCCTTCACTGGGGCCGTTTGTCTGGTTTTTCGGCCGCTCCATTCTCGGGTAGTGTGTCGCTGGCATTAGGTTGTCGGCTTCTTCACGCCCGTCGTTTACTGTGAAAAAATTAGAGTGTTCAAAGCAGGCTTAGGCCATTGGATACATTAGCATGGAATAATGAGATAGGGCCACGGCGGTCTATTTTGTTGGTTTGCACGTTGTGGTAATGATTAACAGGAACGGTTGGGGGTATTCGTATTCAATTGTCAGAGGTGAAATTCTTGGATTTATGGAAGACGAACTACTGCGAAAGCATTTACCAAGGATGTTTTCATTAATCAAGAACGAAAGTTAGGGGATCGAAGATGATTAGATACCATCGTAGTCTTAACCATAAACTATGCCGACTAGGGATTGGCGGTCGTTAATTTCAGGACTCCGTCAGCACCTTCCGAGAAATCAGAGTCTTTGGGTTCCGGGGGGAGTATGGTCGCAAGGCTGAAACTTAAAGAAATTGACGGAAGGGCACCACCAGGAGTGGAGCCTGCGGCTTAATTTGACTCAACACGG

>19-127

AGCAGGCGCGTAAATTGCCCAATCCTGACACAGGGAGGTAGTGACAATAAATAACAATGCCGGGCCTTTACAGGTCTGGCAATTGGAATGAGAACAATTTAAATCCCTTATCGAGTATCAATTGGAGGGCAAGTCTGGTGCCAGCAGCCGCGGTAATTCCAGCTCCAATAGCGTATATTAAAGTTGTTGCAGTTAAAAAGCTCGTAGTTGGATTTGTGGTGTACTTCGGCGGCCCGTCACAATGTGATGGAGTCTGCTGAAGTCGCCATCCTTGGGTGGATCCTGTGTGGCATTAAGTTGTCGTGCAGGGGATGCCCATCGTTTACTGTGAAAAAATTAGAGTGTTCAAAGCAGGCTTATGCCGTTGAATATATTAGCATGGAATAATAAGATAGGACCTTGGTACTATTTTGTTGGTTTGCGCACCAAGGTAATGATTAATAGGGACAGTTGGGGGTATTCGTATTTCATTGTCAGAGGTGAAATTCTTGGATTTTTGAAAGACGAACTACTGCGAAAGCATTTACCAAGGATGTTTTCATTAATCAAGAACGAAAGTTAGGGGATCGAAGATGATTAGATACCATCGTAGTCTTAACCATAAACTATGCCGACAAGGGATTGGTGGAGTTTCGTTATGTCTCCATCAGCACCTTATGAGAAATCACAAGTCTTTGGGTTCCGGGGGGAGTATGGTCGCAAGGCTGAAACTTAAAGAAATTGACGGAAGGGCACCACCAGGAGTGGAGCCTGCGGCTTAATTTGACTCAACACGG

>19-130

AGCAGGCGCGAAAATTGCCCAATCCCGGCACGGGGAGGTAGTGACGAAAAATAACAATACGGGTCTTTTAATAGGCCCCGTAATTGGAATGAGTACACCTTAAAACCATTAACGAGTACCAATTGGAGGGCAAGTCTGGTGCCAGCAGCCGCGGTAATTCCAGCTCCAATAGCGTATATTAAAGTTGTTGCAGTTAAAAAGCTCGTAGTTGGATCTGAGGATTAGAGCCACAGGTTGGCCGCATGGCTACACTTGTGGCTCAGCCTGCATAGTCGGTGATTGCTTTGGTGCTCTTAATTGAGTGCCTTAACTGCCCGGCCACGTTTACTTTGAAAAAATTGGAGTGCTCAAATCAGGCCCACTGCCTGAACAGATGTGCATGGAATAATGGAAAAGGACTTCGGTTCTATTTTGTTGGTTTTCGGAACATGAAGTAATGATTAAGAGGGACAGACGGGGGCATTCGTATTGCGGTGGGAGAGGTGAAATTCTTGGATCATCGCAAGACGCCCTACAGCGAAAGCATTTGCCAAGAATGTTTCCATTAATCAAGAACGAAAGTCAGAGGTTCGAAGACGATCAGATACCGTCCTAGTTCTGACCATAAACGATGCCAACTGGCAATCCGTTGCGATTGCAAGTTCGATCCAACGGGCAGCCTCCGGGAAACCAAAGTCTTTGGGTTCCGGGGGGAGTATGGTCGCAAGGCTGAAACTTAAAGGAATTGACGGAAGGGCACCACCAGGAGTGGAGCCTGCGGCTTAATTTGACTCAACACGG

>19-135

AGCAGGCGCGTAAATTGCCCAATCCTGACACAGGGAGGTAGTGACAATAAATAACAATGCCGGGCCTTTGTAGGTCTGGCAATTGGAATGAGAACAATTTAAACCCCTTATCGAGTATCAATTGGAGGGCAAGTCTGGTGCCAGCAGCCGCGGTAATTCCAGCTCCAATAGCGTATATTAAAGTTGTTGCAGTTAAAAAGCTCGTAGTTGGATTTGTGGTGTACTTCGGCGGCCCGTCACTATGTGATGGAGCTTGCTGAAGTCGCCATCCTTGGGTGGATCCTGTGTGGCATTAAGTTGTCGTGCAGGGGATGCCCATCGTTTACTGTGAAAAAATTAGAGTGTTCAAAGCAGGCTTATGCCGTTGAATATATTAGCATGGAATAATAAGATAGGACCTTGGTACTATTTTGTTGGTTTGCGCACCAAGGTAATGATTAATAGGGACAGTTGGGGGTATTCGTATTTCATTGTCAGAGGTGAAATTCTTGGATTTTTGAAAGACGAACTACTGCGAAAGCATTTACCAAGGATGTTTTCATTAATCAAGAACGAAAGTTAGGGGATCGAAGATGATTAGATACCATCGTAGTCTTAACCATAAACTATGCCGACAAGGGATTGGTGGAGTTTCGTTATGTCTCCATCAGCACCTTATGAGAAATCACAAGTCTTTGGGTTCCGGGGGGAGTATGGTCGCAAGGCTGAAACTTAAAGAAATTGACGGAAGGGCACCACCAGGAGTGGAGCCTGCGGCTTAATTTGACTCAACACGG

>19-145

AGCAGGCGCGCAAATTGCCCAATCCTGACACAGGGAGGTAGTGACAATAAATAACAATGCCGGGCCTTTACAGGTCTGGCAATTGGAATGAGAACAATTTAAATCCCTTATCGAGTATCAATTGGAGGGCAAGTCTGGTGCCAGCAGCCGCGGTAATTCCAGCTCCAATAGCGTATATTAAAGTTGTTGCAGTTAAAAAGCTCGTAGTTGGATTTCTGGCAGGAGGGACCGGTCACGCACTCTGTGTGTGAACTTGGCTCCCTCTGGCCATCCTTGGGGAGATCCTGTTTGGCATTAAGTTGTCGGGCAGGGGATACCCATCGTTTACTGTGAAAAAATTAGAGTGTTTAAAGCAGGCTTATGCCGTTGAATATATTAGCATGGAATAATAAGATAGGACTTCGGAACTATTTTGTTGGTTTGCGTTACGAAGTAATGATTAATAGGGACAGTTGGGGGTATTCGTATTTCGTTGTCAGAGGTGAAATTCTTGGATTTCCGAAAGACGAACTACTGCGAAAGCATTTACCAAGGATGTTTTCATTAATCAAGAACGAAAGTTAGGGGATCGAAGATGATTAGATACCATCGTAGTCTTAACCATAAACTATGCCGACTCAGGATTGGCGGTTGTTTTTTGACTCCGTCAGCACTGTATGAGAAATCAAAGTCTTTGGGTTCCGGGGGGAGTATGGTCGCAAGGCTGAAACTTAAAGAAATTGACGGAAGGGCACCACCAGGAGTGGAACCTGCGGCTTAATTTGACTCAACACGG

>19-147

AGCAGGCGCGTAAATTGCCCAATCTCGGTACGGGGAGGTAGTGACGAAAAATAACAATACGGGTCTTTTTATAGGCCCCGTAATTGGAATGAGTACACTTTAAAAGCATTAACGAGTACCAATTGGAGGGCAAGTCTGGTGCCAGCAGCCGCGGTAATTCCAGCTCCAATAGCGTATATTAAAGTTGTTGCAGTTAAAAAGCTCGTAGTTGGATCTGAGGAGTGGAGCTTACGGTTGGTCGCATGACTACACTGTTGGCTCGGCCTACATAGTCGGTGATTGTTTTGGTGCTCTTAATTGAGTGCCTTAATTACCCGGCCACGTTTACTTTGAAAAAATTGGAGTGCTCAAATCAGGCCTACTGCCTGAACAGATGTGCATGGAATAATGGAAAAGGACTTCGGTTCTATTTTGTTGGTTTTCGGAACATGAAGTAATGATTAAGAGGGACAGACGGGGGCATTCGTATTGCGGTGGGAGAGGTGAAATTCTTAGATCATCGCAAGACGCCCAACAGCGAAAGCATTTGCCAAGAATGTTTCCATTAATCAAGAACGAAAGTCAGAGGTTCGAAGACGATCAGATACCGTCCTAGTTCTGACCATAAACGATGCCAACTGGCAATCCGTTGCGATTGCAAGTTCGATCCAACGGGCAGCCTCCGGGAAACCAAAGTCTTTGGGTTCCGGGGGAAGTATGGTTGCAAAGCTGAAACTTAAAGGAATTGACGGAAAGGCACCACAAGGAGTGGAGCCTGCGGCTTAATTTGACTCAACACGG

>19-150

AGCAGGCGCGAAAATTGCCCAATCCTGACACAGGGAGGTAGTGACAATAAATAACAATGCCGGGCCTTTGTTGGTCTGGCAATTGGAATGAGAACAATTTAAACCCCTTATCGAGGATCAATTGGAGGGCAAGTCTGGTGCCAGCAGCCGCGGTAATTCCAGCTCCAATAGCGTATATTAAAGTTGTTGCAGTTAAAAAGCTCGTAGTTGGATTTGTGGTGTACTTCGTGTTCCCGGCACTTGGTGACGGAGCTTGCCGATGTCGCCATCCTTGGGTGGATCCTGTGTGGCATTAAGTTGTCGTGCAGGGGATGCCCATCATTTACTGTGAAAAAATTAGAGTGTTCAAAGCAGGCTTATGCCGTTGAATATATTAGCATGGAATAATGAGATAGGACTTTTTTGCTATTTTGTTGGTTTGCGCAAAAAGGTAATGATTAATAGGGACAGTTGGGGGTATTCGTATTCCATTGTCAGAGGTGAAATTCTTGGATTTTTGGAAGACGAACTACTGCGAAAGCATTTACCAAGGATGTTTTCATTAATCAAGAACGAAAGTTAGGGGATCGAAGATGATTAGATACCATCGTAGTCTTAACCATAAACTATGCCGACAAGGGATTGGTGGAGTCTCGTTATGTCTCCATCAGCACCTTATGAGAAATCACAAGTCTCTGGGTTCCGGGGGGAGTATGGTCGCAAGGCTGAAACTTAAAGAAATTGACGGAAGGGCACCACCAGGAGTGGAGCCTGCGGCTTAATTTGACTCAACACGG

>19-7

AGCAGGCGCGTAAATTGCCCAATCTTGACACAGGGAGGTAGTGACAATAAATAACAATGCCGGGCCTTTATAGGTCTGGCAATTGGAATGAGAACAATTTAAACCCCTTATCGAGTATCAATTGGAGGGCAAGTCTGGTGCCAGCAGCCGCGGTAATTCCAGCTCCAATAGCGTATATTAAAGTTGTTGCAGTTAAAAAGCTCGTAGTTGGATTTGTGGCTGTCTCGAGCGGCCTGACACCTAGTGTTAGTGCTTGCTTGAGTCGCCATCCTTGGGTGGAACCTGTGTGGCATTAGGTTGTCGTGCAGGGGATGCCCATCGTTTACTGTGAAAAAATTAGAGTGTTCAAAGCAGGCTTATGCCGTTGAATATATTAGCATGGAATAATAAGATAGGACCTTGGTACTATTTTGTTGGTTTGCGCACCAAGGTAATGATTAATAGGGACAGTTGGGGGTATTCGTATTCCATTGTCAGAGGTGAAATTCTTGGATTTCTGGAAGACGAACTACTGCGAAAGCATTTACCAAGGATGTTTTCATTAATCAAGAACGAAAGTTAGGGGATCGAAGATGATTAGATACCATCGTAGTCTTAACCATAAACTATGCCGACAAGGGATTGGCGGAGTTTCGTTTTGTCTCCGTCAGCACCTTATGAGAAATCACAAGTCTTTGGGTTCCGGGGGGAGTATGGTCGCAAGGCTGAAACTTAAAGAAATTGACGGAAGGGCACCACCAGGAGTGGAGCCTGCGGCTTAATTTGACTCAACACGG

>19-11

AGCAGGCGCGTAAATTGCCCAATCCTGACACAGGGAGGTAGTGACAATAAATAACAATGCCGGGCCTTTGTAGGTCTGGCAATTGGAATGAGAACAATTTAAACCCCTTATCGAGTATCAATTGGAGGGCAAGTCTGGTGCCAGCAGCCGCGGTAATTCCAGCTCCAATAGCGTATATTAAAGTTGTTGCAGTTAAAAAGCTCGTAGTTGGATTTGTGGTGTACTTCGGCGGCCCGTCACAATGTGATGGAGCTTGCTGAAGTCGCCATCCTTGGGTGGATCCTGTGTGGCATTGAGTTGTCGTGCAGGGGATGCCCATTGTTTACTGTGAGAAAATTAGAGTGTTCAAAGCAGGCTTAT

GCCGTTGAATATGTTAGCATGGAATAATAAGATAGGACCTTGGTACTATTTTGTTGGTTTGCGCACCAAGGTAATGATTAATAGGGACAGTTGGGGGTATTCGTATTTCATTGTCAGAGGTGAAATTCTTGGATTTTTGAAAGACGAACTACTGCGAAAGCATTTACCAAGGATGTTTTCATTAATCAAGAACGAAAGTTAGGGGATCGAAGATGATTAGATACCATCGTAGTCTTAACCATAAACTATGCCGACAAGGGATTGGCGGAGTTTCGTTATGTCTCCGTCAGCACCTTATGAGAAATCACAAGTCTTTGGGTTCCGGGGGGAGTATGGTCGCAAGGCTGAAACTTAAAGAAATTGACGGAAGGGCACCACCAGGAGTGGAGCCTGCGGCTTAATTTGACTCAACACGG

>19-36

AGCAGGCGCGCAAATTGCCCAATCTTGACACAGGGAGGTAGTGACAATAAATAACAATGCCGGGCTCTTCGAGTCTGGCAATTGGAATGAGAACAATCTAAAACCCTTATCGAGGATCAATTGGAGGGCAAGTCTGGTGCCAGCAGCCGCGGTAATTCCAGCTCCAATAGCGTATATTAAAGTTGTTGCAGTTAAAAAGCTCGTAGTTGGATTTCTCGCACGAGGGCTTGGCCGCGGCCTCACGACCGTCGCGCCGTGGCTTCTTGTGCGTTCCTCGGCCGGGTCCGTGGTGGCATTAGGTTGTCGTCGCGGGGAAGGTCGTCGTTTACTGTGAAAAAATTAGAGTGTTCAAAGCGAGGCTTATGCCATTGGATACATTAGCATGGAATAATAAGATAGGACCTCGGTGTTTTTATTTTGTTGGTTTGCACGCCAAGGTAATAGTTAATAGGGATAGTTGGGGGTATTCGTATTCAATTGTCAGAGGTGAAATTCTTGGATTTATGGAAGACGAACTACTGCGAAAGCATTTACCAAGGATGTTTTCATTAATCAAGAACGAAAGTTGGGGGATCGAAGATGATTAGATACCATCGTAGTCCCAACCATAAACTATACCGACTCGGGATCGGCGGTCGTCAATTCACGACCCCGTCGGCACCGTTCGAGAAATCAAAGTCTTTGGGTTCCGGGGGGAGTATGGTCGCAAGGCTGAAACTTAaAGAAATTGACGGAAGGGCACCACCAGGAGTGGAGCCTGCGGCTTAATTTGACTCAACACGG

>19-46

AGCAGGCGCGAAAATTGCCCAATCCTGACACAGGGAGGTAGTGACAATAAATAACAATGCCGGGCCTTTGTAGGTCTGGCAATTGGAATGAGAACAATTTAAACCCCTTATCGAGTATCAATTGGAGGGCAAGTCTGGTGCCAGCAGCCGCGGTAATTCCAGCTCCAATAGCGTATATTAAAGTTGTTGCAGTTAAAAAGCTCGTAGTTGGATTTGTGGCGTTCGCGTGCGTCCCGGCATTCGTGCCGGTGTTTGCTAGCGTCGCCATCCTTGGGTGGAACTGGTGTGGCATTAAGTTGTCGTGTCAGGGATGCCCATCATTTACTGTGAAAAAATTAGAGTGTTCAAAGCAGGCTTACGCCACTGAATATATTAGCATGGAATAATGAGATAGGACCTTGGTACTATTTTGTTGGTTTGCGCACCGAGGTAATGATTAATAGGGACAGTTGGGGGTATTCGTATTCCATTGTCAGAGGTGAAATTCTTGGATTTCTGGAAGACGAACTACTGCGAAAGCATTTACCAAGGATGTTTTCATTAATCAAGAACGAAAGTTAGGGGATCGAAGATGATTAGATACCATCGTAGTCTTAACCATAAACTATGCCGACAAGGGATTGGCGGAGTTTCGTTTTGTCTCCGTCAGCACCTTATGAGAAATCACAAGTCTTTGGGTTCCGGGGGGAGTATGGTCGCAAGGCTGAAACTTAAAGAAATTGACGGAAGGGCACCACCAGGAGTGGAGCCTGCGGCTTAATTTGACTCAACACGG

>19-61

AGCAGGCGCGTAAATTGCCCAATCTCGACTCGGGGAGGTAGTGACAATAAATAACAATGCCGGTCCCTATAGTGGTCCGGCAATTGGAATGAGTACAATCCAAACCCCTTAACGAGGAACAATTGGAGGGCAAGTCTGGTGCCAGCAGCCGCGGTAATTCCAGCTCCAATAGCGTATATTAAAGTTGTTGCAGTTAAAAAGCTCGTAGTTGGATTTCGGGGCCCCGGACGCTCGTTGCATGTGTCACTGGACGAGTTCAGTGGCCGTGTGGCGGTCGTCGGTCTCTGAGTCGGATCGGTCCGCCGAGAGGCGTGTACTGACCGACGGCCCCTGTTGTCTCTCGGGTTGCCCGCCTGCACTTAACTGTTGTGGACGGGGAGTTCGGGACGTTTACTTTGAACAAATTAGAGTGTTCCAAGCAGGCCGTTTGCTTTGAATACATTAGCATGGAATAATGGAATAGGGCTGCGTGGCCTGTCGTTTCGGCGGCGGGTCCTCGTGTCCTATTTTGTTGGTTTTCGGGACCGCAGTCATGGTCAAGAGGGACAGTTGGGGGCATTCGTATTCAGTTGTCAGAGGTGAAATTCTTGGATTTACGGAAGACGAACAACTGCGAAAGCATTTGCCAAGGATGTTTTCATTAATCAAGAACGAAAGTTGGGGGTTCGAAGACGATCAGATACCGTCGTAGTCCCAACCATAAACCATGCCAGCTAGGGATCAGCGGACGTCGGTAGCGACTCCGTTGGCACCTCTCGAGAAATCCAAGCTTTTGGGTTCCGGGGGGAGTATGGTCGCAAGGCTGAAACTTAAAGGAATTGACGGAAGGGCACCACCAGGAGTGGAGCCTGCGGCTTAATTTGACTCAACACGG

>19-68

AGCAGGCGCGTAAATTGCCCAATCTCAATTCAGGGAGGTAGTGACAAGAAATAACAATGCTGGGGCAATTCGCTTCTGCAATTGGAATGAGAACAACTTAAACCACTTAACGAGTACCAATTGGAGGGCAAGTCTGGTGCCAGCAGCCGCGGTAATTCCAGCTCCAATAGCGTATATGTAAGTTGTTGCGATTAAAAAGCCCGTAGCTGAACCTCGGACTTCCCTGCCGGTCCGCATTATGTGGGTACTGTCGTGGGTTGTCTTTCTGATAAGTCCGTATCCAGGCGTTTATTCGCCTGAAACGTGGTTTATCAAGGTTACTTTGAGTAAAATAGAGTGTTCAAAGCAAGCACGCGCTGTGAATACACTAGCATGGGATAACACGACATGACCGATGGTCTATCTTGTTGGCCTGTAGATCTGTTGGTAATGGCTAATAGGGACGGTCGGGGGCATTCGTATTTCGCTGTCAGAGGTGAAATTCTTAGATTTGCGAAAGACGAACTACTGCGAAAGCATTTGCCAAGGACGCTTTCATTAGTCAAGGACGAAAGTCGGGGGATCGAAGACGATTAGATACCGTCGTAGTCTCGACCATAAACGATGCCAATTAGGGATTGGAGGGTGGATTCATTATGCCTCCTCCAGCACCTTCCGCGAAAGCAAAATCTATGGGCTTCGGGGGGAGTATGGTCGCAAGGCTGAAACTTAAAGGAATTGACGGAACGGCACCACCAGGCGTGGAGCCTGCGGCTTAATTTGACTCAACACGG

>19-73

AGCAGGCGCGCAAATTGCCCAATCTCGGCACGGGGAGGTAGTGACGAAAAATAACAATACGGGTCTTTTAATAGGCCCCGTAATTGGAATGAGTACACCTTAAAACCATTAACGAGTACCAATTGGAGGGCAAGTCTGGTGCCAGCAGCCGCGGTAATTCCAGCTCCAATAGCGTATATTAAAGTTGTTGCAGTTAAAAAGCTCGTAGTTGGATTTGTGGCTGTCACAAGCGGCCTATCACCTAGTGTTAGTGCTTGCTGGTGTCGCCATCCTTGGGTGGAGCCCGTGTGGCATTAGGTTGTCGTGCGGGGGATGCCCATCGTTTACTGTGAGAAAATTAGAGTGTTCAAAGCAGGCTTATGCCGTTGAATATATTAGCATGGAATAATGAGATAGGACCTTGGTGCTATTTTGTTGGTTTGCGCGCCGTGGTAATGATTAACAGGAACGGTTGGGGGTCTTCGTATTCCATTGTCAGAGGTGAAATTCTTGGATTTCTGGAAGACGAACTACTGCGAAAGCATTGACCAAGGATGTTTTCATTAATCAAGAACGAAAGTTAGGGGATCGAAGATGATTAGATACCATCGTAGTCTTAACCATAAACTATGCCGACAAGGGATTGGTGGGGTTCCGTAATGTCCCCATCAGCACCTTCTGAGAAATCACAAGTTTTTGGGTTCCGGGGGGAGTATGGTCGCAAGGCTGAAACTTAaAGAAATTGACGGAAGGGCACCACCAGGAGTGGAGCCTGCGGCTTAATTTGACTCAACACGG

>19-78

AGCAGGCGCGTAAATTGCCCAATCTTGACACGGGGAGGTAGTGACGAAAAATACTGATGCGGGACTCAGTCGAGGCTCCGTAATCGGAATGAGTACACTTTAAATCCTTTAACGAGGATCTATTAGAGGGCAAGTCTGGTGCCAGCAGCCGCGGTAATTCCAGCTCTAATAGCGTATATTAAAGTTGTTGCAGTTAAAAAGCTCGTAGTTGGATCTCGGGCCGCCTGCGCGTGGTCTCGCGGGTTATCTAAGGCTATAGTCCGCCCGTGTAGCACTGCCGTGGGTGCCTATCTGGTGGGGGGAGGTTCCTCGGTTCACTCCTCGGCGCGGCCTCGTGTCGTCCGGGGAGGGGGGAGCGCTCCTGCGAGAGCAGTCGCCAGTACCCTTAGCTGGGTCGCTGGAGGCACCCGCCTCGTTTACTTTGAAAAAATTAGAGTGTTCAAAGCAGGCTGAAGCTGGCCTGAATAATGGCGCATGGAATAATAGAATATGACCTCGGGTCTATTTTGTTGGTCTCCGGACCTGGAGGTAATGATTAAGAGGGACAGACGGGGGCATTCGTACTGCGGCGTTAGAGGTGAAATTCTTGGATCGCCGCAAGACGAACTACTGCGAAAGCATTTGCCAAGAATGTTTTCATTAATCAAGAACGAAAGTCGGAGGTTCGAAGACGATCAGATACCGTCCTAGTTTCGACCATAAACGATGCCGACTAGCGATCTGCCAGAGTTGTTAACCATAGGACCTGGCGGGCAGCTCCCGGGAAACCAAAGTCTTTGGGTTCTGGGGGAAGTATGGTTGCAAAGCTGAAACTTAAAGGAATTGACGGGAGGGCACCACCAGGAGTGGAGCCTGCGGCTTAATTTGACTCAACACGG

>19-80

AGCAGGCGCGTAAATTGCCCAATCTTGACACAGGGAGGTAGTGACAATAAATATCAATTCTGGGCCACATGGTCCGGTAATTGGAATGAGTACAATGTAAACGCCTTAACGAGGATCCATTGGAGGGCAAGTCTGGTGCCAGCAGCCGCGGTAATTCCAGCTCCAATAGCGTATATTTAAGTTGTTGCAGTTAAAAAGCTCGTAGTTGGATTTCGGGTGGGACGCAGCGGTCTCGCATTGCGTTTGTACTGCTGCAGCCCTCCTTCTTGCCGGGGGCGGCCTCTCGGACTTCACTGTCTGGGGGTCAGAATCGGCGATGTTACTTTGAGTAAATTAGAGTGTTCAAAGCAAGCTTACGCTCTGAATATAATAGCATGGGATAACACGACAGGACTCTGGCCTATCGTGTTGGTCTATAGGACCAGAGTAATGATTAAGAGGGACAGTCGGGGGCATTCGTATTCCGTTGTCAGAGGTGAAATTCTTGGATTTACGGAAGACGAACATCTGCGAAAGCATTTGCCAAGGATGTTTTCATTGATCAAGAACGAAAGTTGGGGGCTCGAAGACGATTAGATACCGTCGTAGTCTCAACCATAAACGATGCCGACTAGGGATTGGCGGGTGTTTTTTTGATGACTCCGCCAGCACCTCATGAGAAATCAAAGTTTTTGGGTTCCGGGGGGAGTATGGTCGCAAGGCTGAAACTTAAAGGAATTGACGGAAGGGCACCACCAGGCGTGGAGCCTGCGGCTTAATTTGACTCAACACGG

>19-91

AGCAGGCGCGTAAATTGCCCAATCCCGGCACGGGGAGGTAGTGACGAAAAATAACAAGCTGGTCTCAATGAGTTCCGGCATTGTAATGGGAACAATTTAAATCATTTATCGAGGATCTATTGGAGGGCAAGTCTGGTGCCAGCAGCCGCGGTAATTCCAGCTCCAATAGCGTATATTAAAGTTGTTGCGGTTAAAAAGCTCGTAGTTGGATCTAGGATTAGGGCTACGGTCGTTCAGCTGGACGCACTGTATGCCCGACTCCTTTTCTCTAGTATTCGATTGTTGCTCTTAAATGAGTGACTTTAGTTGCTGGAACTTTTACCTTGAAAAAATTGAAGTGCTCAAAGCAGGTTTTGCCTGAACATTGCTGCATGGGATAATAGAATAGGATTTTGGTCCTATTTCTGTTGGTTTCGGGACACGAAATAATGATTAATAGGGACAGACGGGGGCATTCGTATTGCTGCGTTAGAGGTGAAATTCTTGGATCGCAGCAAGACGAACTACTGCGAAAGCATTTGCCAAGAATGTTTTCATTAATCAAGAACGAAAGTTAGAGGTTCGAAGGCGATCAGATACCGCCCTAGTTCTAACCGTAAACGATGCTAACTTGCGATCCGCAGGAGTTACTTCAACGGCTCTGCGGGAAGCACCCGGGAAACCGAAGTGTTCGAGTTCCGGGGGGAGTATGTTTGCAAAGATGACACTTAAAGGAATTGACGGAAGGGCACCACCATGGAGTGGAGCTTGCGGCTTAATTTGACTCAACACGG

>19-97

AGCAGGCGCGTAAATTGCCCAATCTCGGCACGGGGAGGTAGTGACGAAAAATAACAATACGGGTCTTTTAATAGGCCCCGTAATTGGAATGAGTACACCTTAAAACCATTAACGAGTACCAATTGGAGGGCAAGTCTGGTGCCAGCAGCCGCGGTAATTCCAGCTCCAATAGCGTATATTAAAGTTGTTGCAGTTAAAAAGCTCGTAGTTGGATCTGAGGATTAGAGCCACAGGTTGGCCGCATGGCTACACTTGTGGCTCAGCCTGCATAGTCGGTGATTGCTTTGGTGCTCTTAATTGAGTGCCTTAACTGCCCGGCCACGTTTACTTTGAAAAAATTGGAGTGCTCAAATCAGGCCCACTGCCTGAACAGATGTGCATGGAATAATGGAAAAGGACTTCGGTTCTATTTTGTTGGTTTCTAGAGCTGAGGTAATGATTAATAGGGATGGTTGGGGGCATTCGTATTTAATTGTCAGAGGTGAAATTCTTGGATTTGTTAAAGACGGACGACTGCGATAGCATTTGCCAAGGATGTTTTCATTGATCAAGAACGAAAGTTAGGGGATCGAAGACGATCAGATACCGTCGTAGTCTTAACCATAAACCATGCCGACTAGAGATTGGAGGTCGTTTTCCACACGACCCCTTCAGCACCTTATGAGAAATCAAAGTCTTTGGGTTCCGGGGGGAGTATGGTCGCAAGGCTGAAACTTAAAGGAATTGACGGAAGGGCACCACCAGGAGTGGAGCCTGCGGCTTAATTTGACTCAACACGG

>19-106

AGCAGGCGCGTAAATTGCCCAATCCTGACACAGGGAGGTAGTGACAATAAATAACAATGCCGGGCCTTCTTAGGTCTGGCAATTGGAATGAGAACAATTTAAACCCCTTATCGAGTATCAATTGGAGGGCAAGTCTGGTGCCAGCAGCCGCGGTAATTCCAGCTCCAATAGCGTATATTAAAGTTGTTGCAGTTAAAAAGCTCGTAGTTGGATTTGTGGCGTACGGTGTGTTCCGGGCACTTGTTGTCTGAGTAACCTGCTGTTGCCATCCTTGGGTGGAATCTGTGTGGCATTAAGTTGTCGTGCAGGGGATGCCCATCGTTTACTGTGAAAAAATTAGAGTGTTCAAAGCAGGCTTATGCCGTTGAATATATTAGCATGGAATAATAAGATAGGACCTTGGTACTATTTTGTTGGTTTGCGCACCAAGGTAATGATTAATAGGGACAGTTGGGGGTATTCGTATTTCATTGTCAGAGGTGAAATTCTTGGATTTTTGAAAGACGAACTACTGCGAAAGCATTTACCAAGGATGTTTTCATTAATCAAGAACGAAAGTTAGGGGATCGAAGATGATTAGATACCATCGTAGTCTTAACCATAAACTATGCCGACAAGGGATTGGTGGAGTTTCGTTATGTCTCCATCAGCACCTTATGAGAAATCACAAGTCTTTGGGTTCCGGGGGGAGTATGGTCGCAAGGCTGAAACTTAAAGAAATTGACGGAAGGGCACCACCAGGAGTGGAGCCTGCGGCTTAATTTGACTCAACACGG

>19-109

AGCAGGCGCGCAAATTGCCCAATCCTGACACAGGGAGGTAGTGACAAAAAATAACAATACCTGGCCTTTTTTAGGTCGGGTAATTGGAATGAGTACAATTTAAATCCCTTATCGAGGACCCATTGGAGGGCAAGTCTGGTGCCAGCAGCCGCGGTAATTCCAGCTCCAATAGCGTATATTAAAGTTGTTGCAGTTAAAAAGCTCGTAGTTGAATTTCGGATCGCCACTCCCGCCACTTCATTGTGGCGATGTGGAGTTCCTCCTCGCCTTTGGCGTCATTTACTGTGAAAAAATTAGAGTGTTCAAAGCAGGCAATCGCTTGAATACATTAGCATGGAATAATAAGATAGGATCTTCGTCTATTTTGTTGGTTGCGGACTGAGATAATGATTAATAGGGATAGTTGGGGGTGTCTGTATTGTGTTGTCAGAGGTGAAATTCTTAGATTTACACAAGACAAACTACTGCGAAAGCATTCACCAAGGATGTTTTCATTAATCAAGAACGAAAGTCAGGGGATCGAAGAGGATTAGATACCCTCGTAGTCTTGACCATAAACTATGCCGACTCGGGATTGGCAGACGATTGTTTCGACTCTGTCAGCACCGTATGAAAAATCAAAGTCTTTGGGTTCCGGGGGGAGTATGGTCGCAAGGCTGAAACTTAAAGGAATTGACGGAAGGGCACCACCAGGAGTGGAGCCTGCGGCTTAATTTGACTCAACACGG

>19-129

AGCAGGCGCGTAAATTGCCCAATCCTGACACAGGGAGGTAGTGACAATAAATAACAATGCCGGGCTTTTTCAAGTCTGGCAATTGGAATGAGAGCAATTTAAACCCATCATCGAGTATCAATTGGAGGGCAAGTCTGGTGCCAGCAGCCGCGGTAATTCCAGCTCCAATAGCGTATATTAAAGTTGTTGCAGTTAAAAAGCTCGTAGTTGGATTTGTGGCTGTCGCGGGCGGCCTATCACTTTGTGTTAGTGCTTGCCTGCGTCGCCATCCTTGGGTGGAGCCCGTGTGGCATTAGGTTGTCGTGCGGGGGATGCCCATCGTTTACTGTGAGAAAATTAGAGTGTTCAAAGCAGGCTTATGCCGTTGAATATGTTAGCATGGAATAATAAGATAGGACCTCGGTACTATTTTGTTGGTTTGCGCACCGAGGTAATGATTAATAGGGACAGTTGGGGGTATTCGTATTCCATTGTCAGAGGTGAAATTCTTGGATTTCTGGAAGACGAACTACTGCGAAAGCATTTACCAAGGATGTTTTCATTAATCAAGAACGAAAGTTAGGGGATCGAAGATGATTAGATACCATCGTAGTCTTAACCATAAACTATGCCGACAAGGGATTGGCGGAGTTTCGTTTTGTCTCCGTCAGCACCTTATGAGAAATCACAAGTCTTTGGGTTCCGGGGGGAGTATGGTCGCAAGGCTGAAACTTAAAGAAATTGACGGAAGGGCACCACCAGGAGTGGAGCCTGCGGCTTAATTTGACTCAACACGG

>19-136

AGCAGGCGCGAAAATTGCCCAATCTCGGTACGGGGAGGTAGTGACGAAAAATAACAATACGGGTCTTTTTATAGGCCCCGTAATTGGAATGAGTACACTTTAAAAGCATTAACGAGTACCAATTGGAGGGCAAGTCTGGTGCCAGCAGCCGCGGTAATTCCAGCTCCAATAGCGTATATTAAAGTTGTTGCAGTTAAAAAGCTCGTAGTTGGATCTGAGGAGTGGAGCTTACGGTTGGTCGCATGACTACACTGTTGGCTCGGCCTACATAGTCGGTGATTGTTTTGGTGCTCTTAATTGAGTGCCTTAATTACCCGGCCACGTTTACTTTGAAAAAATTGGAGTGCTCAAATCAGGCCTACTGCCTGAACAGATGTGCATGGAATAATGGAAAAGGACTTCGGTTCTATTTTGTTGGTTTTCGGAACATGAAGTAATGATTAAGAGGGACAGACGGGGGCATTCGTATTGCGGTGGGAGAGGTGAAATTCTTAGATCATCGCAAGACGCCCAACAGCGAAAGCATTTGCCAAGAATGTTTCCATTAATCAAGAACGAAAGTCAGAGGTTCGAAGACGATCAGATACCGTCCTAGTTCTGACCATAAACGATGCCAACTGGCAATCCGTTGCGATTGCATGTTCGATCCAACGGGCAGCCTCCGGGAAACCAAAGTCTGTGGGTTCCGGGGGAAGTATGGTTGCAAAGCTGAAACTTAAAGGAATTGACGGAAAGGCACCACAAGGAGTGGAGCCTGCGGCTTAATTTGACTCAACACGG

>19-140

GCTTGCATGCCTGCAGGTCGACGATTATCTCTAGAGGATCCCCGGGTACCGAGCTCGAATTCGTAATCATGGTCATAGCTGTTTCCTGTGTGAAATTGTTATCCGCTCACAATTCCACACAACATACGAGCCGGAAGCATAAAGTGTAAAGCCTGGGGTGCCTAATGAGTGAGCTAACTCACATTAATTGCGTTGCGCTCACTGCCCGCTTTCCAGTCGGGAAACCTGTCGTGCCAGCTGCATTAATGAATCGGCCAACGCGCGGGGAGAGGCGGTTTGCGTATTGGGCGCTCTTCCGCTTCCTCGCTCACTGACTCGCTGCGCTCGGTCGTTCGGCTGCGGCGAGCGGTATCAGCTCACTCAAAGGCGGTAATACGGTTATCCACAGAATCAGGGGATAACGCAGGAAAGAACATGTGAGCAAAAGGCCAGCAAAAGGCCAGGAACCGTAAAAAGGCCGCGTTGCTGGCGTTTTTCCATAGGCTCCGCCCCCCTGACGAGCATCACAAAAATCGACGCTCAAGTCAGAGGTGGCGAAACCCGACAGGACTATAAAGATACCAGGCGTTTCCCCCTGGAAGCTCCCTCGTGCGCTCTCCTGTTCCGACCCTGCCGCTTACCGGATACCTGTCCGCCTTTCTCCCTTCGGGAAGCGTGGCGCTTTCTCATAGCTCACGCTGTAGGTATCTCAGTTCGGTGTAGGTCGTTCGCTCCAAGCTGGGCTGTGTGCACGAACCCCCCGTTCAGCCCGACCGCTGCGCCTTATCCGGTAACTATCGTCTTGAGTCCAACCCGGTAAGACACGACTTATCGCCACTGGCAGCAGCCACTGGTAACAGGATTAGCAGAGCGAGGTATGTAGGCGGTGCTACAGAGTTCTTGAAGTGGTGGCCTAACTACGGCTACACTAGAAGAACAG

>19-146

AGCAGGCGCGAAAATTGCCCAATCCTGACACAGGGAGGTAGTGACAATAAATAACAATGCCGGGCCTTTAAAGGTCTGGCAATTGGAATGAGAACAATTTAAACCCCTTATCGAGGACCAATTGGAGGGCAAGTCTGGTGCCAGCAGCCGCGGTAATTCCAGCTCCAATAGCGTATATTAAAGTTGTTGCAGTTAAAAAGCTCGTAGTTGGATTTGTGGTTGAGCTGTTCGGTCCAACACTTTTGTGTTGGTACTTGGACGGCTCGTCCATCCTTGGGTGGAATCTGTGTGGCATTAGGTTGTCGTGCAGGGGATGCCCATCTTTTACTGTGAAAAAATTAGAGTGTTCAAAGCAGGCTTATGCCGTTGAATATATTAGCATGGAATAATGAGATAGGACTTTTTTGCTATTTTGTTGGTTTGCGCAAAGAGGTAATGATTAATAGGGACAGTTGGGGGTATTCGTATTCCATTGTCAGAGGTGAAATTCTTGGATTTTTGGAAGACGAACTACTGCGAAAGCATTTACCAAGGATGTTTTCATTAATCAAGAACGAAAGTTAGGGGATCGAAGATGATTAGATACCATCGTAGTCTTAACCATAAACTATGCCGACAAGGGATTGGCGGAGTTTCGTTTTGTCTCCGTCAGCACCTTATGAGAAATCACAAGTCTTTGGGTTCCGGGGGGAGTATGGTCGCAAGGCTGAACTTAAAGAAATTGACGGAAGGGCACCACCAGGAGTGGAGCCTGCGGCTTAATTTGACTCAACACGG

>24-1

AGCAGGCGCGCAAATTGCCCAATCTTGACACAGGGAGGTAGTGACAATAAATAACAATGCCGGGCCTTTGTAGGTCTGGCAATTGGAATGAGAACAATTTAAACCCCTTATCGAGTATCAATTGGAGGGCAAGTCTGGTGCCAGCAGCCGCGGTAATTCCAGCTCCAATAGCGTATATTAAAGTTGTTGCAGTTAAAAAGCTCGTAGTTGGATTTGTGGCTGTCGCGGGCGGCCTATCACTTTGTGTTAGTGCTTGCCTGCGTCGCCATCCTTGGGTGGAACCCGTGTGGCATTAGGTTGTCGTGCGGGGGATGCCCATCGTTTACTGTGAGAAAATTAGAGTGTTCAAAGCAGGCTTATGCCGTTGAATATGTTAGCATGGAATAATAAGATAGGACCTCGGTACTATTTTGTTGGTTTGCGCACCGAGGTAATGATTAATAGGGACAGTTGGGGGTATTCGTATTCCATTGTCAGAGGTGAAATTCTTGGATTTCTGGAAGACGAACTACTGCGAAAGCATTTACCAAGGATGTTTTCATTAATCAAGAACGAAAGTTAGGGGATCGAAGATGATTAGATACCATCGTAGTCTTAACCATAAACTATGCCGACAAGGGATTGGCGGAGTTTCGTTTTGTCTCCGTCAGCACCTTATGAGAAATCACAAGTCTTTGGGTTCCGGGGGGAGTATGGTCGCAAGGCTGAAACTTAAAGGAATTGACGGAAGGGCACCACCAGGAGTGGAGCCTGCGGCTTAATTTGACTCAACACGG

>24-4

AGCTTGCATGCCTGCAGGTCGACGATTCCGTGTTGAGTCAAATTAAGCCGCAGGTTCCACTCCTGGTGGTGCCCTTCCGTCAATTTCTTTAAGTTTCAGCCTTGCGACCATACTCCCCCCGGAACCCAAAGACTTTGATTTCTCATACAGTGCTGACGGAGTCAAAAAACAACCGCCAATCCTGAGTCGGCATAGTTTATGGTTAAGACTACGATGGTATCTAATCATCTTCGATCCCCTAACTTTCGTTCTTGATTAATGAAAACATCCTTGGTAAATGCTTTCGCAGTAGTTCGTCTTTCGGAAATCCAAGAATTTCACCTCTGACAACGAAATACGAATACCCCCAACTGTCCCTATTAATCATTACTTCGTAACGCAAACCAACAAAATAGTTCCGAAGTCCTATCTTATTATTCCATGCTAATATATTCAACGGCATAAGCCTGCTTTAAACACTCTAATTTTTTCACAGTAAACGATGGGTATCCCCTGCCCGACAACTTAATGCCAAACAGGATCTCCCCAAGGATGGCGACTTCAGCAGACTCCATCACATTGTGACGGGCCGCCGAAGTACACCACAAATCCAACTACGAGCTTTTTAACTGCAACAACTTTAATATACGCTATTGGAGCTGGAATTACCGCGGCTGCTGGCACCAGACTTGCCCTCCAATTGATACTCGATAAGGGGTTTAAATTGTTCTCATTCCAATTGCCAGACCTACAAAGGCCCGGCATTGTTATTTATTGTCACTACCTCCCCGAGTCGAGATTGGGCAATTTGCGCGCCTGCTAATCTCTAGAGGATCCCCGGGTACCGAGCTCGAATTCGTAATCATGGTCATAGCTGTTTCCTGTGTGAAATTGTTATCCGCTCACAATTCCACACAACATACGAGCCGGAAGCATAAAGTGTAAAGCCTGGGGTGCCTAATGAGT

>24-6

AAGCTTGCATGCcTGCAGGTCGACGATTCCGTGTTGAGTCAAATTAAGCCGCAGGCTCCACTCCTGGTGGTGCCCTTCCGTCAATTTCTTTAAGTTTCAGCCTTGCGACCATACTCCCCCCGGAACCCAAAGACTTGTGATTTCTCATAAGGTGCTGATGGAGACATAACGAGACTCCACCAATCCCTTGTCGGCATAGTTTATGGTTAAGACTACGATGGTATCTAATCATCTTCGATCCCCTAACTTTCGTTCTTGATTAATGAAAACATCCTTGGTAAATGCTTTCGCAGTAGTTCGTCTTCCAAAAATCCAAGAATTTCACCTCTGACAATGGAATACGAATACCCCCAACTGTCCCTATTAATCATTACCTCTTTGCGCAAACCAACAAAATAGCAAAAAAGTCCTATCTCATTATTCCATGCTAATATATTCAACGGCATAAGCCTGCTTTGAACACTCTAATTTTTTCACAGTAAATGATGGGCATCCCCTGCACGACAACCTAATGCCACACAGGTTCCACCCAAGGATGGCAACAGCAGGTTACTCAGACAACAAGTGCCCGGAACACACCGTACGCCACAAATCCAACTACGAGCTTTTTAACTGCAACAACTTTAATATACGCTATTGGAGCTGGAATTACCGCGGCTGCTGGCACCAGACTTGCCCTCCAATTGATCCTCGATAAGGGATTTAAATTGTTCTCATTCCAATTGCCAGACCAATGATGGCCCGGCATTGTTATTTATTGTCACTACCTCCCTGTGTCAGGATTGGGCAATTTACGCGCCTGCTAATCTCTAGAGGATCCCCGGGTACCGAGCTCGAATTCGTAATCATGGTCATAGCTGTTTCCTGTGTGAAATTGTTATCCGCTCACAATTCCACACAACATACGAGCCGGAAGCATAAAGTGTAAAGCCTGGGGTGCCTA

>24-7

AGCAGGCGCGTAAATTGCCCAATCCTAATGCAGGGAGGTAGTGACAATAAATACTGACGCTGGACTCTACGAGTCTGGTAAAGAATGAGAACAGTTTAAAACCCTTATCGAGGACCCATTGGAGGGCAAGTCTGGTGCCAGCAGCCGCGGTAATTCCAGCTCCAAAAGCGTATACTGAAATTGTTGCGGTTAAAAAGCTCGTAGTTGGATCTTCGACCATCAAGAGCGGTAGCCCTTTGGGTTTAACTGCTTCTTGTGGTCTCCTTGCTGGTCTTTCCTTGGTGCTCTTAATTGAGTGCCTTGGTTGGCTAGTGCTTTTACTTTGAAAAAATTAGAGTGCTCAAAGCAGGCGTTTGCCTGAATAGTGGTGCATGGAATAATGGAAGAGGACCTCGGTCCTATTTTGTTGGTTTCTAGGGCCGAAGTAATGATTAAGAGGGACAGTTGGGGGCATTCGTATTTAATTGTCAGAGGTGAAATTCTTGGATTTATGAAAGACGAACTAGTGCGAAAGCATTTGCCAAGGATGTTTTCATTAATCAAGAACGAAAGTTAGGGGTTCGAAGACGATCAGATACCGTCGTAGTCCTAACCATAAACTATGCCGACTAGGGATCGGTGGATGTTAATGTTTGACTCCATCGGCACCTTATGAGAAATCAAGGTCTTTGGGTTCCGGGGGGAGTATGGTCGCAAGGCTGAAACTTAAAGGAATTGACGGAAGGGCACCACCAGGAGTGGAGCCTGCGGCTTAATTTGACTCAACACGG

>24-13

AGCAGGCGCGTAAATTGCCCAATCCTGACACAGGGAGGTAGTGACAATAAATAACAATGCCGGGCCTTTAAAGGTCTGGCAATTGGAATGAGAACAATTTAAACCCCTTATCGAGGATCAATTGGAGGGCAAGTCTGGTGCCAGCAGCCGCGGTAATTCCAGCTCCAATAGCGTATATTAAAGTTGTTGCAGTTAAAAAGCTCGTAGTTGGATTTGTGGTTGAGGCGGCCGGCCCTCACTTTGTGTGGTGTTGAGCTGCCTCGTCCATCCTTGGGTGGAACCAGTGTGGCATTAAGTTGTCGTGTTGGGGATGCCCATCATTTACTGTGAAAAAATTAGAGTGTTCAAAGCAGGCTTACGCCGTTGAATATATTAGCATGGAATAATGAGATAGGACCTTGGTACTATTTTGTTGGTTTGCGCACCGAGGTAATGATTAATAGGGACAGTTGGGGGTATTCGTATTCCATTGTCAGAGGTGAAATTCTTGGATTTCTGGAAGACGAACTACTGCGAAAGCATTTACCAAGGATGTTTTCATTAATCAAGAACGAAAGTTAGGGGATCGAAGATGATTAGATACCATCGTAGTCTTAACCATAAACTATGCCGACAAGGGATTGGCGGGCGTTGTATTGACTCCGTCAGCACCTTATGAGAAATCACAAGTCTTTGGGTTCCGGGGGGAGTATGGTCGCAAGGCTGAAACTTAAAGAAATTGACGGAAGGGCACCACCAGGAGTGGAGCCTGCGGCTTAATTTGACTCAACACGG

>24-14

AGCAGGCGCGTAAATTGCCCAATCCTGACACAGGGAGGTAGTGACAATAAATAACAATGCCGGGCCTTTGTAGGTCTGGCAATTGGAATGAGAACAATTTAAACCCCTTATCGAGTATCAATTGGAGGGCAAGTCTGGTGCCAGCAGCCGCGGTAATCCCAGCTCCAATAGCGTATATTAAAGTTGTTGCAGTTAAAAAGCTCGTAGTTGGATTTGTGGCGTACGGTGTGTTCCGGGCACTTGTTGTCTGAGTAACCTGCTGTTGCCATCCTTGGGTGGAACCTGTGTGGCATTAGGTTGTCGTGCAGGGGATGCCCATCATTTACTGTGAAAAAATTAGAGTGTTCAAAGCAGGCTTATGCCGTTGAATATATTAGCATGGAATAATAAGATAGGACCTTGGTACTATTTTGTTGGTTTGCGCACCAAGGTAATGATTAATAGGGACAGTTGGGGGTATTCGTATTTCATTGTCAGAGGTGAAATTCTTGGATTTTTGAAAGACGAACTACTGCGAAAGCATTTACCAAGGATGTTTTCATTAATCAAGAACGAAAGTTAGGGGATCGAAGATGATTAGATACCATCGTAGTCTTAACCATAAACTATGCCGACAAGGGATTGGCGGAGTTTCGTTATGTCTCCGTCAGCACCTTATGAGAaATCACAAGTCTTTGGGTTCCGGGGGGAGTATGGTCGCAAGGCTGAAACTTAAAGAAATTGACGGAAGGGCACCACCAGGAGTGGAGCCTGCGGCTTAATTTGACTCAACACGG

>24-18

AGCTTGCATGCCTGCAGGTCGACGATTCCGTGTTGAGTCAAATTAAGCCGCAGGCTCCACTCCTGGTGGTGCCCTTCCGTCAATTTCTTTAAGTTTCAGCCTTGCGACCATACTCCCCCCGGAACCCAAAGACTTGTGATTTCTCATAAGGTGCTGACGGAGACATAACGAAACTCCGCCAATCCCTTGTCGGCATAGTTTATGGTTAAGACTACGATGGTATCTAATCATCTTCGATCCCCTAACTTTCGTTCTTGATTAATGAAAACATCCTTGGTAAATGCTTTCGCAGTAGTTCGTCTTTCAAAAATCCAAGAATTTCACCTCTGACAATGAAATACGAATACCCCCAACTGTCCCTATTAATCACTACCTTGGTGCGCAAACCAACAAAATAGTACCAAGGTCCTATCTTATTATTCCATGCTAATATATTCAACGGCATAAGCCTGCTTTGAACACTCTAATTTTTTCACAGTAAACGATGGGCATCCCCTGCACGACAACTTAATGCCACACAGGATCCACCCAAGGATGGCGACTTCAGCAAGCTCCAGCACTAAGTGATGGGCCGCCGAAGTACACCACAAATCCAACTACGAGCTTTTTAACTGCAACAACTTTAATATACGCTATTGGAGCTGGAATTACCGCGGCTGCTGGCACCAGACTTGCCCTCCAATTGATACTCGATAAGGGGTTTAAATTGTTCTCATTCCAATTGCCAGACCTACAAAGGCCCGGCATTGTTATTTATTGTCACTACCTCCCTGTGTCAGGATTGGGCAATTTACGCGCCTGCTAATCTCTAGAGGATCCCCGGGTACCGAGCTCGAATTCGTAATCATGGTCATAGCTGTTTCCTGTGTGAAATTGTTATCCGCTCACAATTCCACACAACATACGAGCCGGAAGCATAAGTGTAAAGCCTGGGTGCCTAATG

>24-31

AGCAGGCGCGTAAATTGCCCGATCCTGACATAGGGAGGTAGTGACAAGAAATAACAATACAGGGCATCCATGTCTTGTAATTGGAATGAATGTGATTTAAACCCCTTTGTGAGTATCAATTGGAGGGCAAGTCTGGTGCCAGCAGCCGCGGTAATTCCAGCTCCAATAGCGTATATTAAAGTTGTTGCGGTTAAAAAGCTCGTAGTTGGATTTCTGCCGAGGACGACCGGTCCGCCTTTCGGGTGAGCATCTGGCTCGGCTTGGGCATATTCTTGGAGAACGTTCCTGCACTTGACTGTGCGGTGCGGTAGCCAAGAGCTTTACTTTGAGGAAATTAGAGTGTTCTAAGCAGGCAGATGCCTTGAATACATTAGCATGGAATAATGATGTAGGACTTCGGTTCTATTTTGTTGGTTTCTAGAGCTGAAGTAATGATTAATAGGGATGGTTGGGGGCATTCGTATTTAATTGTCAGAAGTGAAATTCTTGGATTTGTTAAAGACGGACGACTGCGATAGCATTTGCCAAGGATGTTTTCATTGATCAAGAACGAAAGTTAGGGGATCGAAGACGATCAGATACCGTCGTAGTCTTAACCATAAACCATGCCGACTAGAGATTGGAGGTCGTTTTCCACACGACCCCTTCAGCACCTTATGAGAAATCAAAGTCTTTGGGTTCCGGGGGGAGTATGGTCGCAAGGCTGAAACTTAAAGGAATTGACGGAAGGGCACCACCAGGAGTGGAGCCTGCGGCTTAATTTGACTCAACACGG

>24-32

AGCAGGCGCGTAAATTGCCCAATCTTGACACAGGGAGGTAGTGACAATAAATATCAATTCTGGGCCACATGGTCCGGTAATTGGAATGAGTACAATGTAAACGCCTTAACGAGGATCCATTGGAGGGCAAGTCTGGTGCCAGCAGCCGCGGTAATTCCAGCTCCAATAGCGTATATTTAAGTTGTTGCAGTTAAAAAGCTCGTAGTTGGATTTCGGGTGGGACGCAGCGGTCTCGCATTGCGTTTGTACTGCTGCAGCCCTCCTTCTTGCCGGGGGCGGCCTCTCGGACTTCACTGTCTGGGGGTCAGAATCGGCGATGTTACTTTGAGTAAATTAGAGTGTTCAAAGCAAGCTTACGCTCTGAATATAATAGCATGGGATAACACGACAGGACTCTGGCCTATCGTGTTGGTCTATAGGACCAGAGTAATGATTAAGAGGGACAGTCGGGGGCATTCGTATTCCGTTGTCAGAGGTGAAATTCTTGGATTTACGGAAGACGAACATCTGCGAAAGCATTTGCCAAGGATGTTTTCATTGATCAAGAACGAAAGTTGGGGGCTCGAAGACGATTAGATACCGTCGTAGTCTCAACCATAAACGATGCCGACTAGGGATTGGCGGGTGTTTTTTTGATGACTCCGCCAGCACCTCATGAGAAATCAAAGTTTTTGGGTTCCGGGGGGAGTATGGTCGCAAGGCTGAAACTTAAAGGAATTGACGGAAGGGCACCACCAGGCGTGGAGCCTGCGGCTTAATTTGACTCAACACGG

>24-33

AGCAGGCGCGTAAATTGCCCAATCCTGACACAGGGAGGTAGTGACAATAAATAACAATGCCGGGCCTTTCTAGGTCTGGCAATTGGAATGAGAACAATTTAAACCCCTTATCGAGTATCAATTGGAGGGCAAGTCTGGTGCCAGCAGCCGCGGTAATTCCAGCTCCAATAGCGTATATTAAAGTTGTTGCAGTTAAAAAGCTCGTAGTTGGATTTGTGGCTGGTTGTGTCGGCCTGGCATATTTGTGCTAGTGTTTGCCGCGACCGCCATCCTTGGGTGGATCCTGTGTGGCATTAGGTTGTCGTGCAGGGGATGCCCATCGTTTACTGTGAAAAAATTAGAGTGTTCAAAGCAGGCTTATGCCGTTGAATATATTAGCATGGAATAATAAGATAGGACCTTGGTACTATTTTGTTGGTTTGCGCACCAAGGTAATGATTAATAGGGACAGTTGGGGGTATTCGTATTCCATTGTCAGAGGTGAAATTCTTGGATTTTTGGAAGACGAACTACTGCGAAAGCATTTACCAAGGATGTTTTCATTAATCAAGAACGAAAGTTAGGGGATCGAAGATGATTAGATACCATCGTAGTCTTAACCATAAACTATGCCGACAAGGGATTGGCGGAGTCTCGTTTTGTCTCCGTCAGCACCTTATGAGAAATCACAAGTCTTTGGGTTCCGGGGGGAGTATGGTCGCAGGGCTGAAACTTAaAGAAATTGACGGAAGGGCACCACCAGGAGTGGAGCCTGCGGCTTAATTTGACTCAACACGG

>24-34

AGCTTGCATGCCTGCAGGTCGACGATTCCGTGTTGAGTCAAATTAAGCCGCAGGCTCCACTCCTGGTGGTGCCCTTCCGTCAATTCCTTTAAGTTTCAGCCTTGCGACCATACTCCCCCCGGAACCCAAAGACTTTGATTTCTCATAAGGTGCCGATGGAGTCAAACATTAACATCCACCGATCCCTAGTCGGCATAGTTTATGGTTAGGACTACGACGGTATCTGATCGTCTTCGAACCCCTAACTTTCGTTCTTGATTAATGAAAACATCCTTGGCAAATGCTTTCGCACTAGTTCGTCTTTCATAAATCCAAGAATTTCACCTCTGACAATTAAATACGAATGCCCCCAACTGTCCCTCTTAATCATTACTTCGGCCCTAGAAACCAACAAAATAGGACCGAGGTCCTCTTCCATTATTCCATGCTAATGTATTCAGGCGAGAGCCTGCCTTGAACACTCTAATTTTTTCAAAGTAAACGTCCCGAACTACCCGACCACTGCAATGAAGAGCAGTCGGGGCTTTCGAGAGGAAGGGCGGCTGACCAGTACTCACCTTGCGGTGGACCAGGCAGGCCACCCCGAAATCCAACTACGAGCTTTTTAACTGCAACAACTTTAATATACGCTATTGGAGCTGGAATTACCGCGGCTGCTGGCACCAGACTTGCCCTCCAATTGATACTCGATAAGGGGTTTAAATTGTTCTCATTCCAATTGCCAGACCTACAAAGGCCCGGCATTGTTATTTATTGTCACTACCTCCCTGTGTCAGGATTGGGCAATTTACGCGCCTGCTAATCTCTAGAGGATCCCCGGGTACCGAGCTCGAATTCGTAATCATGGTCATAGCTGTTTCCTGTGTGAAATTGTTATCCGCTCACAATTCCACACAACATACGAGCCGGAAGCATAAAGTGTAAAGCCTGGGGTGCCTAATGAGTG>24-37

AGCAGGCGCGTAAATTGCCCAATCCTGACACAGGGAGGTAGTGACAAGAAATAGCAATACCAGGCTTTATTAGGTCTGGCAATTGGAATGAGTACAATTTAAATCCCTTAACGAGGATCCACTGGAGGGCAAGTCTGGTGCCAGCAGCCGCGGTAATTCCAGCTCCAATAGCGTATATTAAAGTTGTTGCAGTTAAAAAGCTCGTAGTTGGATTTGTGGCGTACGGTGTGTTCCGGGCACTTGTTGTCTGAGTAACCTGCTGTTGCCATCCTTGGGTGGAACCTGTGTGGCATTAGGTTGTCGTGCAGGGGATGCCCATCATTTACTGTGAAAAAATTAGAGTGTTCAAAGCAGGCTTATGCCGTTGAATATATTAGCATGGAATAATGAGATAGGACTTTTTTGCTATTTTGTTGGTTTGCGCACCAAAGTAATGATTAATAGGGACAGTTGGGGGTATTCGTATTCCGTTGTCAGAGGTGAAATTCTTGGATTTACGGAAGACGAACTACTGCGAAAGCATTTACCAAGGATGTTTTCATTAATCAAGAACGAAAGTTAGGGGATCGAAGATGATCAGATACCATCGTAGTCTTAACCATAAACTATGCCGACTCGGGATTGGCGGGCGTTACTTTTTGACCCCGTCAGCACCGTATGTGAAAACAAAGTCTTTGGGTTCCGGGGGGAGTATGGTCGCAAGGCTGAAACTTAAAGAAATTGACGGAAGGGCACCACCAGGAGTGGAGCCTGCGGCTTAATTTGACTCAACACGG

>24-41

AGCAGGCGCGCAAATTGCCCAATCTTGACACAGGGAGGTAGTGACAATAAATAACAATGCCGGGCCCTTCTAGGTCTGGCAATTGGAATGAGAACAATTTAAATCCCTTATCGAGGATCAATTGGAGGGCAAGTCTGGTGCCAGCAGCCGCGGTAATTCCAGCTCCAATAGCGTATATTAAAGTTGTTGCAGTTAAAAAGCTCGTAGTTGGATTTGTGGCGCATGCGTCCGGCCGCCCCTTTTTGGGGTGTGCTGCGACGCGTCGCCATCTTTGGGTGGAATCTGCGTGGCATTAGGTTGTCGTGCAGGGGATGCCCATCGTTTACTGTGAAAAAATTAGAGTGTTCAAAGCAGGCTTATGCCGTTGAATATGTTAGCATGGAATAATGAGATAGGACCTTGGTACTATTTTGTTGGTTTGCGCACCAAGGTAATGGTTAATAGGGACAGTTGGGGGTATTCGTATTTCATTGTCAGAGGTGGAATTCTTGGATTTTTGAAAGACGAACTACTGCGAAAGCATTTACCAAGGATGTTTTCATTAATCAAGAACGAAAGTTAGGGGATCGAAGATGATTAGATACCATCGTAGTCTTAACCATAAACTATGCCGACAAGGGATTGGTGGGGTTTCATTTTGTCTCCATCAGCACCTTATGAGAAATCACAAGTCTTTGGGTTCCGGGGGGAGTATGGTCGCAAGGCTGAAACTTAAAGAAATTGACGGAAGGGCACCACCAGGAGTGGAGCCTGCGGCTTAATTTGACTCAACACGG

>24-42

AGCAGGCGCGTAAATTGCCCAATCTCAAATCGGGGAGGTAGTGACAAGAAATATCAATAGAGGACCCAATGGGTCTTCTAATTGGAATGAGAACAAGGTAAACATCTTATCGAGGATCCAGCAGAGGGCAAGTCTGGTGCCAGCAGCCGCGGTAATTCCAGCTCTGTAAGCGTATACCAAAGTTGTTGCAGTTAAAACGCTCGTAGTCGAACCTTGGCGGTTGTTTTGCGTTATCCTCACGGATGGCTGTGATTCGGCCGCCTTTGTGGAGGGGGGTGTAGTGGTTCTTTACTGAATTACTGCATTTCCGCCACCTTCTACTGTGAAAAAATTAGAGTGTTCAAAGCAGGCAATTGCCGTGAATACATTAGCATGGAATAATAGAATAAGACCTGTGTTCTATTTTGTTGGTTTGTTAGAAACGGGTAATGATTAAGAGGGATGGTCGGGGGCATTTGTATTCCAGCGCTAGAGGTGAAATTCTTGGATTGTTGGAAGACAAACTGCTGCGAAAGCGTCTGCCAAGGACATTTTCATTGATCAAGAACGAAAGTAAGGGGATCGAAGACGATCAGATACCGTCGTAGTCTTTACTATAAACGATGAGGACTGGGGATCGGGCGTTGCCTATTGGTACGCTCGGCACCCTTCGGGAAACCAAAGTGTTTGCTTTCTGGGGGGAGTATGGTCGCAAGGCTGAAACTTAAAGGAATTGACGGAAGGGCATCACCGGGTGTGGAGCCTGCGGCTTAATTTGACTCAACACGG

>24-44

AGCAGGCGCGTAAATTGCCCAATCTTGACACAGGGAGGTAGTGACAATAAATAACAATGCCGGGCCTTTGTAGGTCTGGCAATTGGAATGAGAACAATTTAAACCCCTTATCGAGTATCAATTGGAGGGCAAGTCTGGTGCCAGCAGCCGCGGTAATTCCAGCTCCAATAGCGTATATTAAAGTTGTTGCAGTTAAAAAGCTCGTAGTTGGATTTGTGGTGTACTTCGGCGGCCCGTCACTATGTGATGGAGCTTGCTGAAGTCGCCATCCTTGGGTGGATCCTGTGTGGCATTAAGTTGTCGTGCAGGGGATGCCCATCGTTTACTGTGAAAAAATTAGAGTGTTCAAAGCAGGCTTATGCCGTTGAATATATTAGCATGGAATAATGAGATAGGACTTTTTTGCTATTTTGTTGGTTTGCGCAAAGAGGTAATGATTAATAGGGACAGTTGGGGGCATTCGTATTCCATTGTCAGAGGTGAAATTCTTGGATTTTTGGAAGACGAACTACTGCGAAAGCATTTACCAAGGATGTTTTCATTAATCAAGAACGAAAGTTAGGGGATCGAAGATGATTAGATACCATCGTAGTCTTAACCATAAACTATGCCGACAAGGGATTGGTGGAGTCTCGTTATGTCTCCATCAGCACCTTATGAGAAATCACAAGTCTTTGGGTTCCGGGGGGAGTATGGTCGCAAGGCTGAAACTTAAAGAAATTGACGGAAGGGCACCACCAGGAGTGGAGCCTGCGGCTTAATTTGACTCAACACGG

>24-45

AGCAGGCGCGTAAATTGCCCAATCCTGACACAGGGAGGTAGTGACAATAAATAACAATGCCGGGCCTTTGTAGGTCTGGCAATTGGAATGAGAACAATTTAAACCCCTTATCGAGTATCAATTGGAGGGCAAGTCTGGTGCCAGCAGCCGCGGTAATTCCAGCTCCAATAGCGTATATTAATGTTGTTGCAGTTAAAAAGCTCGTAGTTGGATTTGTGGCTGTCACGGGCGGCCTATCACTTAGTGTTAGTGCTTGCCTGTGTCGCCATCCTTGGGTGGAGCCCGTGTGGCATTAGGTTGTCGTGCAGGGGATGCCCATCGTTTACTGTGAGAAAATTAGAGTGTTCAAAGCAGGCTTATGCCGTTGAATATGTTAGCATGGAATAATTAGATAGGACCTCGGTACTATTTTGTTGGTTTGCGCAAAGAGGTAATGATTAACAGGGACAGTTGGGGGTATTCGTATTCCATTGTCAGAGGTGAAATTCTTGGCTTTTTGGAAGACGAACTACTGCGAAAGCATTTACCAAGGATGTTTTCATTAATCAAGAACGAAAGTTAGGGGATCGAAGATGATTAGATACCATCGTAGTCTTAACCATAAACTATGCCGACAAGGGATTGGTGGAGTCTCGTTATGTCTCCATCAGCACCTTATGAGAAATCACAAGTCTTTGGGTTCCGGGGGGAGTATGGTCGCAAGGCTGAAACTTAAAGAAATTGACGGAAGGGCACCACCAGGAGTGGAGCCTGCGGCTTAATTTGACTCAACACGG

>24-48

AGCAGGCGCGTAAATTGCCCAATCTTGACACAGGGAGGTAGTGACAATAAATAACAATGCCGGGCCTTTGTAGGTCTGGCAATTGGAATGAGAACAATTTAAACCCCTTATCGAGTATCAATTGGAGGGCAAGTCTGGTGCCAGCAGCCGCGGTAATTCCAGCTCCAATAGCGTATATTAAAGTTGTTGCAGTTAAAAAGCTCGTAGTTGGATTTGTGGCTGTCTCCGGCGGCCCGTCACTTAGTGTCGGTGTTTGCTGGAGTCGCCATCCTTGGGTGGAGCCCGTGTGGCATTAGGTTGTCGTGCGGGGGATGCCCATCGTTTACTGTGAGAAAATTAGAGTGTTCAAAGCAGGCTTATGCCGTTGAATATGTTAGCATGGAATAATAAGATAGGACCTCGGTACTATTTTGTTGGTTTGCGCACCGAGGTAATGATTAATAGGGACAGTTGGGGGTATTCGTATTTCATTGTCAGAGGTGAAATTCTTGGATTTCTGAAAGACGAACTACTGCGAAAGCATTTACCAAGGATGTTTTCATTAATCAAGAACGAAAGTTAGGGGATCGAAGATGATTAGATACCATCGTAGTCTTAACCATAAACTATGCCGACTCGGGATCGGTGGTCTGTTTTTTGACTCCATCGGCACCGTATGAGAAATCAAAGTCTTTGGGTTCCGGGGGGAGTATGGTCGCAAGGCTGAAACTTAAAGAAATTGGCGGAAGGGCACCACCAGGAGTGGAGCCTGCGGCTTAATTTGACTCAACACGG

>24-49

AGCAGGCGCGCAAATTGCCCAATCTTGACACAGGGAGGTAGTGACAATAAATAACAATGCCGGGCCATCATTGGTCTGGCAATTGGAATGAGAACAATTTAAATCCCTTATCGAGGATCAATTGGAGGGCAAGTCTGGTGCCAGCAGCCGCGGTAATTCCAGCTCCAATAGCGTATATTAAAGTTGTTGCAGTTAAAAAGCTCGTAGTTGGATTTGTGGCGTACGGTGTGTTCCGGGCACTTGTTGTCTGAGTAACCTGCTGTTGCCATCCTTGGGTGGAACCTGTGTGGCATTAGGTTGTCGTGCAGGGGATGCCCATCATTTACTGTGAAAAAATTAGAGTGTTCAAAGCAGGCTTATGCCGTTGAATATATTAGCATGGAATAATGAGATAGGACTTTTTTGCTATTTTGTTGGTTTGCGCAAAGAGGTAATGATTAATAGGGACAGTTGGGGGTATTCGTATTCCATTGTCAGAGGTGAAATTCTTGGATTTTTGGAAGACGAACTACTGCGAAAGCATTTACCAAGGATGTTTTCATTAATCAAGAACGAAAGTTAGGGGATCGAAGATGATTAGATACCATCGTAGTCTTAACCATAAACTATGCCGACAAGGGATTGGTGGAGTCTCGTTATGTCTCCATCAGCACCTTATGAGAAATCACAAGTCTTTGGGTTCCGGGGGGAGTATGGTCGCAAGGCTGAAACTTAAAGAAATTGACGGAAGGGCACCACCAGGAGTGGAGCCTGCGGCTTAATTTGACTCAACACGG

>24-56

AGCAGGCGCGTAAATTGCCCAATCCTGACACAGGGAGGTAGTGACAATAAATAACAATGCCGGGCCTTTGTAGGTCAGGCAATTGGAATGAGAACAATTTAAACCCCTTATCGAGTATCAATTGGAGGGCAAGTCTGGTGCCAGCAGCCGCGGTAATTCCAGCTCCAATAGCGTATATTAATGTTGTTGCAGTTAAAAAGCTCGTAGTTGGATTTGTGGACTGGGCCGTCGGTCCGACACTTTTGTGTGGGTACCAGACGTTTCGGTTCATCCTTGGGTGGAATCTGTGTGGCATTAGGTTGTCGTGCAGGGGATGCCCATCATTTACTGTGAAAAAATTAGAGTGTTCAAAGCAGGCTTATGCCGTTGAATATATTAGCATGGAATAATGAGATAGGACTTTTTTGCTATTTTGTTGGTTTGCGCAAAGAGGTAATGATTAATAGGGACAGTTGGGGGTATTCGTATTCCATTGTCAGAGGTGAAATTCTTGGATTTTTGGAAGACGAACTACTGCGAAAGCATTTACCAAGGATGTTTTCATTAATCAAGAACGAAAGTTAGGGGATCGAAGATGATTAGATACCATCGTAGTCTTAACCATAAACTATGCCGACAAGGGATTGGTGGAGTCTCGTTATGTCTCCATCAGCACCTTATGAGAAATCACAAGTCTTTGGGTTCCGGGGGGAGTATGGTCGCAAGGCTGAAACTTAAAGAAATTGACGGAAGGGCACCACCAGGAGTGGAGCCTGCGGCTTAATTTGACTCAACACGG

>24-58

AGCAGGCGCGTAAATTGCCCAATCTCGACTCGGGGAGGTAGTGACAATAAATAACAATGCCGGGCTATTGTAGTCTGGCAATTGGAATGAGTACAATCTAAATCCCTTAACGAGGAACAATTGGAGGGCAAGTCTGGTGCCAGCAGCCGCGGTAATTCCAGCTCCAATAGCGTATATTAAAGTTGTTGCAGTTAAAAAGCTCGTAGTTGGATTTCGGGGTGGCCTGCCTGGTCCACCGCAAGGTGAGTACTGGTCAGCCGCCCTTCCTCTCGAAAGCCCCGACTGCTCTTCATTGCAGTGGTCGGGTAGTTCGGGACGTTTACTTTGAAAAAATTAGAGTGTTCAAGGCAGGCTCTCGCCTGAATACATTAGCATGGAATAATGGAAGAGGACCTCGGTCCTATTTTGTTGGTTTCTAGGGCCGAAGTAATGATTAAGAGGGACAGTTGGGGGCATTCGTATTTAATTGTCAGAGGTGAAATTCTTGGATTTATGAAAGACGAACTAGTGCGAAAGCATTTGCCAAGGATGTTTTCATTAATCAAGAACGAAAGTTAGGGGATCGAAGATGATTAGATACCATCGTAGTCTTAACCATAAACTATGCCGACAAGGGATTGGCGGAGTTTCGTTATGTCTCCGTCAGCACCTTATGAGAAATCACAAGTCTTTGGGTTCCGGGGGGAGTATGGTCGCAAGGCTGAAACTTAAAGAAATTGACGGAAGGGCACCACCAGGAGTGGAGCCTGCGGCTTAATTTGACTCAACACGG

>24-59

AGCAGGCGCGCAAATTGCCCAATCCTGACACAGGGAGGTAGTGACAAGAAATAACAATACAGGGCGTCAACGTCTTGTAATTGGAATGAATAGAATTTAAATCCCTTTATGAGTATCGATTGGAGGGCAAGTCTGGTGCCAGCAGCCGCGGTAATTCCAGCTCCAATAGCGTATATTAAAGTTGTTGCGGTTAAAAAGCTCGTAGTTGGATTTCTGCTGAGGACGACCGGTCCGCCCACTGGGTGAGTATCTGGCTCGGCCTTGGCATCTTCTTGGAGAACGTATCTGCACTTGACTGTGTGGTGCGGTAACCAGGACCTTTACTTTGAGGAAATTAGAGTGTTTCAAGCAGGCGTGCGCCTTGAATACATTAGCATGGAATAATAAGATAGGACCTTAGTTCTATTTTGTTGGTTTCTAGAGCTGGGGTAATGATTAATAGGGACAGTTGGGGGTATTCGTATTCCATTGTCAGAGGTGAAATTCTTGGATTTTTGGAAGACGAACTACTGCGAAAGCATTTACCAAGGATGTTTTCATTAATCAAGAACGAAAGTTAGGGGATCGAAGATGATTAGATACCATCGTAGTCTTAACCATAAACTATGCCGATAAGGGATTGGTGGAGTCTCGTTATGTCTCCATCAGCACCTTATGAGAAATCACAAGTCTTTGGGTTCCGGGGGGAGTATGGTCGCAAGGCTGAAACTTAAAGAAATTGACGGAAGGGCACCACCAGGAGTGGAGCCTGCGGCTTAATTTGACTCAACACGG

>24-61

AGCAGGCGCGTAAATTGCCCAATCTTAATTCAGGGAGGTAGTGACAATAAATAACAATACCGGGCTTCTTAAGTCTGGTAATTGGAATGAGTACAATCTAAATCCCTTAACGAGGATCCATTGGAGGGCAAGTCTGGTGCCAGCAGCCGCGGTAATTCCAGCTCCAATAGCGTATATTTAAGTTGTTGCAGTTAAAAAGCTCGTAGTTGGATTTCGGATGGGATCTGCCGGTCCGTCTTTGAGATGTGTACTGGTAGGTCCTGTCTTGTTGTCGGGGACCAGCTCCTGGGCTTCACTGTCCGGGACTGGGAGCCGACGAAGTTACTTTGAGTAAATTAGAGTGTTCAAAGCAAGCCTACGCTCTGAATATATTAGCATGGAATAACACGATAGGACTCTGGCTTATCTTGTTGGTCTGTGAGACCAGAGTAATGATTAAGAGGGACAGTCGGGGACATTCGTATTTCATTGTCAGAGGTGAAATTCTTGGATTTATGAAAGACGAACTTCTGCGAAAGCATTTGTCAAGGATGTTTTCATTAATCAAGAACGAAAGTTGGGGGCTCGAAGACGATTAGATACCGTCCTAGTCTCAACCATAAACGATGCCGACTAGGGATTGGCAGATGTTTTTTTGATGACTCTGCCAGCACCTTATGAGAAATCAAAGTTTTTGGGTTCCGGGGGGAGTATGGTCGCAAGGCTGAAACTTAAAGGAATTGACGGAAGGGCACCACCAGGCGTGGAGCCTGCGGCTTAATTTGACTCAACACGG

>24-64

AGCAGGCGCGTAAATTGCCCAATCCTGACACAGGGAGGTAGTGACAATAAATAACAATGCCGGGCCTTTGTAGGTCTGGCAATTGGAATGAGAACAATTTAAACCCCTTATCGAGTATCAATTGGAGGGCAAGTCTGGTGCCAGCAGCCGCGGTAATTCCAGCTCCAATAGCGTATATTAAAGTTGTTGCAGTTAAAAAGCTCGTAGTTGGATTTGTGGTGTACTTCGGCGGCCCGTCACTATGTGATGGAGCTTGCTGAAGTCGCCATCCTTGGGTGGATCCTGTGTGGCATTAAGTTGTCGTGTAGGGGATGCCCATCGTTTACTGTGAAAAAATTAGAGTGTTCAAAGCAGGCTTATGCCGTTGAATATATTAGCATGGAATAATAAGATAGGACCTTGGTACTATTTTGTTGGTTTACGCACCAAGGTAATGATTAATAGGGACAGTTGGGGGTATTCGTATTTCATTGTCAGAGGTGAAGTTCTTGGATTTTTGAAAGACGAACTACTGCGAAAGCATTTACCAAGGATGTTTTCATTAATCAAGAACGAAAGTTAGGGGATCGAAGATGATTAGATACCATCGTAGTCTTAACCATAAACTATGCCGACAAGGGATTGGTGGAGTTTCGTTATGTCTCCATCAGCACCTTATGAGAAATCACAAGTCTTTGGGTTCCGGGGGGAGTATGGTCGCAAGGCTGAAACTTAAAGAAATTGACGGAAGGGCACCACCAGGAGTGGAGCCTGCGGCTTAATTTGACTCAACACGG

>24-65

AGCTTGCATGCCTGCAGGTCGACGATTCCGTGTTGAGTCAAATTAAGCCGCAGGCTCCACGCCTGGTGGTGCCCTTCCGTCAATTCCTTTAAGTTTCAGCCTTGCGACCATACTCCCCCCGGAACCCAAAAACTTTGATTTCTCATAAGGTGCTGGCAGAGTCATCAAAAAAACATCTGCCAATCCCTAGTCGGCATCGTTTATGGTTGAGACTAGGACGGTATCTAATCGTCTTCGAGCCCCCAACTTTCGTTCTTGATTAATGAAAACATCCTTGACAAATGCTTTCGCAGAAGTTCGTCTTTCATAAATCCAAGAATTTCACCTCTGACAATGAAATACGAATGTCCCCGACTGTCCCTCTTAATCATTACTCTGGTCTCACAGACCAACAAGATAAGCCAGAGTCCTATCGTGTTATTCCATGCTAATATATTCAGAGCGTAGGCTTGCTTTGAACACTCTAATTTACTCAAAGTAACTTCGCCGGCTCCCAGTCCCGGACAGTGAAGCCCAGGAGCTGGTCCCCGACAACAAGATAGGACCTACCAGTACACACCTCAAAGGCGGACCGGCAGATCCCATCCGAAATCCAACTACGAGCTTTTTAACTGCAACAACTTAAATATACGCTATTGGAGCTGGAATTACCGCGGCTGCTGGCACCAGACTTGCCCTCCAATGGATCCTCGTTAAGGGATTTAGATTGTACTCATTCCAATTACCAGACTTAAGAAGCCCGGTATTGTTATTTATTGTCACTACCTCCCTGAATTAAGATTGGGCAATTTACGCGCCTGCTAATCTCTAGAGGATCCCCGGGTACCGAGCTCGAATTCGTAATCATGGTCATAGCTGTTTCCTGTGTGAAATTGTTATCCGCTCACAATTCCACACAACATACGAGCCGGAAGCATAAAGTGTAAAGCCTGGGGTGCCTAATGAG

>24-66

AGCAGGCGCGCAAATTGCCCAATCCTAATTCAGGGAGGTAGTGACAATAAATAACAATACCGGGCTTCTTAAGTCTGGTAATTGGAATGAGTACAATCTAAATCCCTTAACGAGGAACAATTGGAGGGCAAGTCTGGTGCCAGCAGCCGCGGTAATTCCAGCTCCAATAGCGTATATTAAAGTTGTTGCAGTTAAAAAGCTCGTAGTTGGATTTCGGATGGGATCTGCCGGTCCGCCTTTGAGGTGTGTACTGGTAGGTCCTATCTTGTTGTCGGGGACCAGCTCCTGGGCTTCACTGTCCGGGACTGGGAGCCGGCGAAGTTACTTTGAGTAAATTAGAGTGTTCAAAGCAAGCCTACGCTCTGAATATATTAGCATGGAATAACACGATAGGACTCTGGCTTATCTTGTTGGTCTGTGAGACCAGAGTAATGATTAAGAGGGACAGTCGGGGACATTCGTATTTCATTGTCAGAGGTGAAATTCTTGGATTTATGAAAGACGAACTTCTGCGAAAGCATTTGTCAAGGATGTTTTCATTAATCAAGAACGAAAGTTGGGGGCTCGAAGACGATTAGATACCGTCCTAGTCTCAACCATAAACGATGCCGACTAGGGATTGGCAGATGTTTTTTGATGACTCTGCCAGCACCTTATGAGAAATCAAAGTTTTTGGGTTCCGGGGGGAGTATGGTCGCAAGGCTGAAACTTAAAGGAATTGACGGAAGGGCACCACCAGGAGTGGAGCCTGCGGCTTAATTTGACTCAACACGG

>24-69

AGCTTGCATGCCTGCAGGTCGACGATTAGGCGCGTAAATTGCCCAATCTCAGTGCGAGGAGGTAGTGACGAAAAATAACGAGACAGTCTTCTATGAGGTCTGTCATCGGAATGGGTACAATTTAAACCCTTTAACGAGGATCTATTGGAGGGCAAGTCTGGTGCCAGCAGCCGCGGTAATTCCAGCTCCAAAAGCGTATACTAAAATTGTTGCGGTTAAAAAGCTCGTAGTTGGATCTTCGACCATCAAGAGCGGTAGCCCTTTGGGTTTAACTGCTTCTTGTGGTCTCCTTGCTGGTCTTTCCTTGGTGCTCTTAATTGAGTGCCTTGGTTGGCTAGTGCTTTTACTTTGAAAAAATTAGAGTGCTCAAAGCAGGCGTTTGCCTGAATAGTGGTGCATGGAATAATGGAATAGGACCTCGGTTCTATTTTGTTGGTTTTCAGAGCTCGAGGTAATGATTAAGAGGGACAAACGGGGGCATTCGTACTGCTACGTTAGAGGTGAAATCCTTGGATCGCAGCATGACGAACAACTGCGAAAGCATTTGCCAAGAATGTTTTCATTAATCAAGAACGATAGTTAGAGGTTCGAAGGCGATCAGATACCGCCCTAGTTCTAACCGTAAACGATGCCAACTAGCAATCCGCTGGTGTTAATTTTGACTCAGCGGGCGGCTGCCCGGAAACGATAAGTTTTTCGGTTCCGGGGGAAGTATGGTTGCAAATCCGAAACTTAAAGGAATTGACGGAAGGGCACCACCAGGAGTGGAGCCTGCGGCTTAATTTGACTCAACACGG

>24-83

AGCAGGCGCGAAAATTGCCCAATCTTAAAGTAGGGAGGTAGTGACAATAAATACTGACGCTGGATTCAATGAATCTGGTAAAGAATGAGAACAGTCTAAAACCCTTATCGAGGACCCATTGGAGGGCAAGTCTGGTGCCAGCAGCCGCGGTAATTCCAGCTCCAATAGCGTATATTAAAGTTGTTGCAGTTAAAAAGCTCGTAGTTGAGTTTCGGGTCCCCGCGCTCGGCGCTTCATTGTGCCGTCGTGGTGTCCCTCCTCGCCGTCAGGCGTCCTTTACTGTGAAAAAATTAGAGTGTTTAAAGCAGGCTTATGCCGTTGAATATATTAGCATGGAATAATAAGATAGGACTTCGGAACTATTTTGTTGGTTTGCGTTACGAAGTAATGATTAATAGGGACAGTTGGGGGTATTCGTATTTCGTTGTCAGAGGTGAAATTCTTGGATTTCCGAAAGACGAACTACTGCGAAAGCATTTACCAAGGATGTTTTCATTAATCAAGAACGAAAGTTAGGGGATCGAAGATGATTAGATACCATCGTAGTCTTAACCATAAACTATGCCGACAAGGGATTGGTGGAGTCTCGTTATGTCTCCATCAGCACCTTATGAGAAATCACAAGTCTTCGGGTTCCGGGGGGAGTATGGTCGCAAGGCTGACACTTAAAGAAATTGACGGAAGGGCACCACCAGGAGTGGAACCTGCGGCTTAATTTGACTCAACACGG

>24-85

AGCAGGCGCGAAAATTGCCCAATCCTAATTCAGGGAGGTAGTGACAATAAATAACAATACCGGGCTTCTTAAGTCTGGTAATTGGAATGAGTACAATCTAAATCCCTTAACGAGGATCCATTGGAGGGCAAGTCTGGTGCCAGCAGCCGCGGTAATTCCAGCTCCAATAGCGTATATTTAAGTTGTTGCAGTTAAAAAGCTCGTAGTTGGATTTCGGATGGGATCTGCCGGTCCGTCTTTGAGATGTGTACTGGTAGGTCCTATCTTGTTGTCGGGGACCAGCTCCTGGGCTTCACTGTCCGGGACTGGGAGCCGACGAAGTTACTTTGAGTAAATTAGAGTGTTCAAAGCAAGCCTACGCTCTGAATATATTAGCATGGAATAACACGATAGGACTCTGGCTTATCTTGTTGGTCTGTGAGACCAGAGTAATGATTAAGAGGGACAGTCGGGGACATTCGTATTTCATTGTCAGAGGTGAAATTCTTGGATTTATGAAAGACGAACTTCTGCGAAAGCATTTGTCAAGGATGTTTTCATTAATCAAGAACGAAAGTTGGGGGCTCGAAGACGATTAGATACCGTCCTAGTCTCAACCATAAACGATGCCAACTAGGGATTGGCAGATGTTTTTTTGATGACTCTGCCAGCACCTTATGAGAAATCAAAGTTTTTGGGTTCCGGGGGGAGTATGGTCGCAAGGCTGAAACTTAAAGGAATTGACGGAAGGGCACCACCAGGAGTGGAGCCTGCGGCTTAATTTGACTCAACACGG

>24-87

AGCAGGCGCGTAAATTGCCCAATCTTGACACAGGGAGGTAGTGACAATAAATAACAATGCCGGGCCTTTGTAGGTCTGGCAATTGGAATGAGAACAATTTAAACCCCTTATCGAGTATCAATTGGAGGGCAAGTCTGGTGCCAGCAGCCGCGGTAATTCCAGCTCCAATAGCGTATATTAAAGTTGTTGCAGTTAAAAAGCTCGTAGTTGGATTTGTGGCTGTCACGGGCGGCCTATCACTTAGTGTTAGTGCTTGCCTGTGTCGCCATCCTTGGGTGGAGCCCGTGTGGCATTAGGTTGTCGTGCGGGGGATGCCCATCGTTTACTGTGAGAAAATTAGAGTGTTCAAAGCAGGCTTATGCCGTTGAATATGTTAGCATGGAATAATAAGATAGGACCTCGGTACTATTTTGTTGGTTTGCGCACCGAGGTAATGATTAATAGGGACAGTTGGGGGTATTCGTATTTCATTGTCAGAGGTGAAATTCTTGGATTTCTGAAAGACGAACTACTGCGAAAGCATTTACCAAGGATGTTTTCATTAATCAAGAACGAAAGTTAGGGGATCGAAGATGATTAGATACCATCGTAGTCTTAACCATAAACTATGCCGACAAGGGATTGGCGGAGTTTCGTTTTGTCTCCGTCAGCACCTTATGAGAAATCACAAGTCTTTGGGTTCCGGGGGGAGTATGGTCGCAAGGCTGAAACTTAAAGAAATTGACGGAAGGGCACCACCAGGAGTGGAGCCTGCGGCTTAATTTGACTCAACACGG

>24-89

AGCAGGCGCGTAAATTGCCCAATCCTGACACAGGGAGGTAGTGACAATAAATAACAATGCCGGGCCTTTATAGGTCTGGCAATTGGAATGAGAACAATTTAAACCCCTTATCGAGTATCAATTGGAGGGCAAGTCTGGTGCCAGCAGCCGCGGTAATTCCAGCTCCAATAGCGTATATTAAAGTTGTTGCAGTTAAAAAGCTCGTAGTTGGATTTGTGGCTGTCTCGAGCGGCCTGACACCTAGTGTTAGTGCTTGCTTGAGTCGCCATCCTTGGGTGGAACCTGTGTGGCATTAGGTTGTCGTGCAGGGGATGCCCATCGTTTACTGTGAAAAAATTAGAGTGTTCAAAGCAGGCTTATGCCGTTGAATATATTAGCATGGAATAATAAGATAGGACCTTGGTACTATTTTGTTGGTTTGCGCACCAAGGTAATGATTAATAGGGACAGTTGGGGGTATTCGTATTCCATTGTCAGAGGTGAAATTCTTGGATTTCTGGAAGACGAACTACTGCGAAAGCATTTACCAAGGATGTTTTCATTAATCAAGAACGAAAGTTAGGGGATCGAAGATGATTAGATACCATCGTAGTCTTAACCATAAACTATGCCGACAAGGGATTGGCGGAGTCTCGTTTTGTCTCCGTCAGCACCTTATGAGAAATCACAAGTCTTTGGGTTCCGGGGGGAGTATGGTCGCAAGGCTGAAACTTAAAGAAATTGACGGAAGGGCACCACCAGGAGTGGAGCCTGCGGCTTAATTTGACTCAACACGG

>24-98

AGCAGGCGCGAAAATTGCCCAATCTGACACAGGGAGGTAGTGACAATAAATAACAATGCCGGGCCTTTGTAGGTCTGGCAATTGGAATGAGAACAATTTAAACCCCTTATCGAGTATCAATTGGAGGGCAAGTCTGGTGCCAGCAGCCGCGGTAATTCCAGCTCCAATAGCGTATATTAAAGTTGTTGCAGTTAAAAAGCTCGTAGTTGGATTTGTGGCGTACTGAGGCGGCCTGTCACATAGTGTCGGAGCTTGCTTTAGTCGCCATCCTTGGGTGGATCCTGTGTGGCATTAAGTTGTCGTGCAGGGGATGCCCATCTTTTACTGTGAGAAAATTAGAGTGTTCAAAGCAGGCTTATGCCGTTGAATATGTTAGCATGGAATAATAAGATAGGACCTTGGTACTATTTTGTTGGTTTGCGCACCAAGGTAATGATTAATAGGGACAGTTGGGGGTATTCGTATTCCATTGTCAGAGGTGAAATTCTTGGATTTTTGGAAGACGAACTACTGCGAAAGCATTTACCAAGGATGTTTTCATTAATCAAGAACGAAAGTTAGGGGATCGAAGATGATTAGATACCATCGTAGTCTTAACCATAAACTATGCCGACAAGGGATTGGCGGAGTCTCGTTTTGTCTCCGTCAGCACCTTATGAGAAATCACAAGTCTTTGGGTTCCGGGGGGAGTATGGTCGCAAGGCTGAAACTTAAAGAAATTGACGGAAGGGCACCACCAGGAGTGGAGCCTGCGGCTTAATTTGACTCAACACGG

>24-99

AAGCTTGCATGCCTGCAGGTCGACGATTCCGTGTTGAGTCAAATTAAGCCGCAGGCTCCACGCCTGGTGGTGCCCTTCCGTCAATTCCTTTAAGTTTCAGCCTTGCGACCATACTCCCCCCGGAACCCAAAAACTTTGATTTCTCATAAGGTGCTGGCAGAGTCATCAAAAAACATCTGCCAATCCCTAGTCGGCATCGTTTATGGTTGAGACTAGGACGGTATCTAATCGTCTTCGAGCCCCCAACTTTCGTTCTTGATTAATGAAAACATCCTTGACAAATGCTTTCGCAGAAGTTCGTCTTTCATAAATCCAAGAATTTCACCTCTGACAATGAAATACGAATGTCCCCGACTGTCCCTCTTAATCATTACTCTGGTCTCACAGACCAACAAGATAAGCCAGAGTCCTATCGTGTTATTCCATGCTAATATATTCAGAGCGTAGGCTTGCTTTGAACACTCTAATTTACTCAAAGTAACTTCGTCGGCTCCCAGTCCCGGACAGTGAAGCCCAGGAGCTGGTCCCCGACAAGAAGATAGGACTTACCAGTACACATCTCAAAGACGGACCGGCAAATCCTACCCGAAATCCAACTACGAGCTTTTTAACTGCAACAACTTAAATATACGCTATTGGAGCTGGAATTACCGCGGCTGCTGGCACCAGACTTGCCCTCCAATGGATCCTCGTTAAGGGATTTAGATTGTACTCATTCCAATTACCAGACTTAAGAAGCCCGGTATTGTTATTTATTGTCACTGCCTCCCTGAATTAAGATTGGGCAATTTGCGCGCCTGCTAATCTCTAGAGGATCCCCGGGTACCGAGCTCGAATTCGTAATCATGGTCATAGCTGTTTCCTGTGTGAAATTGTTATCCGCTCACAATTCCACACAACATACGAGCCGGAAGCATAAAGTGTAAAGCCTGGGGTGCCTAAT

>24-101

AGCTTGCATGCCTGCAGGTCGACGATTCCGTGTTGAGTCAAATTAAGCCGCAGGCTCCACTCCTGGTGGTGCCCTTCCGTCAATTCCTTTAAGTTTCAGCCTTGCGACCATACTCCCCCCAGAACCCAAAGACTTTGATTTCTCATAAGATGCCGAAGGAAGCTAATTGATATCCACCGATCTCTAGTCGGCATAGTTTATGGTTAAGACTACGACGGTATCTGATCGTCTTCGATCCCCTAACTTTCGTTCTTGATTAATGAAAACATCCTTGGTAGATGCTTTCGCACTAGTTCGTCCTGAACAAATCTAAGAATTTCACCTCTGACTGTTCAATACGAATACCCCCGACTGTTCCTCTCAACACGCACCGCATTTCCGAGAGTTGAAAAACGGAAATGCAGCCTTTTCGTCATTCCACGGTACACTATTTCAGCAAATGCCCGCTTGAAACACTCTATTTTTCTCAAAGTAACTGAGCATCGTGAGATGCCTGGCAAGCAAAAGGTTTTCCAGTGGAAATGCCGATCACAAGCCATCATTTCAACTACGAGCTTTTTAACTGCAACAACTTTAATATACGCTATTGGAGCTGGAATTACCGCGGCTGCTGGCACCAGACTTGCCCTCCAATTGATACTCTAAGCGAGATGTAACCGCCTGCCATTGCAAACATCAAAGCAAGTGCTTTGTGTTGCTATTTCTTGTCACTACCTCCCTGTGTCAAGATTGGGCAATTTGCGCGCCTGCTAATCTCTAGAGGATCCCCGGGTACCGAGCTCGAATTCGTAATCATGGTCATAGCTGTTTCCTGTGTGAAATTGTTATCCGCTCACAATTCCACACAACATACGAGCCGGAAGCATAAAGTGTAAAGCCTGGGGTGCCTAATGAGTGAGCTAACTCACATTAATTGCGTTGCGCTCACTGCCCGCTTTCCAG

>24-104

AAGCTTGCATGCCTGCAGGTCGACGATTCCGTGTTGAGTCAAATTAAGCCGCAGGCTCCACTCCTGGTGGTGCCCTTCCGTCAATTTCTTTAAGTTTCAGCCTTGCGACCATACTCCCCCCGGAACCCAAAAACTTTGATTTCTCATGAGGTGCTGGCGGCGTCATCTTGAGACAGTCCGCCAATCCCTAGTCGGCATCGTTTATGGTTGAGACTACGACGGTATCTAATCGTCTTCGAGCCCCCAACTTTCGTTCTTGATCAATGAAAACATCCTTGGCAAATGCTTTCGCAGATGTTCGTCTTCCATCAATCACTGAATTTCACCTCTGACAACGGAATACGAATGCCCCCGACTGTCCCTCTTAATCATTACTCTGGTCCTATAGACCAACACGATAGGCCAGAGTCCTGTCGTGTTATCCCATGCTATTATATTCAGAGCGTAAGCTTGCTTTGAACACTCTAATTTACTCAAAGTAACATCGCCGATTCTGACCCTCAGACAGTGAAGTCCGACGGGCCGCCCCCGGCAAGAAGGAGGGCTGCAGCAGTACAAACGCAATGCGAGACCGCTGCGTCCCACCCGAAATCCAACTACGAGCTTTTTAACTGCAACAACTTAAATATACGCTATTGGAGCTGGAATTACCGCGGCTGCTGGCACCAGACTTGCCCTCCAATGGATCCTCGTTAAGGCGTTTACATTATACTCATTCCAATTACCGGACCATGTGGCCCAGAATTGATATTTATTGTCACTACCTCCCTGTGTCAAGATTGGGCAATTTGCGCGCCTGCTAATCTCTAGAGGATCCCCGGGTACCGAGCTCGAATTCGTAATCATGGTCATAGCTGTTTCCTGTGTGAAATTGTTATCCGCTCACAATTCCACACAACATACGAGCCGGAAGCATAAAGTGTAAAGCCTGGGGTGC

>24-105

CAGCTTGCATGCCTGCAGGTCGACGATTCCGTGTTGAGTCAAATTAAGCCGCAGGCTCCACTCCTGGTGGTGCCCTTCCGTCAATTCCTTTAAGTTTCGGATTTGCAACCATACTTCCCCCGGAACCGAAAAACTTATCGTTTCCGGGCAGCCGCCCGCTGAGTCAGAATTAACGCCAGCGGATTGCTAGTTGGCATCGTTTACGGTTAGAACTAGGGCGGTATCTGATCGCCTTCGAACCTCTAACTATCGTTCTTGATTAATGAAAACATTCTTGGCAAATGCTTTCGCAGTTGTTCGTCATGCTACGATCCAAGAATTTCACCTCTAACGCAGCAGTACGAATGCCCCCGTCTGTCCCTCTTAATCATTACCTCGTGCTCCGAAAACCAACAAAATAGAACCGAGGTCCTATTCCATTATTCCATGCACCACTATTCAGGCAAAAGCCTGCGTTAAGCACTCTAATTTTTTCAAAGTAAACGTGCCGATCACCCAGGGCACTCAGTAAAGAGCACCAAGGGAAAACCGACAAGGTAGGTCAAGACAACAAGTATTCCGAGAAGGAAACCAGTGTCTGTGACCGCAGATCCAACTACGAGCTTTTTAACCGCAACAACTTTAATATACGCTATTGGAGCTGGAATTACCGCGGCTGCTGGCACCAGACTTGCCCTCCAATAGATCCTCGTTAAAGGGTTTAAATTGTACCCATTCCGATGACAGACCTCATAGAAGACTGTCTCGTTATTTTTCGTCACTACCTCCTCGTTCTGAGATTGGGCAATTTACGCGCCTGCTAATCTCTAGAGGATCCCCGGGTACCGAGCTCGAATTCGTAATCATGGTCATAGCTGTTTCCTGTGTGAAATTGTTATCCGCTCACAATTCCACACAACATACGAGCCGGAAGCATAAAGTGTAAAGCCTGGGG

>24-107

AGCAGGCGCGCAAATTGCCCAATCCTGACACAGGGAGGTAGTGACAATAAATAACAATGCCGGGCCATCATTGGTCTGGCAATTGGAATGAGAACAATTTAAATCCCTTATCGAGGATCAATTGGAGGGCAAGTCTGGTGCCAGCAGCCGCGGTAATTCCAGCTCCAATAGCGTATATTAAAGTTGTTGCGGTTAAAAAGCTCGTAGTTGGATTTCTGTTGGGGACGACCGGTCCGCCTTCTGGGTGAGCATCTGGTTCGGCCTTGGCATCTTTCTAGCGAACGGTCTGTACTTGACTGTATGGGTCGGTAGTTAGGACTTTTACCTTGAGAAAATTAGAGTGTTTCAGGCAGGCTTGCGCCCTGAATACATTAGCATGGAATAATAATATAGGACTCAAGTTCTATTTTGTTGGTTTCTAGTGCTTGAGTAATGATTGATAGGGATAGTTGGGGGCATTCGTATTTAACTGTCAGAGGTGAAATTCTTGGATTTGTTAAAGACGGACTACTGCGAAAGCATTTGCCAAGGATGTTTTCATTAATCAAGAACGAAAGTTAGGGGATCGAAGATGATTAGATACCATCGTAGTCTTAACCATAAACTATGCCGACAAGGGATTGGCGGGCGTTGTATTGACTCCGTCAGCACCTTATGAGAAATCACAAGTCTTTGGGTTCCGGGGGGAGTATGGTCGCAAGGCCGAAACTTAAAGAAATTGACGGAAGGGCACCACCAGGAGTGGAGCCTGCGGCTTAATTTGACTCAACACGG

>24-108

AGCAGGCGCGTAAATTGCCCAATCTTGATACGGGGAGGTAGTGACAAGAAATAACAATACGGGGCCTACTAGGTCTTGTAATTGGAATGAGTACAATCTAAATCCCTTAACGAGGATCAATTGGAGGGCAAGTCTGGTGCCAGCAGCCGCGGTAATTCCAGCTCCAATAGCGTATATTAAAGTTGTTGCAGTTAAAAAGCTCGTAGTTGGATTTCTGGTAGGAGCGACCATACCGGACTTGATTGTCCGCGTAATGCGTTGTCTCCAGCCATCCTCGTGGAGATTGTTTTTGGCATTAACTTGTCGAATTCGGGACCCGCGTCATTTACTGTGAAAAAATTAGAGTGTTTAAAGCAGGCAATTGGCTTGAATACATTAGCATGGAATAATAAGATAGGACCTTGGTACTATTTTGTTGGTTTGCGCACCAAGGTAATGATTAATAGGGACAGTTGGGGGTATTCGTATTCCATTGTCAGAGGTGAAATTCTTGGATTTTTGGAAGACGAACTACTGCGAAAGCATTTACCAAGGATGTTTTCATTAATCAAGAACGAAAGTTAGGGGATCGAAGATGATTAGATACCATCGTAGTCTTAACCATAAACTATGCCGACAAGGGATTGGCGGGGTTTCGTTATGTCTCCGCCAGCACCTTATGAGAAATCACAAGTCTTTGGGTTCCGGGGGGAGTATGGTCGCAAGGCTGAAACTTAAAGAAATTGACGGAAGGGCACCACCAGGAGTGGAGCCTGCGGCTTAATTTGACTCAACACGG

>24-109

AGCAGGCGCGTAAATTGCCCAATCTTGATACGGGGAGGTAGTGACAATAAATAACAATACGGGGCCCATCGGGTCTTGTAATTGGAATGAGTACAATCTAAATCCCTTAACGAGGAACAATTGGAGGGCAAGTCTGGTGCCAGCAGCCGCGGTAATTCCAGCTCCAATAGCGTATATTTAAGTTGTTGCAGTTAAAAAGCTCGTAGTTGGATTTCGGATGGGATCTGCCGGTCCGTCTTTGAGATGTGTACTGGTAGGTCCTATCTTGTTGTCGGGGACCAGCTCCTGGGCTTCACTGTCCGGGACTGGGAGCCGACGAAGTTACTTTGAGTAAATTAGAGTGTTCAAAGCAAGCCTACGCTCTGAATATATTAGCATGGAATAACACGATAGGACTCTGGCTTATCTTGTTGGTCTGTGAGACCAGAGTAATGATTAAGAGGGACAGTCGGGGACATTCGTATTTCATTGTCAGGGGTGAAATTCTTGGATTTATGAAAGACGAACTTCTGCGAAAGCATTTGTCAAGGATGTTTTCATTAATCAAGAACGAAAGTTGGGGGCTCGAAGACGATTAGATACCGTCCTAGTCTCAACCATAAACGATGCCGACTAGGGATTGGCAGATGTTTTTTTGATGACTCTGCCAGCACCTTATGAGAAATCAAAGTTTTTGGGTTCCGGGGGGAGTATGGTCGCAAGGCTGAAACTTAAAGGAATTGACGGAAGGGCACCACCAGGCGTGGAGTCTGCGGCTTAATTTGACTCAACACGG

>24-111

AGCTTGCATGCCTGCAGGTCGACGATAATCTCTAGAGGATCCCCGGGTACCGAGCTCGAATTCGTAATCATGGTCATAGCTGTTTCCTGTGTGAAATTGTTATCCGCTCACAATTCCACACAACATACGAGCCGGAAGCATAAAGTGTAAAGCCTGGGGTGCCTAATGAGTGAGCTAACTCACATTAATTGCGTTGCGCTCACTGCCCGCTTTCCAGTCGGGAAACCTGTCGTGCCAGCTGCATTAATGAATCGGCCAACGCGCGGGGAGAGGCGGTTTGCGTATTGGGCGCTCTTCCGCTTCCTCGCTCACTGACTCGCTGCGCTCGGTCGTTCGGCTGCGGCGAGCGGTATCAGCTCACTCAAAGGCGGTAATACGGTTATCCACAGAATCAGGGGATAACGCAGGAAAGAACATGTGAGCAAAAGGCCAGCAAAAGGCCAGGAACCGTAAAAAGGCCGCGTTGCTGGCGTTTTTCCATAGGCTCCGCCCCCCTGACGAGCATCACAAAAATCGACGCTCAAGTCAGAGGTGGCGAAACCCGACAGGACTATAAAGATACCAGGCGTTTCCCCCTGGAAGCTCCCTCGTGCGCTCTCCTGTTCCGACCCTGCCGCTTACCGGATACCTGTCCGCCTTTCTCCCTTCGGGAAGCGTGGCGCTTTCTCATAGCTCACGCTGTAGGTATCTCAGTTCGGTGTAGGTCGTTCGCTCCAAGCTGGGCTGTGTGCACGAACCCCCCGTTCAGCCCGACCGCTGCGCCTTATCCGGTAACTATCGTCTTGAGTCCAACCCGGTAAGACACGACTTATCGCCACTGGCAGCAGCCACTGGTAACAGGATTAGCAGAGCGAGGTATGTAGGCGGTGCTACAGAGTTCTTGAAGTGGTGGCCTAACTACGGCTACACTAGAAGAACAGTATTTGGTATCTGCGCTCTGCTGAG

>24-115

AGCTTGCATGCCTGCAGGTCGACGATTATCTCTAGAGGATCCCCGGGTACCGAGCTCGAATTCGTAATCATGGTCATAGCTGTTTCCTGTGTGAAATTGTTATCCGCTCACAATTCCACACAACATACGAGCCGGAAGCATAAAGTGTAAAGCCTGGGGTGCCTAATGAGTGAGCTAACTCACATTAATTGCGTTGCGCTCACTGCCCGCTTTCCAGTCGGGAAACCTGTCGTGCCAGCTGCATTAATGAATCGGCCAACGCGCGGGGAGAGGCGGTTTGCGTATTGGGCGCTCTTCCGCTTCCTCGCTCACTGACTCGCTGCGCTCGGTCGTTCGGCTGCGGCGAGCGGTATCAGCTCACTCAAAGGCGGTAATACGGTTATCCACAGAATCAGGGGATAACGCAGGAAAGAACATGTGAGCAAAAGGCCAGCAAAAGGCCAGGAACCGTAAAAAGGCCGCGTTGCTGGCGTTTTTCCATAGGCTCCGCCCCCCTGACGAGCATCACAAAAATCGACGCTCAAGTCAGAGGTGGCGAAACCCGACAGGACTATAAAGATACCAGGCGTTTCCCCCTGGAAGCTCCCTCGTGCGCTCTCCTGTTCCGACCCTGCCGCTTACCGGATACCTGTCCGCCTTTCTCCCTTCGGGAAGCGTGGCGCTTTCTCATAGCTCACGCTGTAGGTATCTCAGTTCGGTGTAGGTCGTTCGCTCCAAGCTGGGCTGTGTGCACGAACCCCCCGTTCAGCCCGACCGCTGCGCCTTATCCGGTAACTATCGTCTTGAGTCCAACCCGGTAAGACACGACTTATCGCCACTGGCAGCAGCCACTGGTAACAGGATTAGCAGAGCGAGGTATGTAGGCGGTGCTACAGAGTTCTTGAAGTGGTGGCCTAACTACGGCTACACTAGAAGAACAGTATTTGGTATCTGCGCTCTGCTGAAG

>24-118

AGCAGGCGCGTAAATTGCCCAATCTTGACACAGGGAGGTAGTGACAAGAAATAACAATGCAGGGCATCCATGTCTTGCAATTGGAATGAGTAGAATTTAAATCCCTCTATGAGTATCAATTGGAGGGCAAGTCTGGTGCCAGCAGCCGCGGTAATTCCAGCTCCAATAGCGTATATTAAAGTTGTTGCGGTTAAAAAGCTCGTAGTTGGATTTCTGCCGAGGACGACCGGTCCACCCTCTGGGTGAGCATCTGGCTCGGCTTGGGCATCTTCTTGGGGAGCGTATCTGCGCTTCATTGTGCGGTGCGGTACCCGAGACTCTTACTTTGAGGAAATTAGAGTGTTTCAAGCAGGCTCACGCCTTGAATACATTAGCATGGAGTAATAGAATTGGACCTCGGCCCTTTTTTGTTGGTTTCTAGAGCTGAGGTAAGGATTAATAGGGATAGTTGGGGGCATTCGTATCTAACTGTCAGAGGTGAAATTCTTGGATTTGTTAAAGACGGACTAATGCGAAAGCATTTGCCAAGGATGTTTTCATTGACCAAGAACGAAAGTTAGGGGATCGAAGACGATCAGATACCGTCCTAGTCTTAACCATAAACCATGCCGACTAGGGATTGGAGGTCGTTATCCATATGACCCCTTCAGCATCTTATGAGAAATCAAAGTCTTTGGGTTCCGGGGGGAGTATGGTCGCAAGGCTGAAACTTAAAGGAATTGACGGAAGGGCACCACCAGGAGTGGAGCCTGCGGCTTAATTTGACTCAACACGG

>24-119

AGCAGGCGCGAAAATTGCCCAATCCTGACACAGGGAGGTAGTGACAATAAATAACAATGCCGGGCCTTTGTAGGTCTGGCAATTGGAATGAGAACAATTTAAACCCCTTATCGAGTATCAATTGGAGGGCAAGTCTGGTGCCAGCAGCCGCGGTAATTCCAGCTCCAATAGCGTATATTAAAGTTGTTGCAGTTAAAAAGCTCGTAGTTGGATTTGTGGTGTACTTCGGCGGCCCGTCACTATGTGATGGAGCTTGCTGAAGTCGCCATCCTTGGGTGGATCCTGTGTGGCATTAAGTTGTCGTGCAGGGGATGCCCATCGTTTACTGTGAAAAAATTAGAGTGTTCAAAGCAGGCTTATGCCGTTGAATATATTAGCATGGAATAATAAGATAGGACCACAGCGGTCTATTTTGTTGGTTTGCGCACCAAGGTAATGATTAATAGGGACAGTTGGGGGTATTCGTATTTCATTGTCAGAGGTGAAATTCTTGGATTTTTGAAAGACGAACTACTGCGAAAGCATTTACCAAGGATGTTTTCATTAATCAAGAACGAAAGTTAGGGGATCGAAGATGATTAGATACCATCGTAGTCTTAACCATAAACTATGCCGACAAGGGATTGGCGGGCGTTGTATTGACTCCGTCAGCACCTTATGAGAAATCACAAGTCTTTGGGTTCCGGGGGGAGTATGGTCGCAAGGCTGAAACTTAAAGAAATTGACGGAAGGGCACCACCAGGAGTGGAGCCTGCGGCTTAATTTGACTCAACACGG

>24-121

AGCAGGCGCGAAAATTGCCCAATCTTGACACAGGGAGGTAGTGACAATAAATAACAATGCCGGGCCATCATTGGTCTGGCAATTGGAATGAGAACAATTTAAATCCCTTATCGAGGATCAATTGGAGGGCAAGTCTGGTGCCAGCAGCCGCGGTAATTCCAGCTCCAATAGCGTATATTAAAGTTGTTGCAGTTAAAAAGCTCGTAGTTGGATTTGTGGCGTACGGTGTGTTCCGGGCACTTGTTGTCTGAGTAACCTGCTGTTGCCATCCTTGGGTGGAACCTGTGTGGCATTAAGTTGTCGTGCAGGGGATGCCCATCATTTACTGTGAAAAAATTAGAGTGTTCAAAGCAGGCTTACGCCGCTGAATATATTAGCATGGAATAATGAGATAGGACCTTGGTACTATTTTGTTGGTTTGCGCACCGAGGTAATGATTAATAGGGACAGTTGGGGGTATTCGTATTCCATTGTCAGAGGTGAAATTCTTGGATTTCTGGAAGACGAACTACTGCGAAAGCATTTACCAAGGATGTTTTCATTAATCAAGAACGAAAGTTAGGGGATCGAAGATGATTAGATACCATCGTAGTCTTAACCATAAACTATGCCGACAAGGGATTGGTGGAGTCTCGTTACGTCTCCATCAGCACCTTAGGAGAaATCATAAGTCTTTGGGTTCCGGGGGGAGTATGGTCGCAAGGCTGAAACTTAAAGAAATTGACGGAAGGGCACCACCAGGAGTGGAGCCTGCGGCTTAATTTGACTCAACACGG

>24-122

AGCAGGCGCGTAAATTGCCCAATCCTGACACAGGGAGGTAGTGACAATAAATAACAATGCCGGGCCTTTACAGGTCTGGCAATTGGAATGAGAACAATTTAAATCCCTTATCGAGTATCAATTGGAGGGCAAGTCTGGTGCCAGCAGCCGCGGTAATTCCAGCTCCAATAGCGTATATTAAAGTTGTTGCAGTTAAAAAGCTCGTAGTTGGATTTCTGGCAGGAGGGACCGGTCACGCACTCTGTGTGTGAACTTGGCTCCCTCTGGCCATCCTTGGGGAGATCCTGTTTGGCATTAAGTTGTCGGGCAGGGGATACCCATCGTTTACTGTGAAAAAATTAGAGTGTTTAAAGCAGGCTTATGCCGTTGAATATATTAGCATGGAATAATAAGATAGGACTTCGGAACTATTTTGTTGGTTTGCGTTACGAAGTAATGATTAATAGGGACAGTTGGGGGTATTCGTATTTCGTTGTCAGAGGTGAAATTCTTGGATTTCCGAAAGACGAACTACTGCGAAAGCATTTACCAAGGATGTTTTCATTAATCAAGAACGAAAGTTAGGGGATCGAAGATGATTAGATACCATCGTAGTCTTAACCATAAACTATGCCGACTCAGGATTGGCGGTTGTTTTTTGACTCCGTCAGCACTGTATGAGAAATCAAAGTCTTTGGGTTCCGGGGGGAGTATGGTCGCAAGGCTGAAACTTAAAGAAATTGACGGAAGGGCACCACCAGGAGTGGAACCTGCGGCTTAATTTGACTCAACACGG

>24-123

AGCAGGCGCGTAAATTGCCCAATCTTAATTCAGGGAGGTAGTGACAATAAATAACAATACCGGGCTTCTTAAGTCTGGTAATTGGAATGAGTACAATCTAAATCCCTTAACGAGGATCCATTGGAGGGCAAGTCTGGTGCCAGCAGCCGCGGTAATTCCAGCTCCAATAGCGTATATTTAAGTTGTTGCAGTTAAAAAGCTCGTAGTTGGATTTCGGATGGGATCTGCCGGTCCGTCTTTGAGATGTGTACTGGTAGGTCCTATCTTGTTGTCGGGGACCAGCTCCTGGGCTTCACTGTCCGGGACTGGGAGCCGACGAAGTTACTTTGAGTAAATTAGAGTGTTCAAAGCAAGCCTACGCTCTGAATATATTAGCATGGAATAACACGATAGGACTCTGGCTTATCTTGTTGGTCTGTGAGACCAGAGTAATGATTAAGAGGGACAGTCGGGGACATTCGTATTTCATTGTCAGAGGTGAAATTCTTGGATTTGTTAAAGACGGACGACTGCGATAGCATTTGCCAAGGATGTTTTCATTGATCAAGAACGAAAGTTAGGGGATCGAAGACGATCAGATACCGTCGTAGTCTTAACCATAAACCATGCCGACTAGAGATTGGAGGTCGTTTTCCACACGACCCCTTCAGCACCTTATGAGAAATCAAAGTCTTTGGGTTCCGGGGGGAGTATGGTCGCAAGGCTGAAACTTAAAGGAATTGACGGAAGGGCACCACCAGGAGTGGAGCCTGCGGCTTAATTTGACTCAACACGG

>24-125

AGCTTGCATGCCTGCAGGTCGACGATTCCGTGTTGAGTCAAATTAAGCCGCAGGCTCCACTCCTGGTGGTGCCCTTCCGTCAATTTCTTTAAGTTTCAGCCTTGTGACCACACTCCCCCCGGAACCCAAAGACTTGTGATTTCTCATGAGGTGCTGATGGAGACATGACGAGACTCCACCAATCCTTTGTCGGCACAGTTCATGGTTAAGACTGCGATGGTGTCTAATCATCTTCGATCCCCTAACTTTCGTTCTTGATTAATAAAAACATCCTTGGTAAATGCTTTCGCAGTAGTTCGTCTTCCAAAAATCCAAGAATTTCACCTCTGACAATGGAATACGAATACCCCCAACTGTCCCTGTTAATCATTGCCTCTTTGCGCAAACCAACAAAATAGCAAAAAAGTCCCATCTCATCATTCCATGCTAATGCATTCAACGGCATGAGCCTGCTTTGAACACTCTAATTTTTTCACAGTGAATGATGGGCATCCCCTGCACGACAACCTAATGCCACACAGGTTCCACCCAAGGATGGCAACAGCAGGTTACTCAGACAACAGTTGCCCGGAACACACCGTGCGCCACAAATCCAACTACGAGCTTTTTAACTGCAACAACTTTAATATACGCTATTGGAGCTGGAATTACCGCGGCTGCTGGCACCAGACTTGCCCTCCAATTGATACTCGATAAGGGGTTTAAATTGTTCTCATTCCAATTGACAGACCTACAAAGGCCCGTCATTGTTATTTATTGTCACTACCTCCCTGTGTCAAGATTGGGCAATTTGCGCGCCTGCTAATCTCTAGAGGATCCCCGGGTACCGAGCTCGAATTCGTAATCATGGTCATAGCTGTTTCCTGTGTGAAATTGTTATCCGCTCACAATTCCACACaACATACGAGCCGGAAGCATAAGTGTAAAGCCTGGGGTGCCTAATGA>24-126

AGCAGGCGCGCAAATTGCCCAATCCTGAAAAAGGGAGGTAGTGACAAAAAATAGCAACTCAAAGCTTTAGCTTTGCTGATTGCAATGGGGTTCGGCTAAAGCTCGTTCCTAGAATCAATTGGAGGGCAAGTCTGGTGCCAGCAGCCGCGGTAATTCCAGCTCCAATAGCGTATATTTAAGTTGTTGCAGTTAAAAAGCTCGTAGTTGTACCGATGCGCCGGGTAAGCTAGTTCTTTTTAGAGCTTCTTCCTTGCGTGGGTCTTTCGTAGACCTCTGTCACTTTGAGAAAAATTGAGTGTTTCAGGCGGGCCAGTGCTGAAATAGTGTACCATGGAATGACGAAGTGGCTGCCTCTCTGTTTATTTGGCTTTAGAGAGGCAGCGCGTGGTTGAGAGAAACAGTCGGGGGTATTCGTATTGAACAGTCAGAGGTGAAATTCTTAGATTTGTTCAAGACGAACCAATGCGAAAGCATCTACCATGGATGTTTTCATTAATCAAGAACGAAAGTTAGGGGATCCAAGACGATCAGATACCGTCGTAGTCTTAACCGTAAACTATGCCGACTAGAGATCGGTGGAAATCTAATCAGATTCCTTCGGCACCTTATGAGAAATCAAAGTTCTTGGGTTCTGGGGGGAGTATGGTCGCAAGGCTGAAACTTAAAGGAATTGACGGAAGGGCACCACCAGGAGTGGAGCCTGCGGCTTAATTTGACTCAACACGG

>24-133

AGCAGGCGCGAAAATTGCCCAATCCTGACACAGGGAGGTAGTGACAATAAATAACAATGCCGGGCCTTTGTAGGTCTGGCAATTGGAATGAGAACAATTTAAACCTCTTATCGAGTATCAATTGGAGGGCAAGTCTGGTGCCAGCAGCCGCGGTAATTCCAGCTCCAATAGCGTATATTAAAGTTGTTGCAGTTAAAAAGCTCGTAGTTGGATTTGTGGCGTACGGTGTGTTCCGGGCACTTGTTGTCTGAGTAACCTGCTGTTGCCATCCTTGGGTGGAACCTGTGTGGCATTAGGTTGTCGTGCAGGGGATGCCCATCGTTTACTGTGAAAAAATTAGAGTGTTCAAAGCAGGCTTATGCCGTTGAATATATTAGCATGGAATAATGAGATAGGACTTTTTTGCTATTTTGTTGGTTTGCGCGAAGAGGTAATGATTAATAGGGACAGTTGGGGGTATTCGTATTCCATTGTCAGAGGTGAAATTCTTGGATTTCTGGAAGACGAACTACTGCGAAAGCATTTACCAAGGATGTTTTCATTAATCAAGAACGAAAGTTAGGGGATCGAAGATGATTAGATACCATCGTAGTCTTAACCATAAACTATGCCAACAAGGGATTGGTGGAGTCTCGTTACGTCTCCATCAGCACCTTATGAGAAATCACAAGTCTTTGGGTTCCGGGGGGAGTATGGTCGCAAGGCTGAAACTTAAAGAAATTGACGGAAGGGCACCACCAGGAGTGGAGCCTGCGGCTTAATTTGACTCAACACGG

>24-134

AGCTTGCATGCCTGCAGGTCGACGATTCCGTGTTGAGTCAAATTAAGCCGCAGGCTCCACTCCTGGTGGTGCCCTTCCGTCAATTTCTTTAAGTTTCAGCCTTGCGACCACACTCCCCCCGGAACCCAAAGACTTGTGATTTCTCATAAGGTGCTGATGGAGACATAACGAGACTCCACCAATCCCTTGTCGGCATAGTTTATGGTTAAGACTACGATGGTATCTAATCATCTTCGATCCCCTAACTTTCGTTCTTGATTAATGAAAACATCCTTGGTAAATGCTTTCGCAGTAGTTCGTCTTCCAAAAATCCAAGAATTTCACCTCTGACAATGGAATACGAATACCCCCAACTGTCCCTATTAATCATTACCTCTTTGCGCAAACCAACAAAATAGCAAAAAAGTCCTATCTCATTATTCCATGCTAATATATTCAACGGCATAAGCCTGCTTTGAACACTCTAATTTTTTCACAGTAAATGATGGGCATCCCCTGCACGACAACCTAATGCCACACAGGTTCCACCCAAGGATGGCAACAGCAGGTTACTCAGACAACAAGTGCCCGGAACACACCGTACGCCACAAATCCAACTACGAGCTTTTTAACTGCAACAACTTTAATATACGCTATTGGAGCTGGAATTACCGCGGCTGCTGGCACCAGACTTGCCCTCCAATTGATACTCGATAAGGGGTTTAAATTGTTCTCATTCCAATTGCCAGACCTACAAAGGCCCGGCATTGTTATTTATTGTCACTACCTCCCTGTGTCAGGATTGGGCAATTTACGCGCCTGCTAATCTCTAGAGGATCCCCGGGTACCGAGCTCGAATTCGTAATCATGGTCATAGCTGTTTCCTGTGTGAAATTGTTATCCGCTCACAATTCCACACAACATACGAGCCGGAAGCATAAAGTGTAAAGCCTGGGGTGCCTAATGAG

>24-137

AGCAGGCGCGCAAATTGCCCAATCTTGACACAGGGAGGTAGTGACAATAAATAACAATGCCGGGCCATCATTGGTCTGGCAATTGGAATGAGAACAATTTAAATCCCTTATCGAGGATCAATTGGAGGGCAAGTCTGGTGCCAGCAGCCGCGGTAATTCCAGCTCCAATAGCGTATATTAAAGTTGTTGCAGTTAAAAAGCTCGTAGTTGGATTTGTGGCGTACGGTGTGTTCCGGGCACTTGTTGTCTGAGTAACCCGCTGTTGCCATCCTTGGGTGGAACCTGTGTGGCATTAGGTTGTCGTGCAGGGGATGCCCATCATTTACTGTGAAAAAATTAGAGTGTTCAAAGCAGGCTTATGCCGTTGAATATATTAGCATGGAATAATGAGATAGGACTTTTTTGCTATTTTGTTGGTTTGCGCAAGAAGGTAATGATTAATAGGGACAGTTGGGGGTATTCGTATTCCATTGTCAGAGGTGAAATTCTTGGATTTTTGGAAGACGAACTACTGCGAAAGCATTTACCAAGGATGTTTTCATTAATCAAGAACGAAAGTTAGGGGATCGAAGATGATTAGATACCATCGTAGTCTTAACCATAAACTATGCCGACAAGGGATTGGTGGAGTCTCGTTATGTCTCCATCAGCACCTTATGAGAAATCACAAGTCTTTGGGTTCCGGGGGGAGTATGGTCGCAAGGCTGAAACTTAAAGAAATTGACGGAAGGGCACCACCAGGAGTGGAGCCTGCGGCTTAATTTGACTCAACACGG

>24-140

AGCAGGCGCGCAAATTACCCAATCCTAATTCAGGGAGGTAGTGACAATAAATAACAATACCGGGCTTCTTAAGTCTGGTAATTGGAATGAGTACAATCTAAGTCCCTTAACGAGGATCCATTGGAGGGCAAGTCTGGTGCCAGCAGCCGCGGTAATTCCAGCTCCAATAGCGTATATTTAAGTTGTTGCAGTTAAAAAGCTCGTAGTTGGATTTCGGGTGGGCGGTGCCGGTCCGCCGTTTCGGTGCGCACTGGTTCTGCCCACCTTGCTGCCGGGGACGTGTTCCTGGGCTTCGTTGTCCGGGACACGGCGTCGGCGAGGTTACTTTGAGTAAATTAGAGTGTTCAAAGCAGGCCTACGCTCTGAATACATTAGCATGGAATAACACGATAGGACTCTGGCCTATCTTGTTGGTCTGTAGGACCGGAGTAATGATTAAGAGGGACAGTCGGGGGCATTCGTATTTCATTGTCAGAGGTGAAATTCTTGGATTTATGAAAGACGAACTACTGCGAAAGCATTTGCCAAGGATGTTTTCATTAATCAAGAACGAAAGTTGGGGGCTCGAAGACGATTAGATACCGTCCTAGTCTCAACCATAAACGATGCCGACTAGGGATCGGCGGGTGTTTTTTTGATGACCCCGCCGGCACCTTATGAGAAATCAAAGTTTTTGGGTTCCGGGGGGAGTATGGTCGCAAGGCTGAAACTTAAAGGAATTGACGGAAGGGCACCACCAGGCGTGGAGCCTGCGGCTTAATTTGACTCAACACGG

>24-141

AGCAGGCGCGTAAATTGCCCAATCCTGACACAGGGAGGTAGTGACAATAAATAACAATGCCGGGCCATCATTGGTCTGGCAATTGGAATGAGAACAATTTAAATCCCTTATCGAGGATCAATTGGAGGGCAAGTCTGGTGCCAGCAGCCGCGGTAATTCCAGCTCCAATAGCGTATATTAAAGTTGTTGCAGTTAAAAAGCTCGTAGTTGGATTTGTGGCGTACGGTGTGTTCCGGGCACTTGTTGTCTGAGTAACCTGCTGTTGCCATCCTTGGGTGGAACCTGTGTGGCATTAGGTTGTCGTGCAGGGGATGCCCATCATTTACTGTGAAAAAATTAGAGTGTTCAAAGCAGGCTTATGCCGTTGAATATATTAGCATGGAATAATGAGATAGGACTTTTTTGCTATTTTGTTGGTTTGCGCAAAGAGGTAATGATTAATAGGGACAGTTGGGGGTATTCGTATTCCATTGTCAGAGGTGAAATTCTTGGATTTTTGGAAGACGAACTACTGCGAAAGCATTTACCAAGGATGTTTTCATTAATCAAGAACGAAAGTTAGGGGATCGAAGACGATTAGATACCATCGTAGTCTTAACCATAAACTATGCCGACAAGGGATTGGTGGAGTCTCGTTATGTCTCCATCAGCACCTTATGAGAAATCACAAGTCTTTGGGTTCCGGGGGGAGTATGGTCGCAAGGCTGAAACTTAAAGAAATTGACGGAAGGGCACCACCAGGAGTGGAGCCTGCGGCTTAATTTGACTCAACACGG

>24-144

AAGCTTGCATGCcTGcAGgTCGACGATTCCGTGTTGAGTCAAATTAAGCCGCAAGCTCCACTCCTGGTGGTGCCCTTCCGTCAATTTCTTTAAGTTTCAGCCTTGCGACCATACTCCCCCCGGAACCCAAAGACTTGTGATTTCTCATAAGGTGCTGATGGAGACGAAACGAAACTCCACCAATCCCTTGTCGGCATAGTTTATGGTTAAGACTACGATGGTATCTAATCATCTTCGATCCCCTAACTTTCGTTCTTGATTAATGAAAACATCCTTGGTAAATGCTTTCGCAGTAGTTTGTCTTCCAAAAATCCAAGAATTTCACCTCTGACAATGGAATACAAATACCCCCAACTGTCCCTATTAATCATTACCTCAGTGCGCAAACCAACAAAATAGTACCAAGGTCCTATATCATTATTCCATGCTAATACGTTCAACGGCATAAGCCTGCTTTGAACACTCCGATTTTTTCACAGTAAAAGATGGGCACCGACAATGCGACAACTTAATGCCACAAAGCCAACAATCCCATAAAAGGTGGACCCGTACAGCAGAGAAGGCTTAGCCGACTCACCGACAGGACGCACCACAAATCCAACTACGAGCTTTTTAACTGCAACAACTTTAATATACGCTATTGGAGCTGGAATTACCGCGGCTGCTGGCACCAGGCTTGCCCTCCAATTGATACTCGATAAGGGGTTTAAATTGTTCTCATTCCAATTGCCAGACCTACAAGGCCCGGGATTGTTATTTATTGTCACTACCTCCCCGAGTCGAGATTGGGCAATTTACGCGCCTGCTAATCTCTAGAGGATCCCCGGGTACCGAGCTCGAATTCGTAATCATGGTCCATAGCTGTTTCCTGTGTGAGATTGTTATCCCGCTCACCACTACACACAACATACGAGCCGCAGCATAAAGTAGTCAAGCCTGGGCGT

>24-146

AGCTTGCATGCCTGCAGGTCGACGATTCCGTGTTGAGTCAAATTAAGCCGCAGGCCCCACGCCTGGTGGTGCCCTTCCGTCAATTCCTTTAAGTTTCAGCCTTGCGACCATACTCCCCCCGGAACCCAAAAACTTTGATTTCTCATAAGGTGCTGGCAGAGTCATCAAAAAAACATCTGCCAATCCCTAGTCGGCATCGTTTATGGTTGAGACTAGGACGGTATCTAATCGTCTTCGAGCCCCCAACTTTCGTTCTTGATTAATGAAAACATCCTTGACAAATGCTTTCGCAGAAGTTCGTCTTTCATAAATCCAAGAATTTCACCTCTGACAATGAAATACGAATGTCCCCGACTGTCCCTCTTAATCATTACTCTGGTCTCACAGACCAACAAGATAAGCCAGAGTCCTATCGTGTTATTCCATGCTAATATATTCAGAGCGTAGGCTTGCTTTGAACACTCTAATTTACTCAAAGTAACTTCGTCGGCTCCCAGTCCCGGACAGTGAAGCCCAGGAGCTGGTCCCCGACAACAAGATAGGACCTACCAGTACACATCTCAAAGACGGACCGGCAGATCCCATCCGAAATCCAACTACGAGCTTTTTAACTGCAACAACTTAAATATACGCTATTGGAGCTGGAATTACCGCGGCTGCTGGCACCAGACTTGCCCTCCAATGGATCCTCGTTAAGGGATTTAGATTGTACTCATTCCTATTACCAGACTTAAGAAGCCCGGTATTGTTATTTATTGTCACTACCTCCCTGAATTAGGATTGGGTAATATTACGCGCCTGTTAATCTCTAGAGGATCCCCGGGTACCGAGCTCGAATTCGTAATCATGGTCATAGCTGTTTCCTGTGAAAATTGTTATCCGCTCACAATTCCACACAACCATACGAGCTCGGAAGCATATAGTGTAAAGCTGGGGGTGCCTT

>24-148

AGCAGGCGCGTAAATTGCCCAATCTTGACACAGGGAGGTAGTGACAATAAATAACAATGCCGGGCCATCATTGGTCTGGCAATTGGAATGAGAACAATTTAAATCCCTTATCGAGGATCAATTGGAGGGCAAGTCTGGTGCCAGCAGCCGCGGTAATTCCAGCTCCAATAGCGTATATTAAAGTTGTTGCAGTTAAAAAGCTCGTAGTTGGATTTGTGGCGTACGGTGTGTTCCGGGCACTTGTTGTCTGAGTAACCCGCTGTTGCCATCCTTGGGTGGAACCTGTGTGGCATTAGGTTGTCGTGCAGGGGATGCCCATCATTTACTGTGAAAAAATTAGAGTGTTCAAGGCAGGCTCTCGCCTGAATACATTAGCATGGAATAATGGAAGAGGACCTCGGTCCTATTTTGTTGGTTTCTAGGGCCGAAGTAATGATTAAGAGGGACAGTTGGGGGCATTCGTATTTAATTGTCAGAGGTGAAATTCTTGGATTTATGAAAGACGAACTAGTGCGAAAGCATTTACCAAGGATGTTTTCATTAATCAAGAACGAAAGTTAGGGGATCGAAGATGATTAGATACCATCGTAGTCTTAACCATAAACTATGCCGACAAGGGATTGGCGGAGTTTCGTTATGTCTCCGTCAGCACCTTATGAGAAATCACAAGTCTTTGGGTTCCGGGGGGAGTATGGTCGCAAGGCTGAAACTTAAAGAAATTGACGGAAGGGCACCACCAGGAGTGGAGCCTGCGGCTTAATTTGACTCAACACGG

>24-149

AGCAGGCGCGCAAATTGCCCAATCCCGACTCGGGGAGGTAGTGACAATAAATAACAATGCCGGGCTATTGTAGTCTGGCAATTGGAATGAGTACAATCTAAATCCCTTAACGAGGAACAATTGGAGGGCAAGTCTGGTGCCAGCAGCCGCGGTAATTCCAGCTCCAATAGCGTATATTAAAGTTGTTGCAGTTAAAAAGCTCGTAGTTGGATTTCGGGGTGGCCTGCCTGGCCCACCGCAAGGTGAGTACTGGTCAGCCGCCCTTCCTCTCGAAAGCCCCGACTGCTCTTCATTGCAGTGGTCGGGTAGTTCGGGACGTTTACTTTGAAAAAATTAGAGTGTTCAAGGCAGGCTCTCGCCTGAATACATTAGCATGGAATAATGGAAGAGGACCTCGGTCCTATTTTGTTGGTTTCTAGGGCCGAAGTAATGATTAAGAGGGACAGTTGGGGGCATTCGTATTTAATTGTCAGAGGTGAAATTCTTGGATTTATGAAAGACGAACTAGTGCGAAAGCATTTGCCAAGGATGTTTTCATTAATCAAGAACGAAAGTTAGGGGTTCGAAGACGATCAGATACCGTCGTAGTCCTAACCATAAACTATGCCGACTAGGGATCGGTGGATGTTAATGTTTGACTCCATCGGCACCTTATGAGAAATCAAAGTCTTTGGGTTCCGGGGGGAGTATGGTCGCAAGGCTGAAACTTAAAGGAATTGACGGAAGGGCACCACCAGGAGTGGAGCCTGCGGCTTAATTTGACTCAACACGG

>24-150

AGCAGGCGCGCAAATTGCCCAATCTCAGAACGAGGAGGTAGTGACGAAAAATAACGAGACAGTCTTCTATGAGGTCTGTCATCGGAATGGGTACAATTTAAACCCTTTAACGAGGATCTATTGGAGGGCAAGTCTGGTGCCAGCAGCCGCGGTAATTCCAGCTCCAATAGCGTATATTAAAGTTGTTGCGGTTAAAAAGCTCGTAGTTGGATCTGCGTCCATCAAGACCGGTAGCCCATTGGGTTTCACTGGTTCTTGTGGACTCCTTACTAGTTTCTCCATGGTGCTCTTAACTGAGTGCCTTGGTAGACTAGTGCTTTTACTTTGAAAAAATTAGAGTGCTCAAAGCAGGCGTTTGCCTGAATAGTGGTGCATGGAATAATGGAATAGGACCTCGGTTCTGTTTTGTTGGTTTTCGGAGCTCGAGGTAATGATTAAGAGGGACAGACGGGGGCATTCGTACTGCTGCGTTAGAGGTGAAATTCTTGGATCGCAGCATGACGAGCAACTGCGAAAGCATTTGCCAAGAATGTTTTCATTAATCAAGAACGATAGTTAGAGGTTCGAAGGCGATCAGATACCGCCCTAGTTCTAACCGTAAACGATGCCAACTAGCAATCCGCTGGTGTTAATTTTGGCTCAGCGGGCGGCTGCCCGGAAACGATAAGTTTTTCGGTTCCGGGGGAAGTATGGTTGCAAATCCGAAACTTAAAGGAATTGACGGAAGGGCACCACCAGGAGTGGAGCCTGCGGCTTAATTTGACTCAACACGG

>24-151

AGCAGGCGCGCAAATTGCCCAATCTCGACGCGGGGAGGTAGTGACAATAAATACCGATGCAGGGCCCTTATGGGTCTTGTAATTGGAATGAGTACAATTTAAATCCCTTAACGAGGAACAATTAGAGGGCAAGTCTGGTGCCAGCAGCCGCGGTAATTCCAGCTCTAATAGCGTATATTAAAGTTGTTGCAGTTAAAAAGCTCGTAGTTGAACCTTGGGCCTGGCTGGCCGGTCCGCCTCACCGCGTGCACTGGTCCGGCCGGGCCTTTCCTTCTGGGGATCCGCATGCCCTTCACTGGGCGTGTCGGGGAACCAGGACTTTTACTTTGAAAAAATTAGAGTGTTCAAAGCAGGCCTTTGCTCGAATACATTAGCATGGAATAATAGAATAGGACGTGTGGTTCTATTTTGTCGGTTTCTAGGACCGCCGTAATGATTAATAGGGATAGTCGGGGGCATCCGTATTCAATTGTCAGAGGTGAAATTCTTGGATTTATTGAAGACGAACTACTGCGAAAGCATTTGCCAAGGATGTTTTCATTAATCAGTGAACGAAAGTTAGGGGATCGAAGACGATCAGATACCGTCGTAGTCTTAACCATAAACTATGCCGACTAGGGATCGGTGGATGTTATCTTTTTGACTCCATCGGCACCTTACGAGAAATCAAAGTTTTTGGGTTCTGGGGGGAGTATGGTCGCAAGGCTGAAACTTAAAGAAATTGACGGAAGGGCACCACCAGGCGTGGAGCCTGCGGCTTAATTTGACTCAACACGG

>24-154

AGCAGGCGCGTAAATTGCCCAATCCTGACACAGGGAGGTAGTGACAATAAATAACAATGCCGGGCCTTTAAAGGTCTGGCAATTGGAATGAGAACAATTTAAACCCCTTATCGAGGATCAATTGGAGGGCAAGTCTGGTGCCAGCAGCCGCGGTAATTCCAGCTCCAATAGCGTATATTAAAGTTGTTGCAGTTAAAAAGCTCGTAGTTGGATTTGTGGTTGAGGTGGCCGGCCCTCACTATGTGTGGTGCTTGGCTGTCCTCGTCCATCCTTGGGTGGAACTGGTGTGGCATTAAGTTGTCGTGTCAGGGATGCCCATCATTTACTGTGAAAAAATTAGAGTGTTCAAAGCAGGCTTACGCCGCTGAATATATTAGCATGGAATAATGAGATAGGACCTTGGTACTATTTTGTTGGTTTGCGCACCGAGGTAATGATTAATAGGGACAGTTGGGGGTATTCGTATTCCATTGTCAGAGGTGAAATTCTTGGATTTCTGGAAGACGAACTACTGCGAAAGCATTTACCAAGGATGTTTTCATTAATCAAGAACGAAAGTTAGGGGATCGAAGATGATTAGATACCATCGTAGTCTTAACCATAAACTATGCCGACAAGGGATTGGCGGGCGTTGTATTGACTCCGTCAGCACCTTATGAGAAATCACAAGTCTTTGGGTTCCGGGGGGAGTATGGTCGCAAGGCCGAAACTTAAAGAAATTGACGGAAGGGCACCACCAGGAGTGGAGCCTGCGGCTTAATTTGACTCAACACGG

引物：primer 18S Non-copepodF2 (5’-agcaggcgcGhaaattrcccaatcy-3’) paired with 18S Non-copepodR2 (5’-CCGTGTTGAGTCAAATTAAGCCG-3’CGGCTTAATTTGACTCAACACGG)
